# Deciphering the Chameleonic Chemistry of Allenols: Breaking the Taboo of a Onetime Esoteric Functionality

**DOI:** 10.1021/acs.chemrev.0c00986

**Published:** 2021-02-25

**Authors:** José M. Alonso, Pedro Almendros

**Affiliations:** † Grupo de Lactamas y Heterociclos Bioactivos, Departamento de Química Orgánica, Unidad Asociada al CSIC, Facultad de Química, Universidad Complutense de Madrid, 28040 Madrid, Spain; ‡ Instituto de Química Orgánica General, IQOG-CSIC, Juan de la Cierva 3, 28006 Madrid, Spain

## Abstract

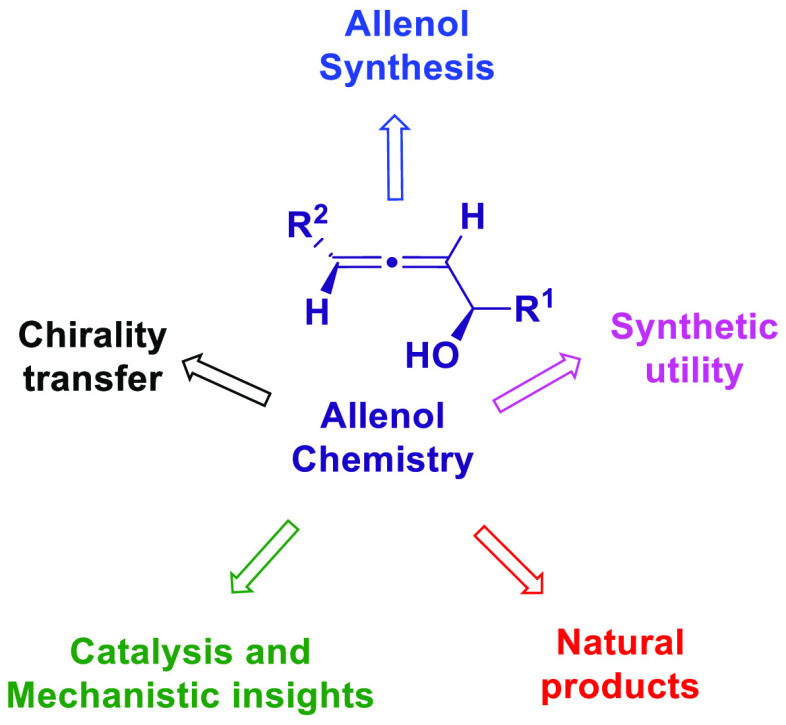

The allene functionality has participated in one of the most exciting voyages in organic chemistry, from chemical curiosities to a recurring building block in modern organic chemistry. In the last decades, a special kind of allene, namely, allenol, has emerged. Allenols, formed by an allene moiety and a hydroxyl functional group with diverse connectivity, have become common building blocks for the synthesis of a wide range of structures and frequent motif in naturally occurring systems. The synergistic effect of the allene and hydroxyl functional groups enables allenols to be considered as a unique and sole functionality exhibiting a special reactivity. This Review summarizes the most significant contributions to the chemistry of allenols that appeared during the past decade, with emphasis on their synthesis, reactivity, and occurrence in natural products.

## 1 Introduction

Allenes are far considered the most useful and widely employed of the cumullenes. Since Van’t Hoff’s early predictions about structure and composition,^1^ chemists have produced a continuous stream of research in the allene field, facilitated by the perfect balance of reactivity and stability in the allene unit.^2−13^ Opposite to alkynes or alkenes, allenes show three reaction sites coupled to potential axial chirality. They can behave both as electrophiles and nucleophiles,^14−17^ and they can undergo cycloaddition reactions,^18−21^ thermal or radical rearrangements.^22,23^ Besides their synthetic utility, they are also recurring subjects in catalysis and theoretical studies.

In the last decades, a special kind of allene, namely, allenol, has emerged in both organic and physical organic chemistry, becoming a common building block for the synthesis of a wide range of structures..^24−36^ Allenols are formed by an allene and a hydroxyl functional group showing diverse connectivity. The synergistic effect of one functional group over the other when sharing the same skeleton makes the allenol unit a unique and sole functional group exhibiting a special reactivity. In one hand, allenols can be viewed as π-activated alcohols showing an extra reactivity toward eliminations, substitutions, or rearrangements. On the other hand, they can be viewed as allenes bearing extra electron pairs, which promote intramolecular cyclizations or provide an alternative metal-coordination site. This overview is focused in the most recent examples dealing with the enhanced chemical behavior of allenols, leaving aside the more particular situations where the allene and alcohol motifs react separately within the allenol molecules.

Herein we will discuss the most significant contributions to the allenol chemistry appeared during the past decade. Nevertheless, selected early works will be also mentioned to keep a critic and accurate review about the history of allenols. First of all, we will describe the most representative advances for the synthesis of allenols, specially focusing in the more challenging highly substituted structures and chiral allenes. Also, the different connectivity between the allene and hydroxyl moieties leading to α-, β-, γ-, or δ-allenols will be detailed. In a second chapter, synthetic utility of the allenol functional group will be discussed. This chapter will be organized according to the diverse reactivity of the allenol skeleton. Thus, in a first section, allenols as special π-activated alcohols will be considered, mainly showing hydroxyl units as leaving groups through elimination, substitution, aromatization, or rearrangement processes. A second section will describe the bidentate nature of the allenol, acting both as nucleophile and electrophile in annulation reactions. A third section is dedicated to all the examples where the OH group is not leaving or attacking the cumullene bonds. Instead, the alcohol unit is acting as a coordination site facilitating diverse transformations as additions, bond migrations, or isomerizations. A final section will contemplate the most recent achievements in chirality transfer using allenols through any kind of transformation. In addition, and to proof the extensive use of allenols in every field related to organic chemistry, a last chapter will be considered discussing the last contributions in natural product chemistry. Both allenols as key reaction intermediates and as motifs in the final structure will be presented.

## 2 Synthesis of Allenols

The extensive use of allenols in organic chemistry has also facilitated a considerable number of methodologies for their preparation. In addition to the most conventional routines, such as the allenation of terminal alkynes or the metal catalyzed addition of propargyl derivatives to aldehydes, different strategies have emerged during the past decade to provide more complex structures through more creative procedures. Among the diverse family of allenols, those bearing the hydroxyl unit at the α position, namely α-allenols, represent the widest number and focus the main part of investigations regarding both synthesis and applications. On the other hand, the synthesis of allenols exhibiting different connectivities such as β-, γ-, or δ-allenols have been frequently described following adapted methodologies from α-allenol synthesis. Thus, to get a concise discussion with a more homogeneous distribution, a classification according to the synthethic strategy is herein presented. Moreover, differently substituted allenols in both racemic and enantioenriched versions will be discussed.

### 2.1 Racemic Allenols

**Scheme 1:**
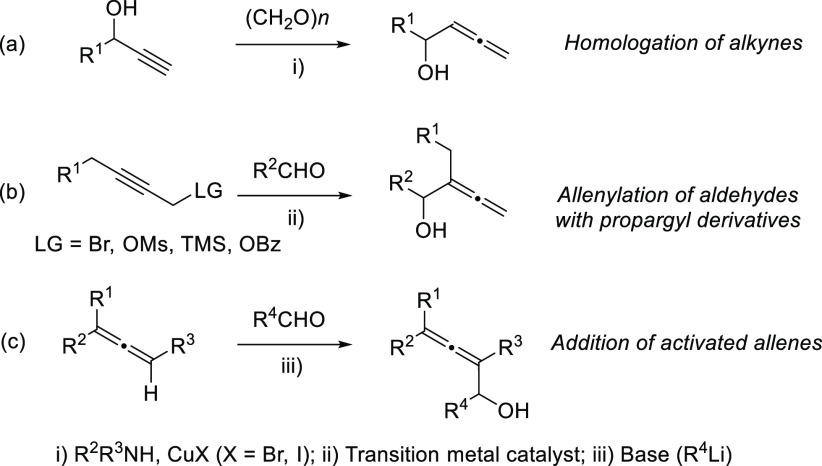
Classical Strategies for the Synthesis of Racemic Allenols

The most classical methodologies for the synthesis of racemic allenols start from alkynes, mainly including homologation of propargylic alcohols and addition of propargyl bromides to aldehydes (Scheme 1, reactions a and b). Also, activated allenes have been often used as starting materials in the aldol-type addition to carbonyl compounds (Scheme 1, reaction c).

**Scheme 2:**
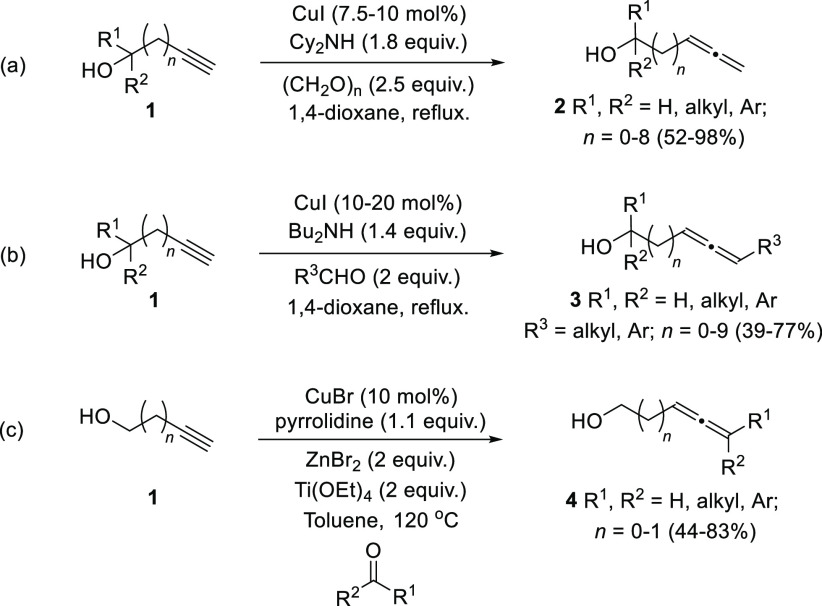
Optimization of the Allenation of Terminal Alkynes for the Synthesis of Substituted Allenols

Among the homologation procedures, allenation of terminal alkynes from propargylic alcohols is still one of the most common methodologies for the construction of the allenol skeleton. In the late 70s, Crabbé and co-workers reported the first synthesis of allenes from terminal alkynes, isopropylamine, paraformaldehyde, and CuBr as metal catalyst.^37−39^ The presence of a hydroxyl group in the alkyne unit seemed to activate the transformation providing α-allenols with excellent yields. Nevertheless, this transformation was limited to paraformaldehyde leading only to monosubstituted allenols as reaction products. In recent years, Ma research group has extensively investigated the scope of the allenation of terminal alkynes toward the synthesis of di- and trisubstituted allenes, by extending the methodology to diverse aldehydes and ketones.^40^ Thus, yields and scope were first improved by changing the original CuBr/propylamine pair for CuI/cyclohexylamine toward the optimized synthesis of terminal allenes **2**. This methodology was successfully applied for the preparation of different allenols, such as β- or γ-allenols, normally showing low yields under original Crabbé’s reaction conditions (Scheme 2, reaction a).^41−46^ To extend the reaction to normal aldehydes, the same research group assumed that finding the proper metal salt/secondary amine combination should be the key for the direct allenylation. Fortunately, diverse matching combination such as ZnI_2_/morpholine or CuI/Bu_2_NH gave successful results from different starting materials (Scheme 2, reaction b); 1,3-disubstituted allenols **3** were therefore accessible.^47,48^ Further extension to trisubstituted allenols **4** was achieved by reaction with ketones using CdI_2_/pyrrolidine or the less toxic CuBr-ZnBr_2_/pyrrolidine reagent combination in a sequential addition procedure, or CuBr-ZnBr_2_–Ti(OEt)_4_/pyrrolidine in the one pot version (Scheme 2, reaction c).^49−51^

**Scheme 3:**
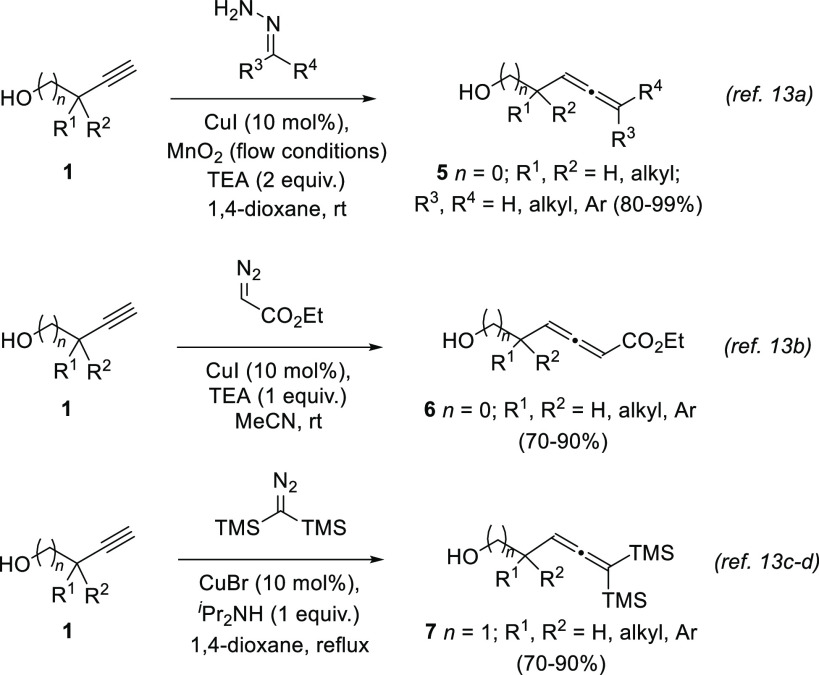
Substituted α- and β-Allenols from Terminal Alkynes and Diazo Compounds

An alternative approach for the homologation of terminal alkynes under copper catalysis employs differently substituted diazo compounds instead of aldehydes. First described by Wang and collaborators,^52^ this methodology has also been applied for the synthesis of substituted allenols by diverse research groups (Scheme 3).^53−56^ Alkyl and aryl-substituted allenes, allenoates, and TMS-disubstituted allenes are described in the recent literature following this strategy. In addition, both α- and β-allenols are accessed with no considerable decrease of yield.

Allenylation of aldehydes with propargyl derivatives bearing an appropriate leaving group (normally bromides) represent another classical approach for the allenol synthesis. Many reports have appeared dealing with the regioselective control between propargylation and allenylation, including a wide variety of metal catalysts, such as Sn complexes,^57−59^ Zn,^60−62^ Bi, or Cd salts.^63^ Among all these well-known procedures, In-promoted allenylation in aqueous media has possibly provided the best results.^64−67^ In this field, Cr-catalyzed allenylation of aldehydes is probably the only contribution appeared during the past decade, allenols are prepared in a complete regioselective manner allowing both racemic and enantioselective synthesis, with the late being further discussed in the next section.^85^

**Scheme 4:**
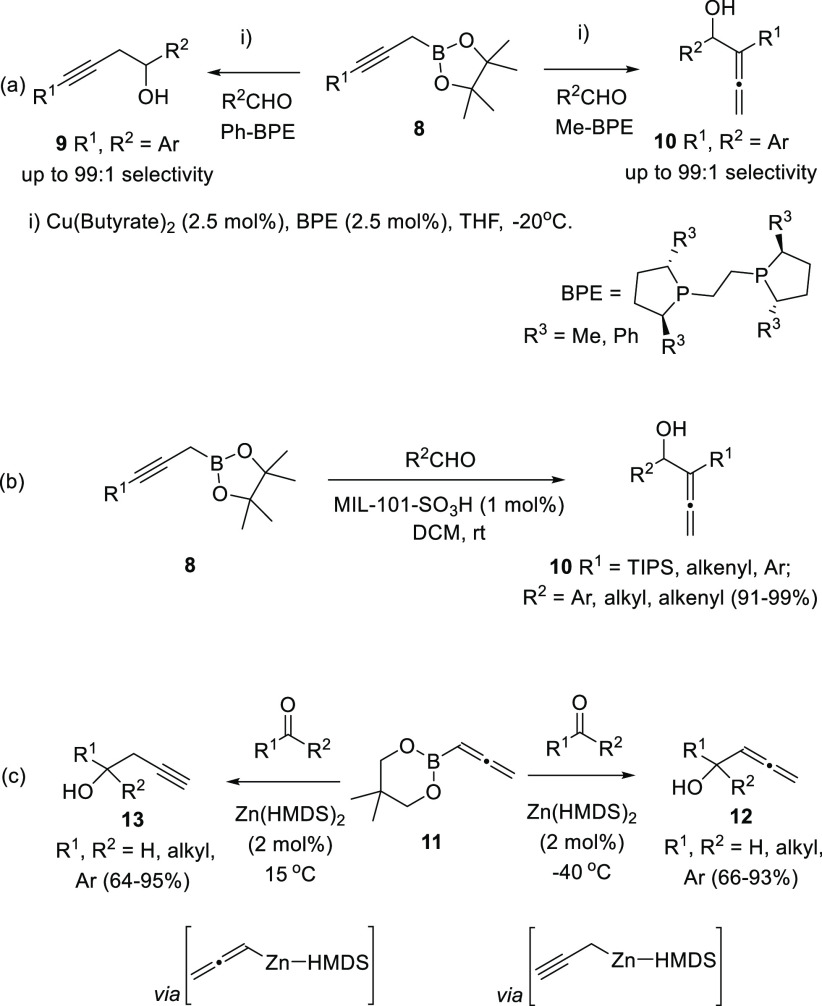
α-Allenol Synthesis through Propargylboronic Addition onto Carbonyl Compounds

In a similar approach, propargyl boronic esters have recently shown a good control in the regioselectivity toward the addition to aldehydes. Copper catalysis allowed the synthesis of both alkynol **9** and α-allenol structures **10** by switching the phosphine-based ligand (Scheme 4, reaction a).^69,70^ This study has been analyzed from both experimental and theoretical perspectives. Alternatively, the MOF (metal–organic framework)-supported mineral acid catalyst MIL-101 yielded similar α-allenols **10** as sole reaction products through a related transformation (Scheme 4, reaction b).^71^

Kobayashi and co-workers have also reported an example of regiocontrol toward the allenylation *versus* propargylation of both aldehydes and ketones from boronic esters. In this case, Zn-propargyl or Zn-allenyl species are generated *in situ* from allenyl-boronic derivatives **11**. The regioselectivity of the process was found to be temperature-dependent, yielding α-allenol species **12** as major reaction products at lower temperatures (Scheme 4, reaction c).^72^

**Scheme 5:**
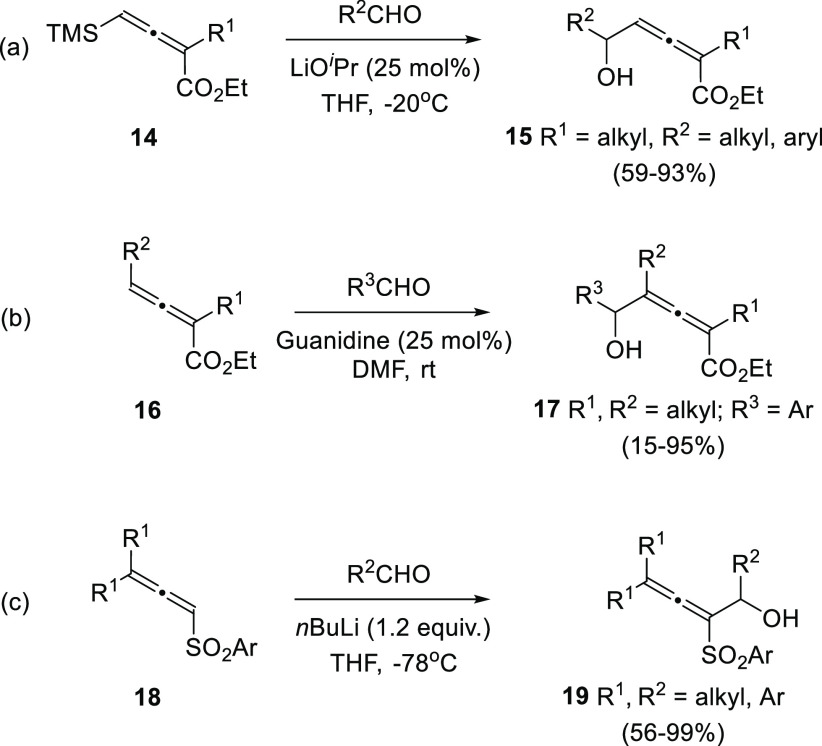
α-Allenol Synthesis through Activated Allene Addition to Aldehydes

Direct allene addition onto carbonyl compounds have also provided several examples of α-allenol synthesis through allene-aldol or Baylis-Hillman-type reactions, including both α- and γ-selective additions.^73−75^ In the recent literature, γ-addition has been reported from substituted allenoates **14** and **16** and diverse aldehydes through Morita-Baylis-Hillman additions using different catalysts (Scheme 5, reactions a and b).^76,77^ Direct addition of allenes to carbonyls frequently shows scope limitation as activated substrates are needed, normally allenoates. One rare example of this kind of transformation using a different activated allene employs tosyl derivatives **18**. Treatment with *n*BuLi at low temperature yields the corresponding organolithium compound, which is reported to be trapped in the presence of different aldehydes generating α-allenols **19** (Scheme 5, reaction c).^78^ A wide variety of aldehydes or ketones and arylsulfones are tolerated, although the challenging trisubstitution pattern around the allene skeleton should be already present in the starting material.

**Scheme 6:**
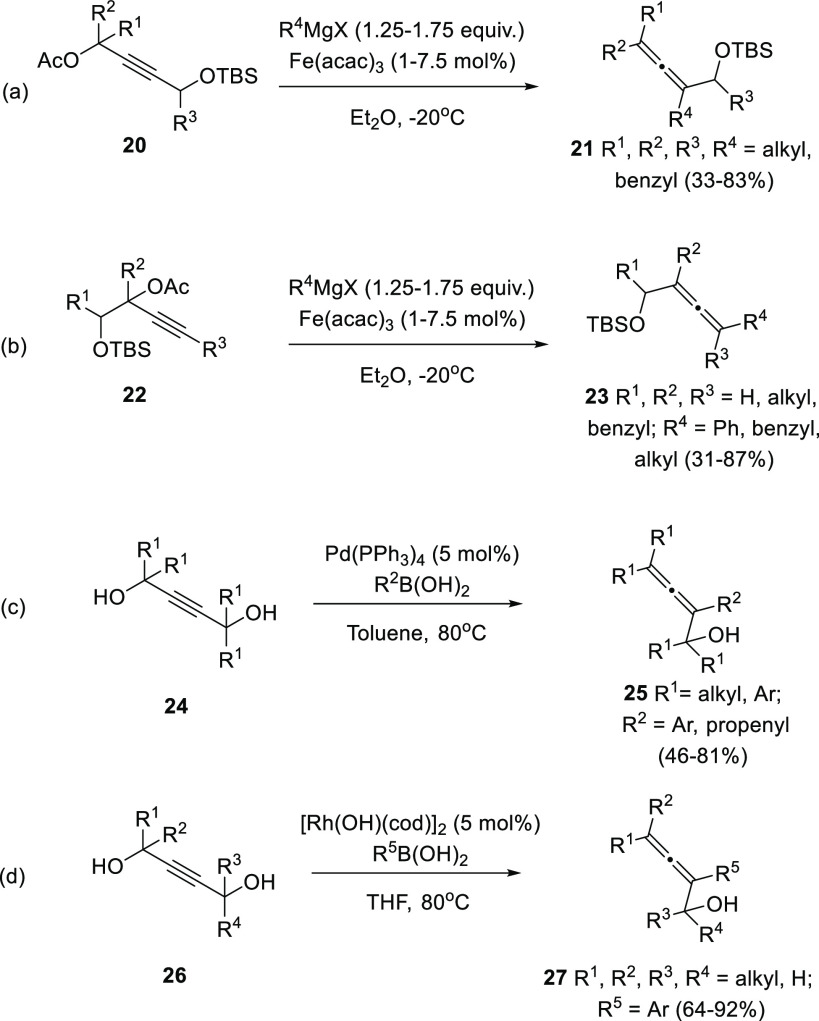
Substituted α-Allenols from Alkyndiols

Besides the above-mentioned general strategies for the racemic allenol synthesis, the past decade has witnessed an increasing number of more specific transformation leading to more challenging α-allenol structures. Bäckvall research group has employed protected alkyndiols **20** and **22** through an iron-catalyzed cross-coupling reaction with Grignard reagents for the synthesis of di-, tri- and tetra-substituted allenols **21** and **23** (Scheme 6, reactions a and b).^79^ Acetate-protected hydroxyls act as the leaving group facilitating the cumullene generation, while TBS-protected OH remains unaltered in the final allenol skeleton. The two alcohol units could be placed in either opposite (**20**) or same (**22**) side of the alkyne moiety, providing a wider scope and versatility. Also, the mild reaction conditions allowed an extensive functional group compatibility in both the alkyndiol system and the Grignard reagent. Related work from Sherburn’s and Dou’s research groups have independently shown alkyndiol efficiency in the allenol synthesis (Scheme 6, reactions c and d). Pd-catalyzed Suzuki-Miyaura cross-coupling reaction from symmetrically substituted alkynes **24** allowed the synthesis of allenes **25**. Nevertheless, only sterically hindered boronic acids were tolerated to avoid 2-fold addition processes (Scheme 6, reaction c).^80^ The use of rhodium catalysis in a similar transformation from diols **26** provided higher control toward the single addition reaction. Less hindered boronic acids were allowed, and unsymmetrically substituted alkyndiols **26** were also tolerated under Rh conditions (Scheme 6, reaction d).^81^

**Scheme 7:**
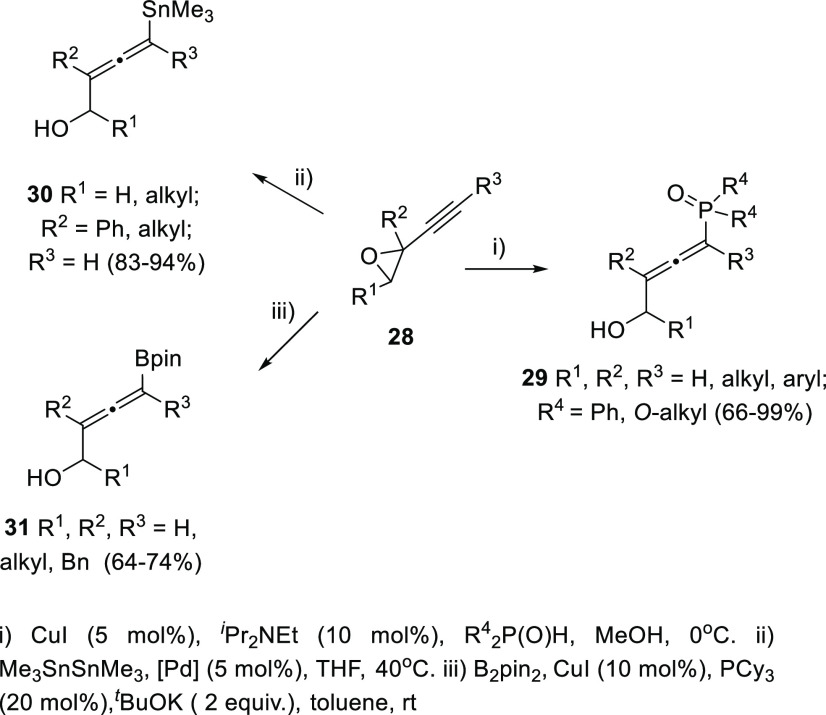
Allenol Synthesis through Selective Ring Opening of Propargyl Epoxides

Propargyl epoxides **28** have also been employed for the synthesis of substituted α-allenols through S_N_2′-type reactions in the presence of nucleophiles.^82−87^ While C-based nucleophiles such as Grignard reagents have been previously reported in classic methodologies, heteronucleophiles are much more scarcely described.^88−91^ Nevertheless, recent publications have started to focus in this transformation for the synthesis of allenes showing carbon-heteroatom bonds, not easily accessible through any other approach. Thus, B-,^92^ P-,^93^ and Sn-decorated allenols **29**–**31**,^94−96^ have been synthesized using different transition metals as catalysts and mild reaction conditions (Scheme 7).

**Scheme 8:**
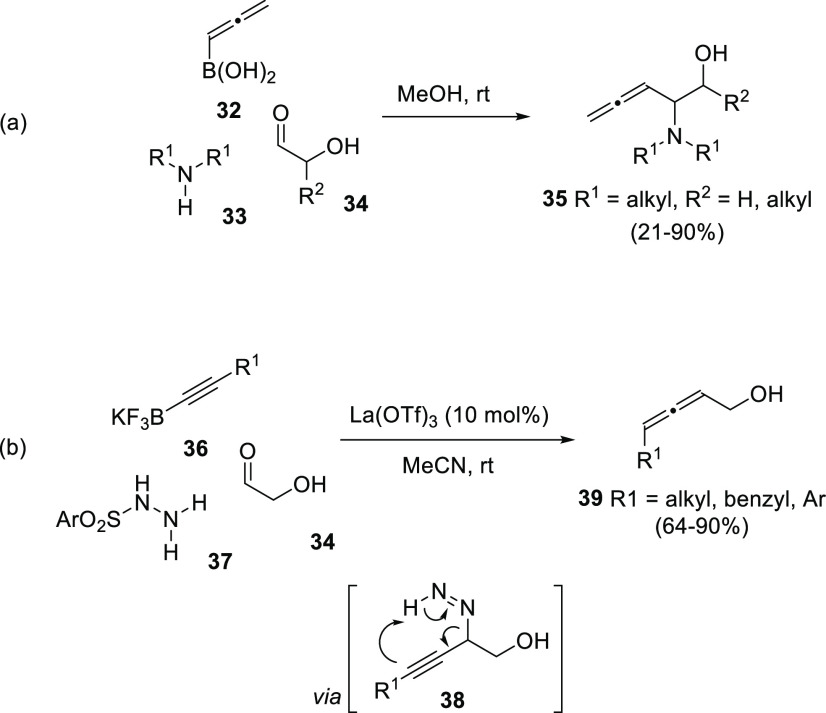
Multicomponent Strategies for Allenol Synthesis

Multicomponent reactions allow the synthesis of highly substituted and complex structures in one single step. This approach has been recently applied to the synthesis of β- and α-allenols from allenyl or propargyl boronic compounds, respectively. Petasis and co-workers have reported the synthesis of allenyl aminoalcohols **35**, exhibiting a β-allenol motif, by a metal-free three-component process using allenyl boronic acids **32**, amines **33**, and hydroxyaldehydes **34**.^97^ Regioselectivity (allenylation *vs* propargylation) was found to be dependent on the amine. Thus, secondary aliphatic amines selectively furnished the corresponding allenols (Scheme 8, reaction a), while primary and aromatic amines yielded the propargylation products. Alternatively, Thomson research group described a multicomponent reaction from alkynyl trifluoroborate salts **36**, hydroxyaldehydes **34**, and sulfonylhydrazines **37**.^98^ The strategy was based in the *in situ* decomposition of the intermediate propargyl diazine **38** to yield the allenol compound **39** as sole reaction product through a so-called traceless Petasis-type process (Scheme 8, reaction b).

More particular transformations to yield the allenol motif include the synthesis of exocyclic allenols through carbocyclization of both allenynes,^99^ or carbonyl enynes,^100,101^ the deoxygenation of pentadiyn diols,^102^ the aza-Cope-type rearrangement of propargyl indoles,^103^ one example of ethynylation and S_N_2′ reaction,^104^ or transformations from 1,3-enynes, such as alkylarylations,^105^ or hydromagnesiation.^106^

### 2.2 Enantioenriched Allenols

Asymmetric synthesis and chirality transfer processes have attracted much attention during the last years. Enantioenriched starting materials as chirality transfer agents represents one of the most common approaches. Thus, great interest has been shown in the design and synthesis of enantioenriched allenols, useful building blocks in asymmetric synthesis through diverse transformations as it will be later detailed. Because of the orthogonal distribution of cumullene molecular orbitals, allenes exhibit axial chirality when differently substituted. In addition, the presence of the extra alcohol unit in the allenol skeleton provides a potential stereogenic center. Synthesis of enantioenriched allenols may therefore contemplate axial chirality generation, central chiral generation, or both in the most complex cases. The principal methodologies for the synthesis of enantioenriched allenols that will be discussed in this section may be divided in three general groups; chirality transfer from enantioenriched starting materials, asymmetric synthesis using enantiopure catalysts, and dynamic resolution of racemic allenols.

#### 2.2.1 Chirality Transfer and Chirality Induction from Enantioenriched Starting Materials

##### 2.2.1.1 Allenols Showing Axial Chirality and Axial-Central Chirality

**Scheme 9:**
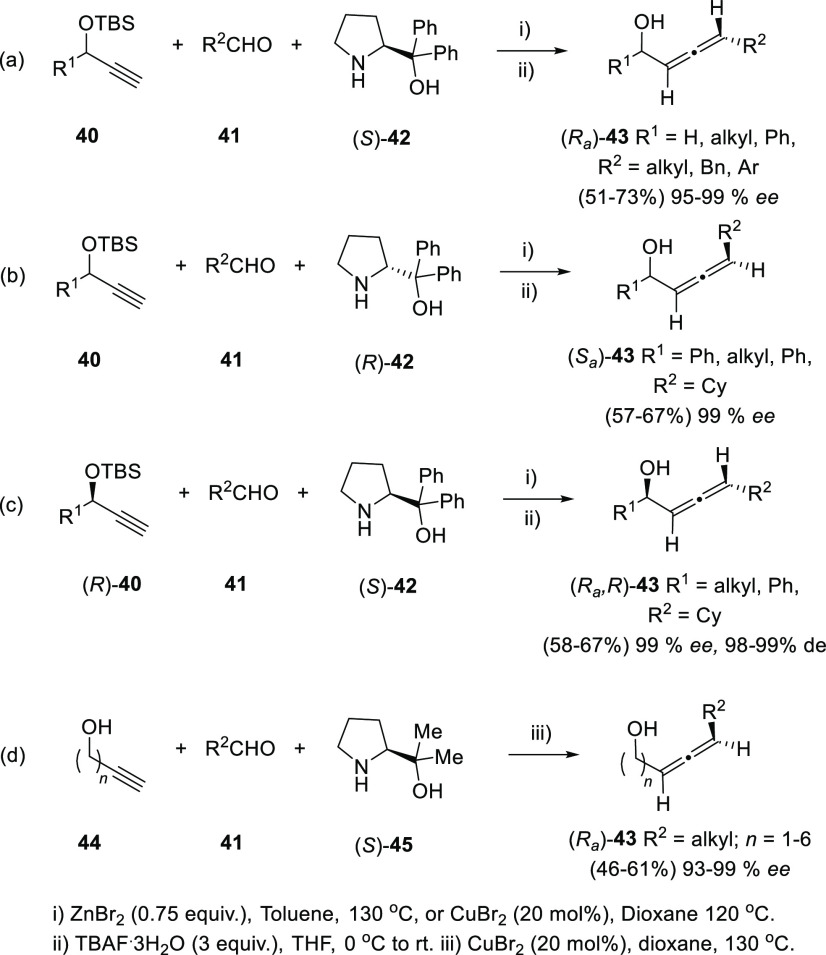
Asymmetric Allenation of Terminal Alkynes Employing Chiral Amines

Allenation of terminal alkynes of terminal alkynols has also been investigated in the asymmetric version to yield optically pure allenols. Ma and co-workers have employed differently substituted prolinols **42** and **45** as chirality transfer agents providing practical yields and good to excellent enantioselectivities. TBS-protected alkynols **40** were first explored in combination with both (*R*)- and (*S*)-diphenylprolinol (**42**). Axial enantioselectivity is perfectly controlled from the absolute configuration of the amines **42** (Scheme 9, reactions a and b), while the stereochemistry of the hydroxyl carbon in the starting alkynol **40** is retained throughout the reaction when enantioenriched substrates were tested (Scheme 9, reaction c). The authors also point that the bulky TBS group in the alkynol **40** may have double role by avoiding the allene racemization and enhancing the enantioselectivity.^107,108^ In a later work, dimethylprolinol (**45**) was found to exhibit higher enantiodirection, allowing the use of unprotected alkynols **44** as starting materials and extending the scope to the obtention of β-, γ-, and δ-allenols (Scheme 9, reaction d).^109^

**Scheme 10:**
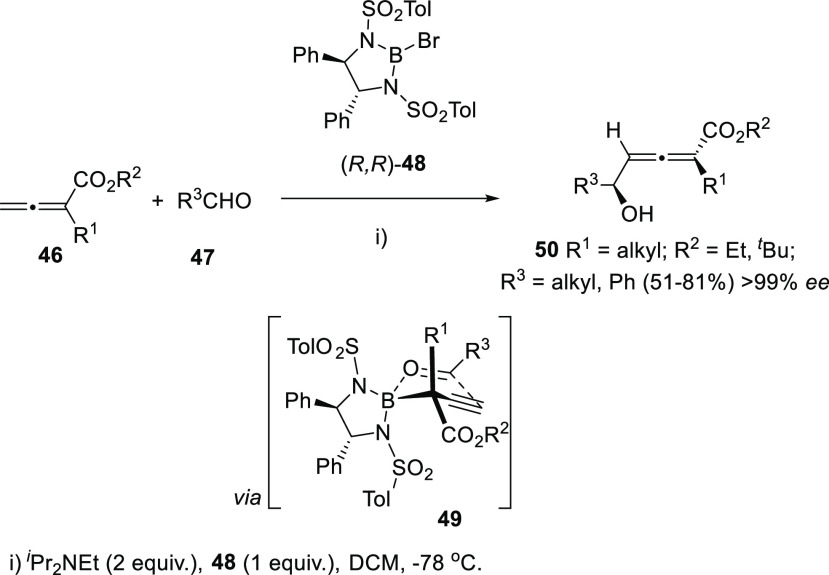
Aldol Allenoate Addition to Aldehydes Promoted by Chiral Bromoboranes

Yu’s research group has envisioned an aldol allenoate addition to aldehydes **47** promoted by chiral bromoboranes **48** in the presence of tertiary amines. Both regiochemical and stereochemical outcomes of the reaction are explained through a Curtin-Hammet-type transition state **49**, selectively favoring γ-addition products **50** and providing excellent chiral and central enantioselectivities (Scheme 10). The methodology was also applied for the kinetic resolution of racemic aldehydes, and further generation of the butenolide core of the natural product (+)-xilogiblactone A.^110−113^

**Scheme 11:**
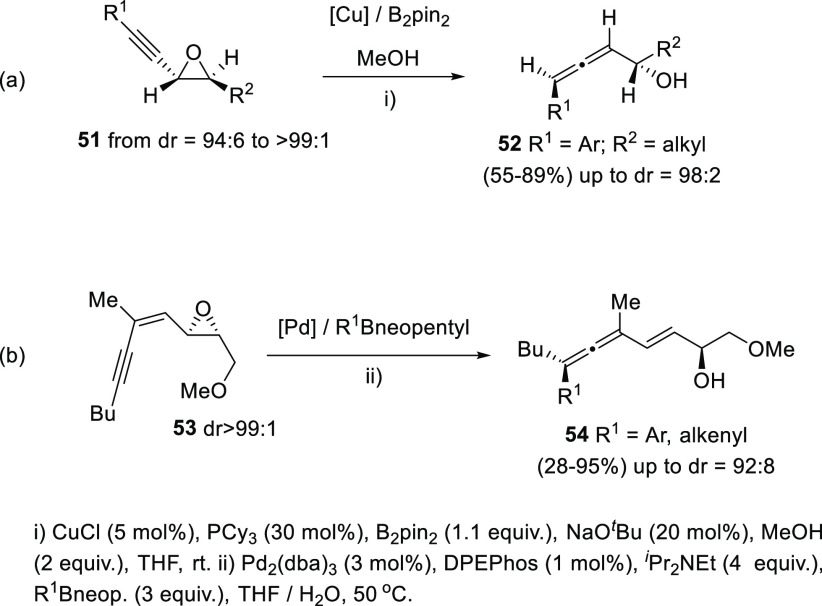
Chiral Oxiranes as α- and γ-Allenol Precursors

Enantioenriched oxiranes have also been employed for the synthesis of di- and trisubstituted allenols with good diastereoselectivites.^114−116^ Two related approaches based in metal catalysis and organoboron reagents have been described. Propargyl epoxides **51** undergo a ring-opening through a *syn*-hydride borane addition using MeOH as proton shuttle, followed by selective *syn*-elimination catalyzed by copper salts. The proper phosphine-base combination seemed to be crucial for the axial selectivity toward allenols **52** (Scheme 11, reaction a).^117^ On the other hand, enynyl oxiranes **53** have been reported to react through a formal S_N_2′ mechanism in the presence of aryl boronic esters and palladium catalysts to provide enantioenriched allenols **54** (Scheme 11, reaction b).^118^

**Scheme 12:**
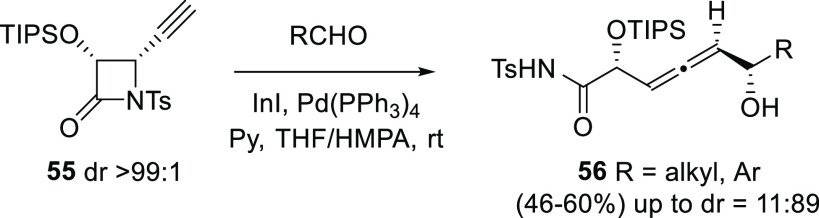
Central-to-Axial Chirality Transfer from Ethynyl β-Lactams

Another example of a central-to-axial chirality transfer uses optically pure ethynyl β-lactams **55** and different aldehydes for the asymmetric synthesis of structurally complex allene diols **56**. The process includes initial *in situ* generation of the propargyl indium reagent, and further addition onto aldehydes. Although complete selectivity in the generation of the new α-hydroxy chiral center was not achieved, a reasonable dr = 11:89 could be attained (Scheme 12).^119^

**Scheme 13:**
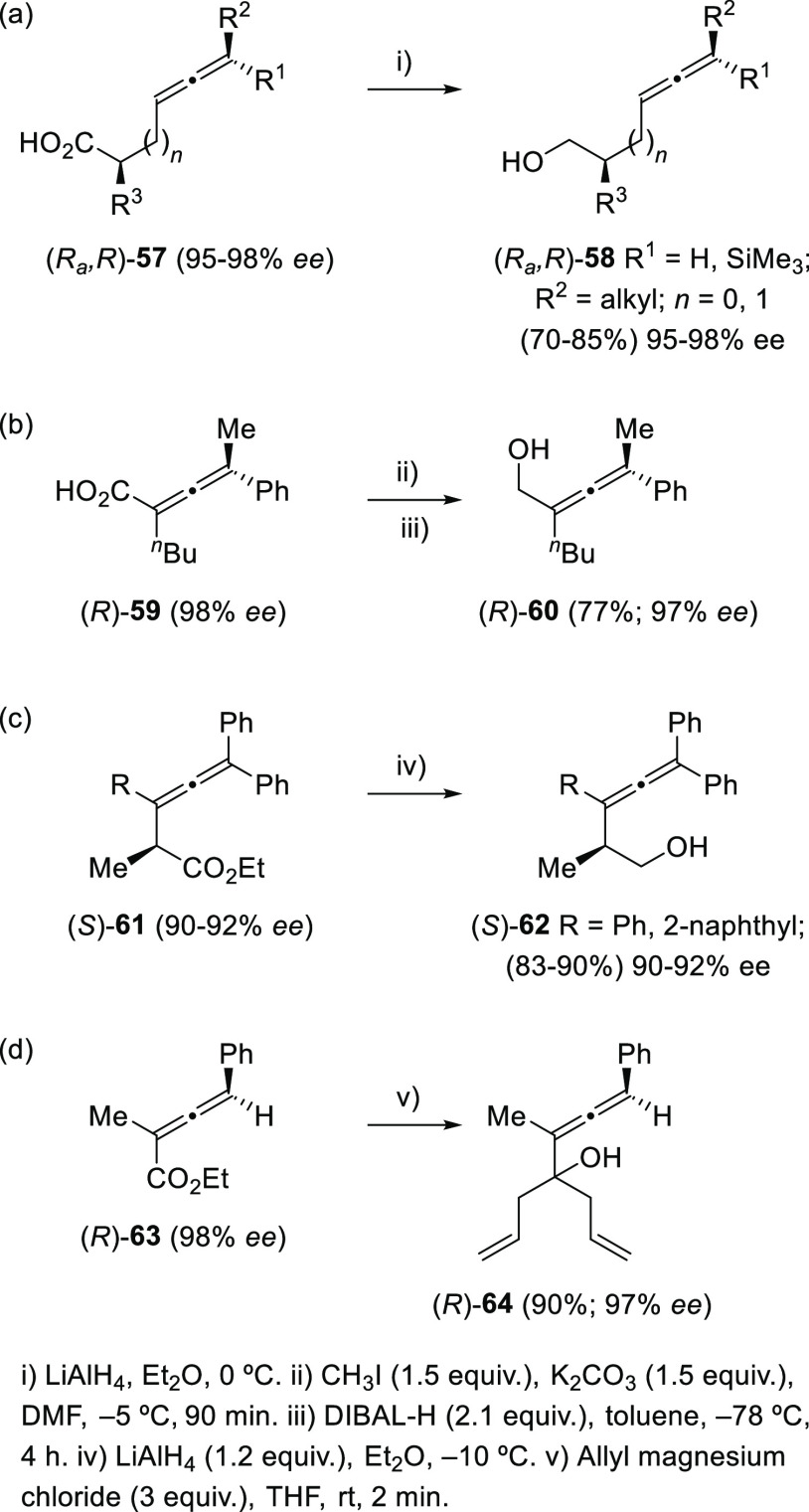
Chiral Allenoic Acids and Allenoates as α-, β-, and γ-Allenol Precursors

Ma and colleagues have accomplished the synthesis of enantioenriched β- and γ-allenols **58** taking advantage of the reduction of optically pure allenoic acids **57** with LiAlH_4_ (Scheme 13, reaction a).^120−122^ The preparation of enantioenriched α-allenol **60** from allenoic acid **59** required an esterification followed by reduction with DIBAL-H (Scheme 13, reaction b).^123^ The above processes occur with efficient chirality transfer, which shows the high synthetic potential of this methodology in asymmetry synthesis. The DIBAL-H-promoted reduction of racemic α-allenoates into α-allenols can be conveniently achieved,^124^ while the LiAlH_4_-assisted reduction of enantioenriched α-allenoates **61** (Scheme 13, reaction c^125^ and the double 1,2-addition of allyl magnesium chloride to axially chiral α-allenoate **63** (Scheme 13, reaction d)^126^ generated with retained chirality the corresponding optically active α-allenols **62** and **64**, respectively.

##### 2.2.1.2 Allenols Showing Central Chirality

**Scheme 14:**
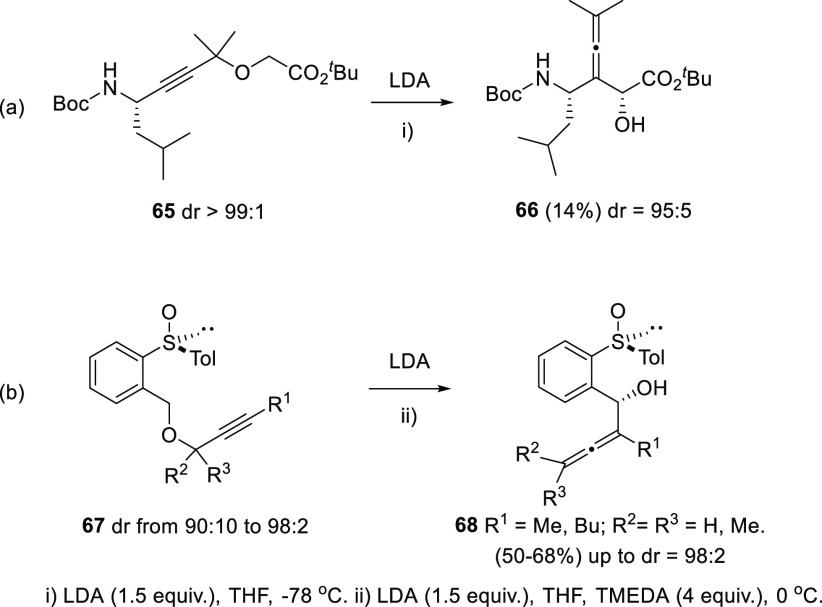
Asymmetric Wittig Rearrangement in α-Allenol Synthesis

[2,3]-Wittig rearrangement of propargylic ethers provides the α-allenol skeleton in one single synthetic step. A first approach to the asymmetric version of the Wittig rearrangement was applied to the synthesis of a pharmacologically attractive α-hydroxy γ-amino acid **66** bearing an allene unit, despite in poor yield (Scheme 14, reaction a).^127^ More recently, a Wittig-based methodology was also employed for the synthesis of a family of substituted asymmetric α-allenols **68**. In this case, a remote chiral sulfoxide in the starting propargylic ether **67** was responsible of the stereochemistry, behaving as a chiral inductor rather than a chirality transfer agent (Scheme 14, reaction b).^128^ In both cases, good diastereoselectivities were achieved, although no axial chirality is described, and the lack of a wider scope in the methodology leaves much work yet to be explored in this field.

**Scheme 15:**
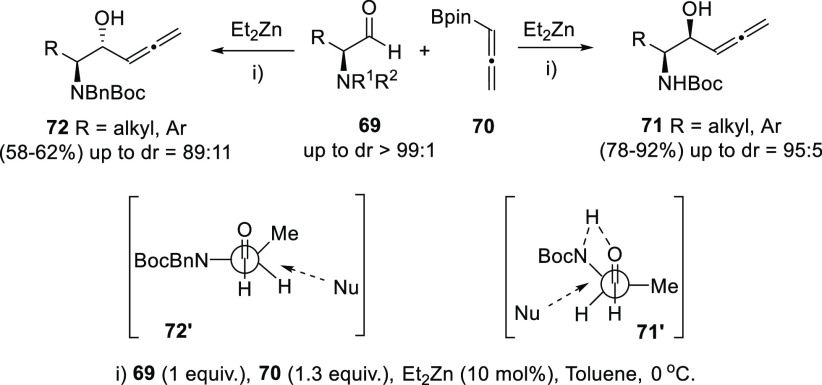
Asymmetric Allenyl Boronate Addition to Aldehydes

Other examples dealing only with central chirality in the newly formed hydroxyl carbon use the asymmetric version of the allenyl boronate addition onto aldehydes. Zn catalysis provides complete regioselectivity toward the allenylation *versus* propargylation processes, while chiral α-amino aldehydes **69** are responsible of the stereoselectivity observed as chiral inductors. Diastereoselectivity can be tuned by simply modifying the aldehyde substitution. NHBoc-substituted aldehydes led to *syn* amino allenols **71** though a Cram-chelation model **71′**, while NBnBoc-substituted aldehydes yielded *anti* isomers **72** through a Felkin-Ahn addition model **72′** (Scheme 15).^129^

**Scheme 16:**
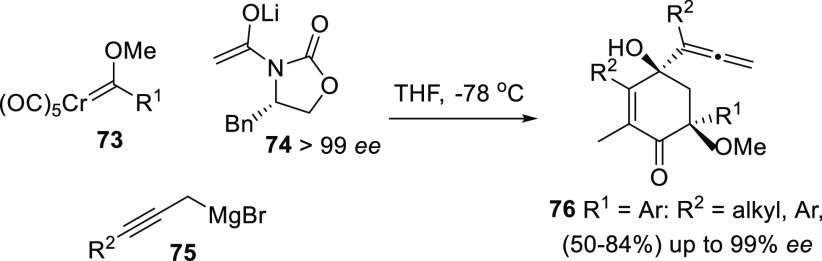
Multicomponent Asymmetric Reaction for the Synthesis of Allenyl Cyclohexenones

More specific transformations include the asymmetric propargylboration of aldehydes using 10-trimethylsilyl-9-borabicyclo[3.3.2]decanes,^130^ or Barluenga’s multicomponent reaction of chromium carbenes **73**, oxazolidine-2-ones lithium enolates **74**, and Grignard reagents **75** to yield highly substituted cyclohexenones **76** bearing allenolic units.^131^ Central-to-central chirality transfer is reported, using optically pure oxazolidines as chiral inductors, and yielding allenols **76** showing up to 99% *ee* (Scheme 16).

#### 2.2.2 Kinetic Resolution of Racemic Allenols

Because of the more effective and economic chirality transfer approaches based in enantiopure catalysts, the past decade has experienced a decay in the number of contributions dealing with kinetic resolution strategies. Nevertheless, pioneer research groups in this field such as Bäckvall’s,^132−135^ have continued their interest in kinetic resolution strategies proposing new alternatives and more efficient procedures.

**Scheme 17:**
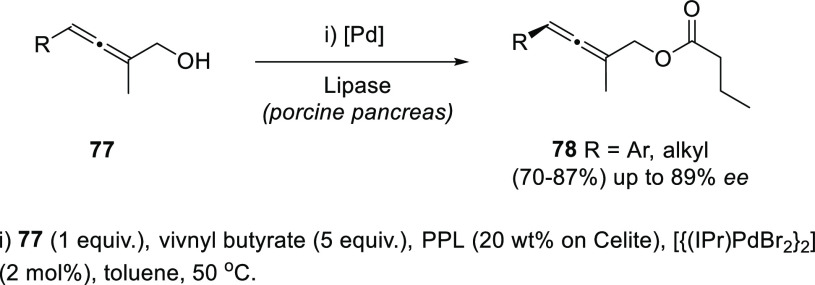
DKR of α-Allenols Using Palladium Catalysis

Dynamic kinetic resolution (DKR) by means of thermal or chemical induced isomerization has been extensively used to overcome the limited yield disadvantage inherent to KR. Axially chiral trisubstituted α-allenols **78** were obtained through esterification of **77** in the presence of lipase from porcine pancreas and vinyl butyrate. DKR was achieved by using palladium catalysis, inducing the allene **77** isomerization through the corresponding π-allyl palladium complex. The reported hybrid chemo-enzymatic methodology led to improved yields up to 83%, and good enantioselectivities (Scheme 17).^136^

**Scheme 18:**
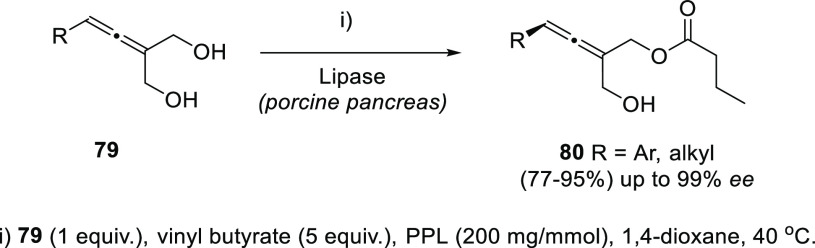
Desymmetrization of Allendiols

One different conceptual approach to get access to chiral allenols with high yields employs prochiral starting materials in desymmetrization processes. Thus, allene diols **79** react selectively with vinyl butyrate in the presence of Lipase from porcine pancreas as sole catalyst to yield optically pure monoesters **80**. Yields were good to excellent for allenes bearing aromatic susbtituents, and expectedly lower for aliphatic systems, though practical high enantioselectivities up to 99% were reported (Scheme 18).^137^

**Scheme 19:**
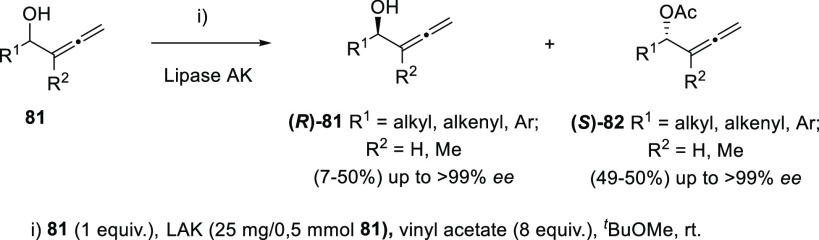
Enzymatic KR of Centrally Chiral α-Allenols

The group of Ma reported in 2002 the use of Novozym-435 as a convenient biocatalyst for the kinetic resolution of racemic α-allenols, giving rise to enantioenriched (*S*)-(−)-α-allenols and (*R*)-(+)-α-allenyl acetates in an efficient way.^138^ Hong and co-workers have contributed to the asymmetric synthesis of allenols with central chirality by developing different KR methodologies. In this regard, both enzymatic and chemical alternatives have been studied. Acetylation of α-allenols **81** in the presence of the appropriate lipase allowed the preparation of optically pure compounds (*R*)-**81** and (*S*)-**82** with yields up to 50% and enantiomeric excesses above 99%. After an enzyme screening, lipase AK (*Pseudomonas fluorescens*) was identified as the best candidate to achieve this transformation. The methodology was expanded to many differently substituted terminal allenols, including alkyl-, alkenyl-, and aryl-decorated structures (Scheme 19).^139^

**Scheme 20:**
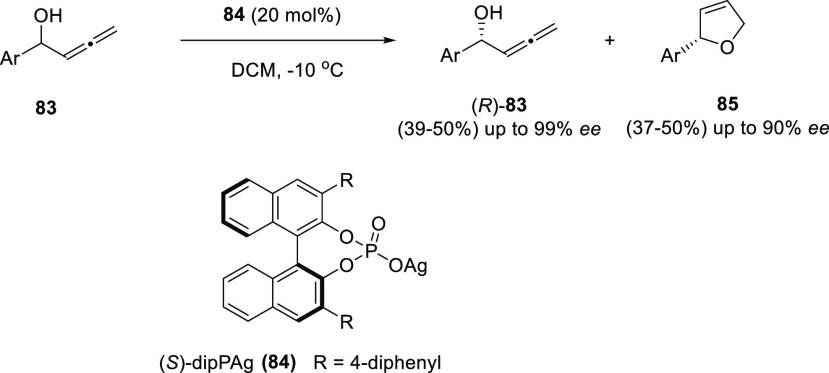
Chemical Kinetic Resolution of Aryl-Substituted Terminal Allenols

Taking advantage of the transition metals ability to catalyze allenol cycloisomerizations, which will be further discussed in the next chapter, a chemical KR of allenols was envisioned. The chiral silver phosphate **84** allowed the selective oxycyclization of the (*S*)-enantiomers from the racemic mixture of allenols **83**. Thus, enantioenriched dihydrofurans **85** were obtained, while unreacted (*R*)-allenols **83** were recovered. Both species were easily separable after column chromatography, providing aryl-substituted terminal allenols with yields up to 50% and up to 99% *ee* (Scheme 20).^140,141^

#### 2.2.3 Asymmetric Synthesis Using Enantiopure Catalysts

The use of enantiopure catalysts represents a strategy of increasing interest in asymmetric synthesis. The possibility to avoid the preparation of optically pure starting materials in large scale, along with the catalyst recycling facilitates more economic synthetic routes. Regarding the synthesis of enantioenriched allenols, both enantiopure ligands in metal catalysis and enantiopure organocatalysts are described.

##### 2.2.3.1 Enantiopure Ligands in Metal Catalysis: Axial and Axial-Central Chirality

**Scheme 21:**
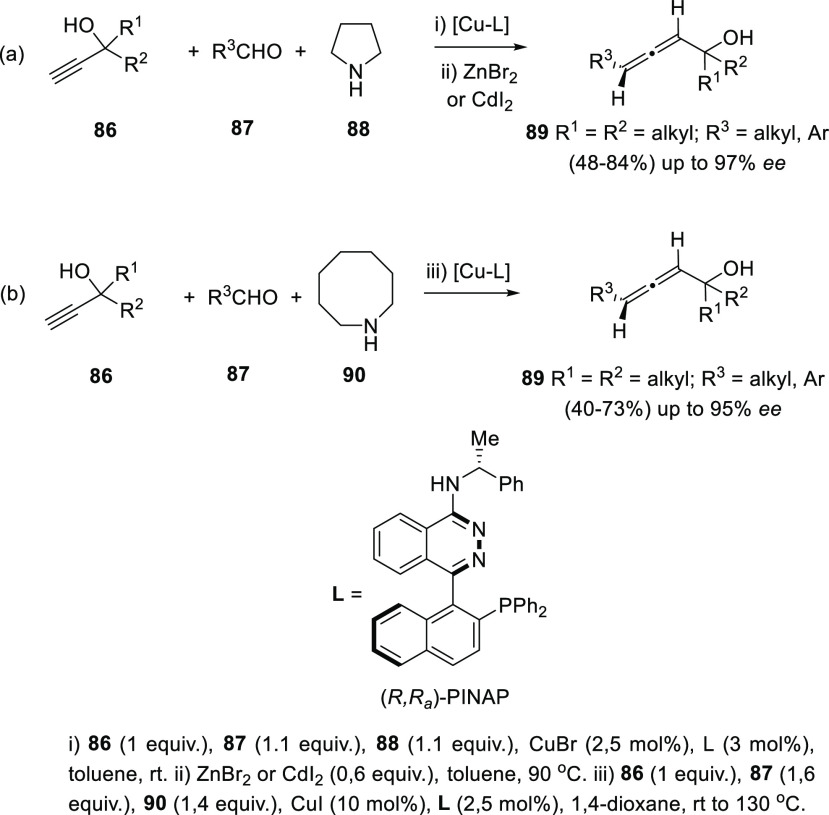
Asymmetric Allenation of Terminal Alkynes Using Chiral Ligands

Ma and co-workers have studied the asymmetric allenation of terminal alkynes using enantiopure ligands as chirality transfer agents in the synthesis of enantioenriched α-allenols. Readily available propargylic alcohols **86** were first submitted to Cu catalysis using pyrrolidine (**88**) as amine and (*R,R*_*a*_)-PINAP as ligand. To achieve practical reaction conversions, cocatalysts ZnBr_2_ or CdI_2_ were needed, describing a one pot/two steps and a one pot/one step procedures respectively (Scheme 21, reaction a).^142,143^ Further investigations on this transformation revealed that increasing the amine ring size (**90**) led to high enantioselectivities under CuI as sole catalytic species in a more efficient and economic manner (Scheme 21, reaction b). Also, reversal enantioselectivity was easily achieved by using enantiomer (*R*,*S*_*a*_)-PINAP as chiral ligand.^144^ Good axial enantioselectivities were reported through a versatile and practical methodology.

**Scheme 22:**
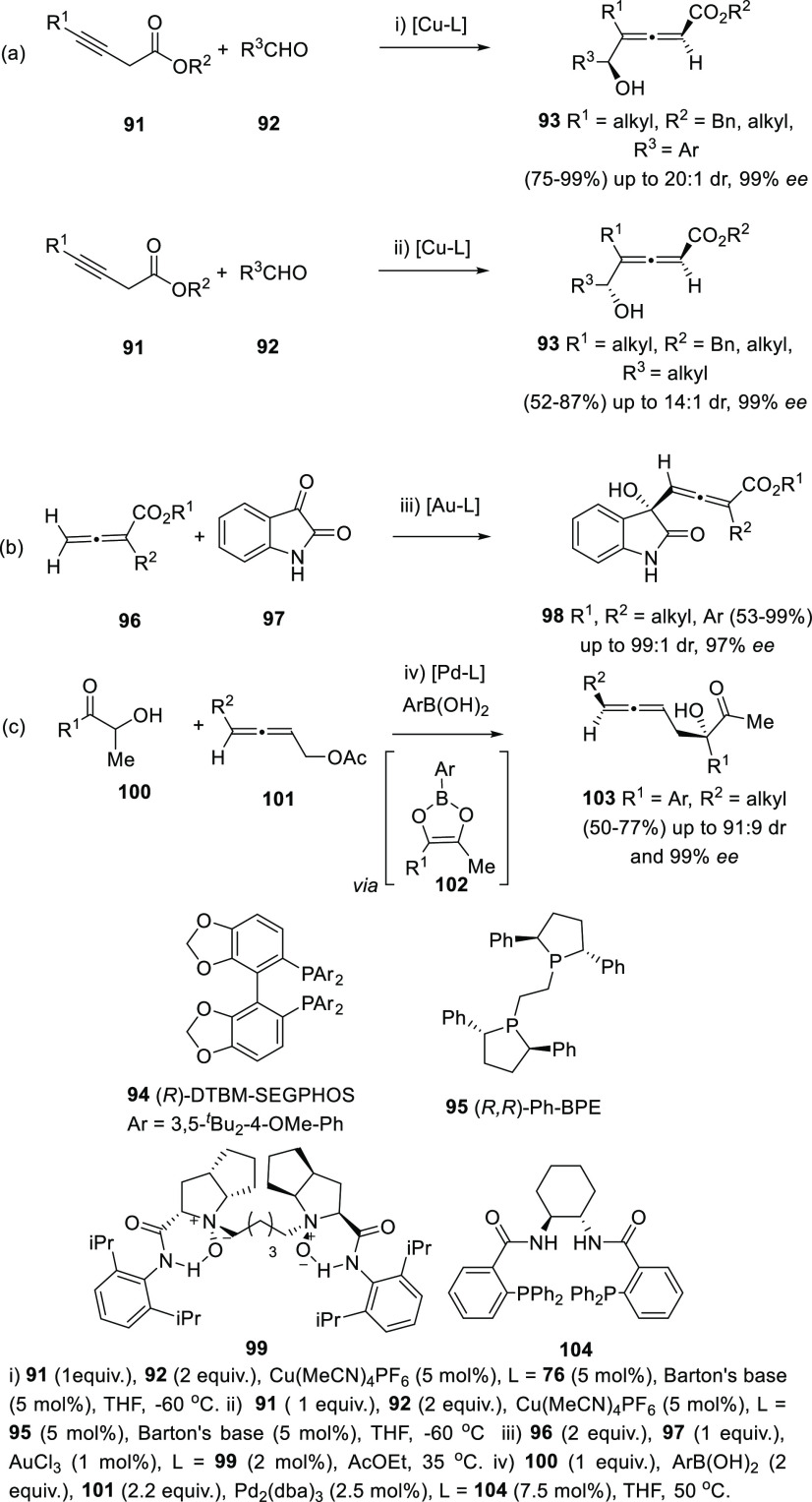
Asymmetric Aldol-type Synthesis of Substituted α- and β-Allenols

Aldol-type additions of both propargylic and allenyl substrates onto carbonyls also find a stereoselective variant based on the use of chiral ligands in transition metal catalysis for the synthesis of α-allenols. Alkynylogous aldol reaction from propargylic carboxylates **91** catalyzed by copper salts and (*R*)-DTBM-SEGPHOS (**94**) as chiral ligand was found to be very effective for the synthesis of 2,3-allenols **93** from aromatic aldehydes (Scheme 22, reaction a). On the other hand, aliphatic aldehydes showed better stereoselectivities in the presence of (*R*,*R*)-Ph-BPE (**95**) as chiral ligand, which provided opposite central enantioselectivity. In both cases, high diastereo- and enantioselectivities were obtained (Scheme 22, reaction a).^145,146^ Au (III) salts have been reported to promote the aldol-type addition of allenic esters **96** onto isatin **97**. In this case, a chiral *N*,*N*-dioxide **99** was used as chirality transfer agent, providing tri- and tetra-substituted allenols **98** in good to excellent yields and good enantioselectivities (Scheme 22, reaction b).^147^ In a different approach, condensation of boronic acids with α-hydroxycarbonyls **100** formed 1,3-dioxaboroles **102**, which can be used as electrophiles in asymmetric allenylation reactions for the synthesis of β-allenols. Thus, racemic allenes **101** were transformed into enantioenriched allenols **103** using palladium catalysis and enantiopure phosphine ligands (Scheme 22, reaction c).^148^

**Scheme 23:**
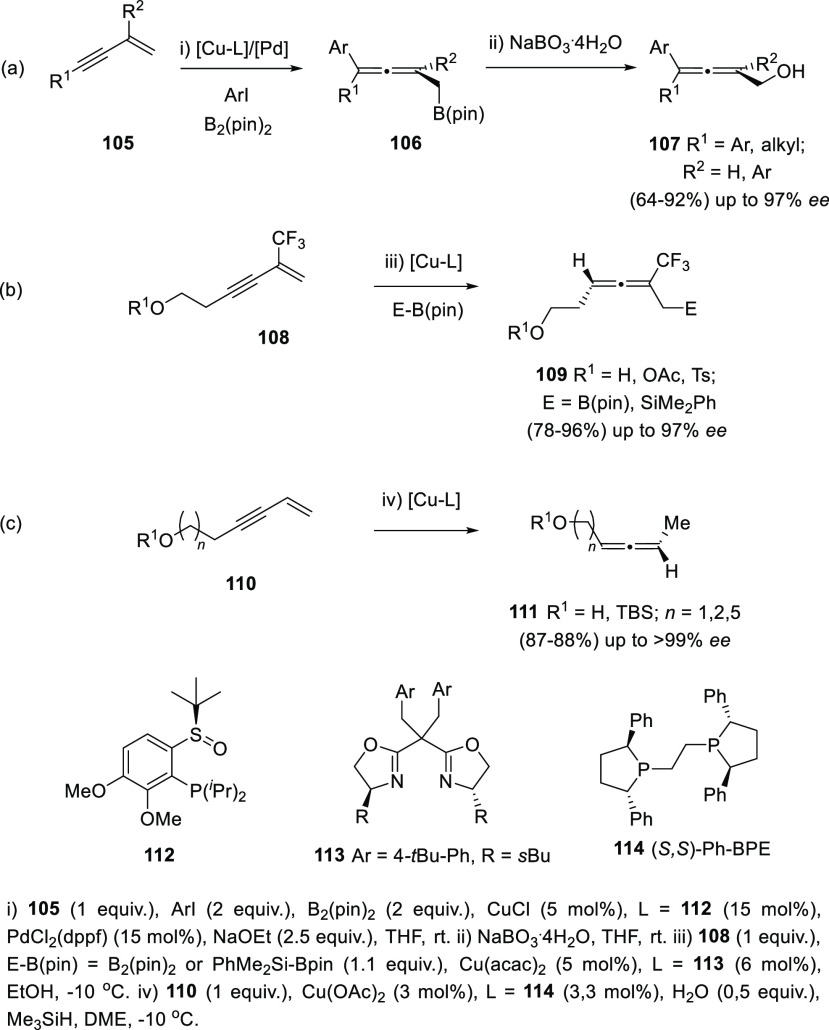
Axially Chiral Allenols from Enynes

1,3-Enynes have also been employed as common starting materials for the asymmetric synthesis of allenols exhibiting diverse allene-hydroxyl connectivity. Challenging tri- and tetra-substituted allenols **107** showing axial chirality are accessible through a cooperative Cu–Pd arylboration of enynes **105**. Treatment of boron intermediates **106** with NaBO_3_ eventually yielded the expected α-allenols **107**. The appropriate use of both metal catalyst and the noncommercial chiral sulfoxide **112** as ligand is reported to be responsible of the high enantioselectivity observed, avoiding racemization of allenyl copper intermediates (Scheme 23, reaction a).^149^ In a different contribution, related 2-trifluoromethyl enynes **108** decorated with a hydroxyl group, provided the allenol skeleton through a similar Cu-catalyzed 1,4-protoborylation or 1,4-protosylilation. In this case, new designed chiral bisoxazoline ligands **113** showed the best results yielding up to 97% *ee* (Scheme 23, reaction b).^150^ Alternatively, copper hydride semireduction of enynes **110** provided axially chiral disubstituted allenols **111**. Mild reaction conditions and the use of water as proton source allowed a wide functional group compatibility. Commercially available 1,2-bis((2*S*,5*S*)-2,5-diphenyl-phospholano)ethane [(*S*,*S*)-Ph-BPE] (**114**) showed enantiomeric excesses above 99% (Scheme 23, reaction c).^151^

##### 2.2.3.2 Enantiopure Ligands in Metal Catalysis: Central Chirality

**Scheme 24:**
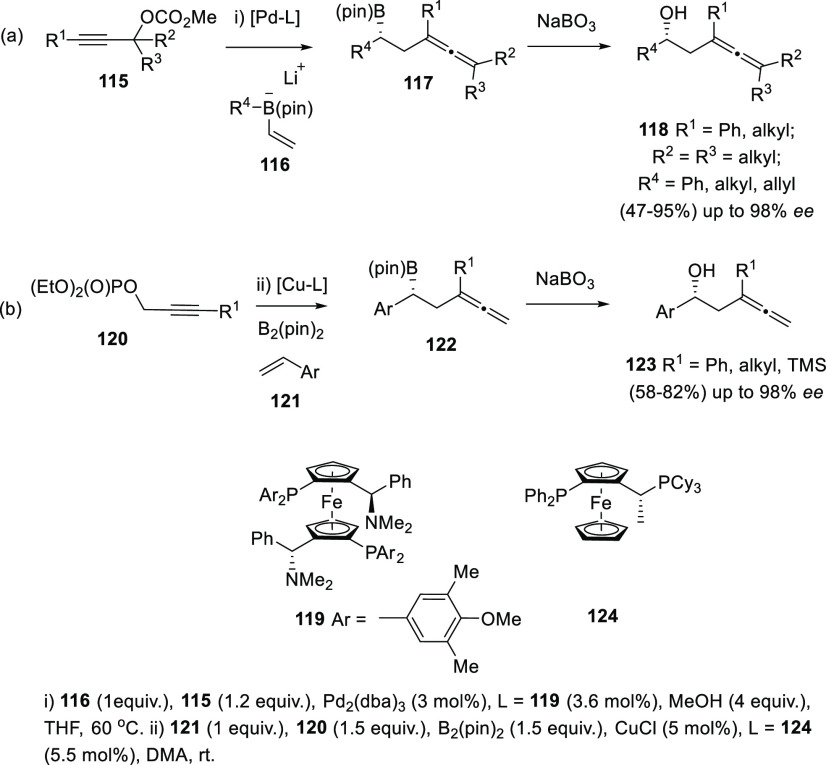
Cross-Coupling Reactions of Borane Complexes and Propargylic Compounds

Besides the above-mentioned coupling reaction with enynes, organoboron reagents have shown great versatility toward allenol preparation, both starting from diverse substrates and through different transition metal-catalyzed reaction mechanisms. Cross-coupling reaction between propargylic carbonates **115** and boronate complexes **116** under Pd catalysis afforded β-boryl allenes **117** as reaction intermediates. Again, boron functionalization was effectively used as hydroxyl precursors by treatment with NaBO_3_. (*S*,*S*)-MandyPhos ligand (**119**) was employed to induce central chirality, providing β-allenols **118** with good yields and enantioselectivities up to 98% (Scheme 24, reaction a).^152^ A related approach reacted vinyl arenes **121** and bis(pinacolato)diboron B_2_(pin)_2_ with propargylic phosphates **120** under Cu catalysis and (*R,S*)-Josiphos (**124**) as chirality transfer agent. Following a similar strategy, the hydroxyl group was obtained after treatment of the corresponding β-boryl allenes **122** with NaBO_3_ (Scheme 24, reaction b).^153^

**Scheme 25:**
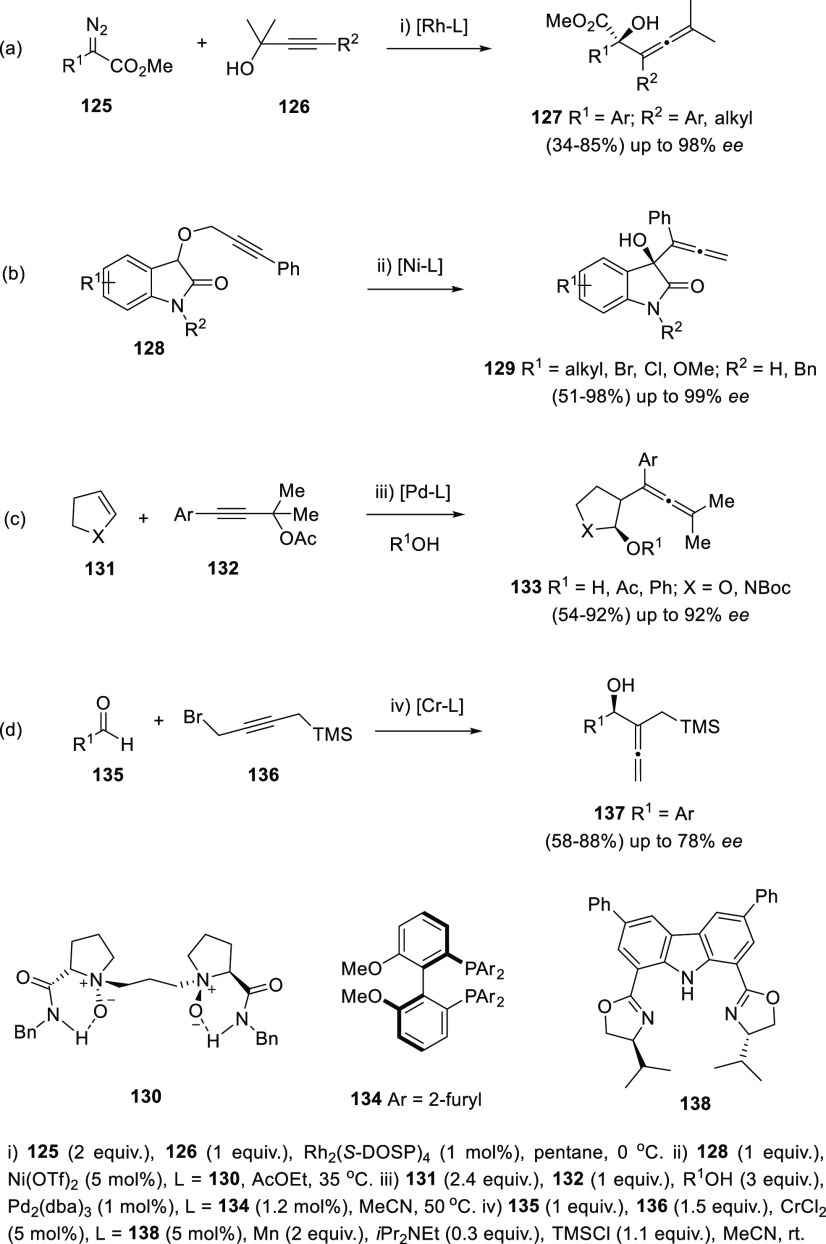
Allenol Asymmetric Synthesis from Propargylic Derivatives through Diverse Procedures

More particular transformations based on enantiopure ligands include reaction between diazoesters **125** and propargylic compounds **126** through a tandem ylide formation/[2,3]-sigmatropic rearrangement (Scheme 25, reaction a),^154^ [2,3]-Wittig rearrangement of propargylic isatins **128** (Scheme 25, reaction b),^155^ Wacker-type oxyallenylation of cyclic alkenes **131** (Scheme 25, reaction c),^156^ or a Cr-catalyzed addition of propargyl bromides **136** onto aldehydes (Scheme 25, reaction d).^157^

##### 2.2.3.3 Enantiopure Organocatalysts

**Scheme 26:**
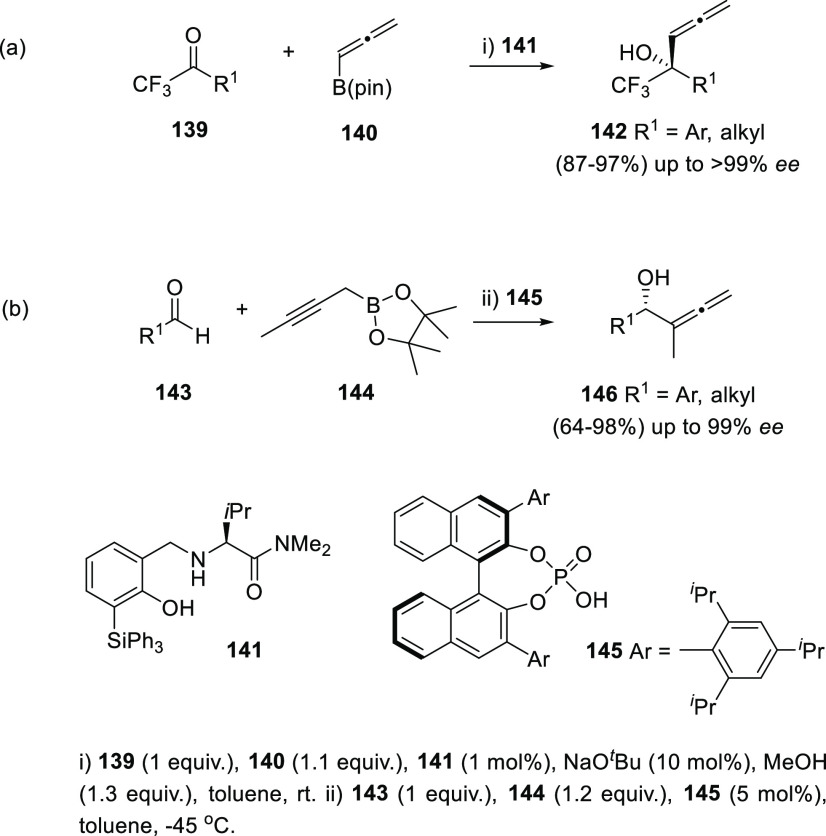
Organocatalyzed Addition of Allenyl and Propargyl Boronic Complexes to Carbonyls

Enantiopure organocatalysts have been scarcely reported for the synthesis of allenols. Nevertheless, during the last years, different research groups have started to apply this methodology to the asymmetric addition of propargyl and allenyl compounds to carbonyls. This transformation unravelling intriguing catalytic strategies allows the synthesis of both axially and centrally chiral allenols with good yields. Hoveyda’s research group has described a general methodology for the asymmetric nucleophilic addition to carbonyls controlled by fluorine–ammonium electrostatic interactions. The organocatalyzed procedure was applied to the addition of allenyl boronic complexes **140** to trifluoromethyl ketones **139**, yielding α-allenols **142**. An enantiopure organocatalyst **141** showing the appropriate electronic features delivered allenols **142** in excellent yields and enantioselectivities above 99% (Scheme 26, reaction a).^158^ Chen and co-workers have also studied a related organoboron addition onto carbonyls for the asymmetric approach to the allenol skeleton. In this case, propargylic boronates **144** were added to aldehydes in the presence of a chiral phosphoric acid **145**, providing α-allenols **146** with good to excellent yields and showing central chirality with up to 99% *ee* (Scheme 26, reaction b).^159^

**Scheme 27:**
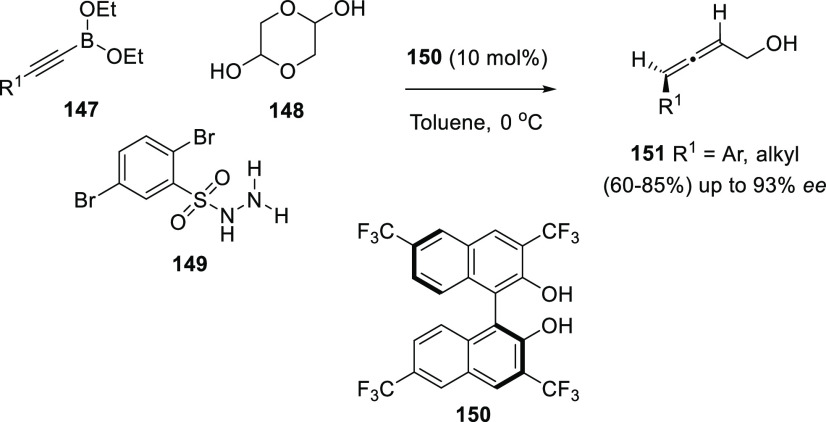
Organocatalyzed Asymmetric Traceless-Petasis for the Synthesis of Allenols

Following with the extensive use of unsaturated organoboron compounds, and continuing with the applications of Petasis-type reactions, Thomson and co-workers have developed a chiral organocatalyzed version of the multicomponent reaction between propargyl boronates **147**, protected aldehyde **148**, and sulfonyl hydrazine **149**. Enantiopure biphenol **150** gave access to enantioenriched allenols **151** displaying axial chirality, with moderate to good yields and up to 99% *ee* (Scheme 27).^160^

**Scheme 28:**
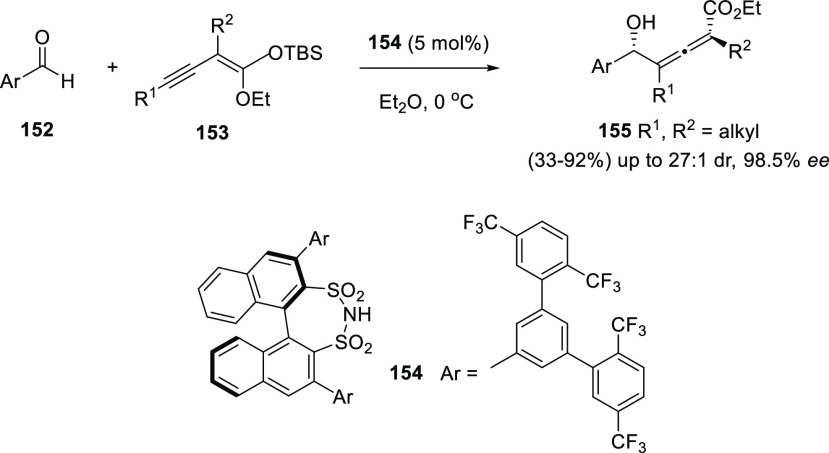
Organocatalyzed Alkynylogous Mukaiyama Aldol Synthesis of Allenols

Organocatalyzed alkynylogous Mukaiyama aldol reaction also constitutes a feasible methodology for the asymmetric allenol preparation. List and collaborators have recently reported an enyne addition onto aldehydes catalyzed by a newly designed chiral disulfonimide **154**. Challenging tetrasubstituted allenols **155** were prepared, exhibiting both axial and central chirality. The scope of the transformation includes aromatic aldehydes **152** and differently substituted alkyl enynes **153**. Mild reaction conditions are reported, leading to moderate or excellent yields, diasteroselectivities up to 27:1, and enantiomeric excesses up to 98.5% (Scheme 28).^161^

## 3 Synthetic Utility

### 3.1 Allenols as π-Activated Alcohols

**Scheme 29:**
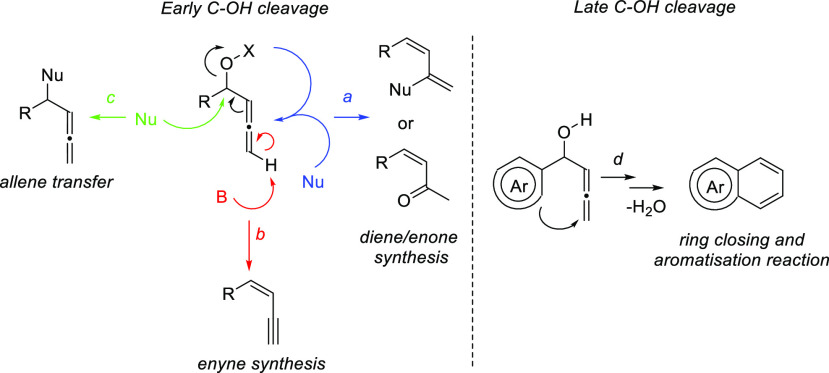
General Reaction Mechanisms for C–OH Cleavage in α-Allenols

Hydroxyl units are traditionally considered bad leaving groups in organic chemistry, unless previous OH-activation has been made. π-*A*ctivated alcohols are a special class of hydroxylic compounds in which the positive charge at the α-carbon is stabilized by the presence of conjugated π-orbitals. Taking advantage of this particular reactivity, π-activated α-allenols have been reported to undergo a wide number of transformations where the C**–**O bond cleaves at one certain point of the reaction mechanism. In this context, reactivity of allenols may be divided in two main groups: (i) those where the OH leaves the molecule at the first steps of the mechanism, leading normally to the diene, enone or enyne skeletons, and (ii) those where the OH loss occurs at the final stages of the process, which are normally found in tandem reactions for the synthesis of aromatic rings and alkaloids. Thus, either when a 1,3-migration reaction takes place or an external nucleophile attacks the central allenic carbon promoting the extrusion of the previously activated alcohol in a S_N_2′-type reaction, diene/enone skeletons may be formed (Scheme 29, path a). On the other hand, if a base abstracts a terminal allenic proton, rearrangement and activated alcohol elimination can take place to yield the enyne motif (Scheme 29, path b). In addition, when the allenic carbocation resulting from C**–**O bond dissociation is stable enough, an allene transfer process may happen by nucleophilic trapping, retaining the allene moiety (Scheme 29, path c). Finally, C**–**OH cleavage can take place through a further dehydratation step after carbo- or heterocyclization processes, leading normally to aromatic or heteroaromatic compounds (Scheme 29, path d).

#### 3.1.1 OH as a Leaving Group in the First Stage of the Reaction

##### 3.1.1.1 Synthesis of Dienes and Enones

Dienes are easily generated from allenols and protected allenols trough 1,3-rearrangement processes. Several methodologies including acid or base promoted isomerizations and metal promoted reactions have been recently reported.^162−185^

**Scheme 30:**
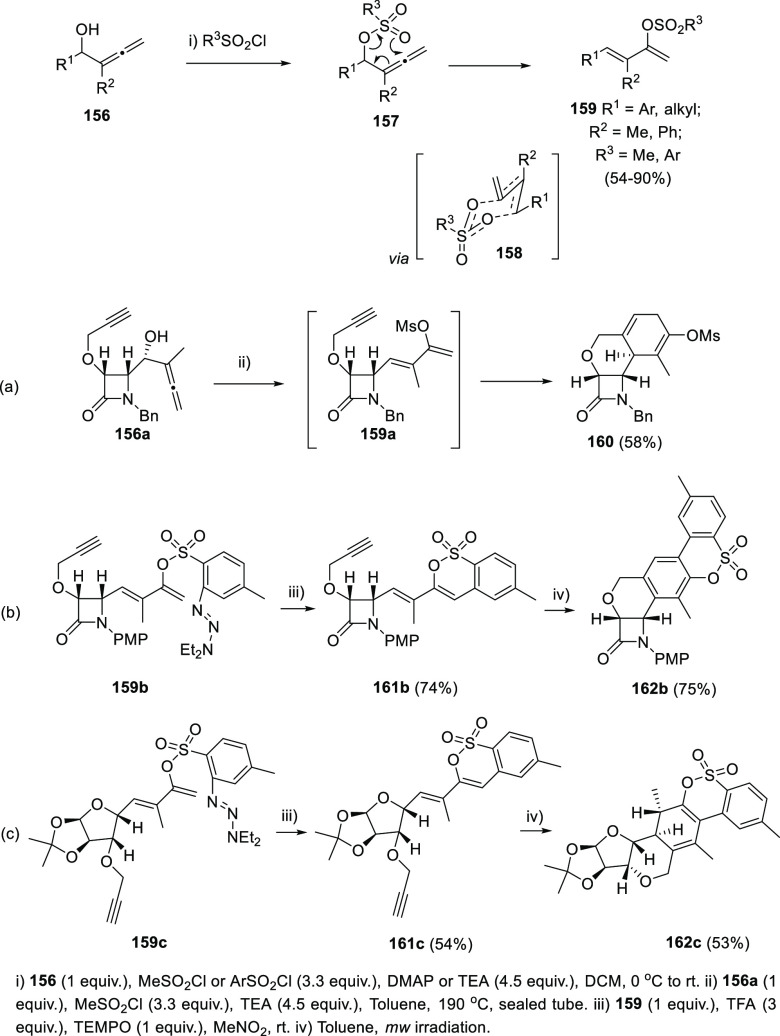
Methyl and Arylsulfonyl Chloride Promoted Synthesis of Dienes and Its Application toward the Synthesis of Polycyclic Structures

Starting from allenols **156** and the appropriate sulfonyl chloride, the Alcaide and Almendros research group described a novel [3,3]-sigmatropic rearrangement of nonisolable α-allenic methanesulfonates and arylsulfonates **157**. The formal OH migration to yield dienes **159** is proposed to proceed through a six membered ring transition state **158** (Scheme 30).^186,187^ DFT calculations supported an aromatic transition state in accordance to a pericyclic reaction mechanism, in view of a negative nucleus independent chemical shift obtained at the ring critical point of the electron density (NICS = −6.5 ppm). Also, a low calculated activation barrier of only 17.7 kcal/mol shows coincidence with the mild experimental reaction conditions needed for the transformation.

The methodology was extended to a wide number of substituted allenols **156** and applied to the preparation of different fused polycyclic structures. In one hand, a tandem [3,3]-sigmatropic rearrangement/Diels–Alder reaction provided optically pure tricyclic β-lactams such as **160** (Scheme 30, reaction a).^186^ On the other hand, related [3,3]-sigmatropic transposition of arylsulfonyl allenes produced dienes **159b** or **159c**, which are employed to the synthesis of enantiopure polycyclic sultones such as **162b** or **162c** trough a two-step sequence (Scheme 30, reactions b and c).^187^

**Scheme 31:**
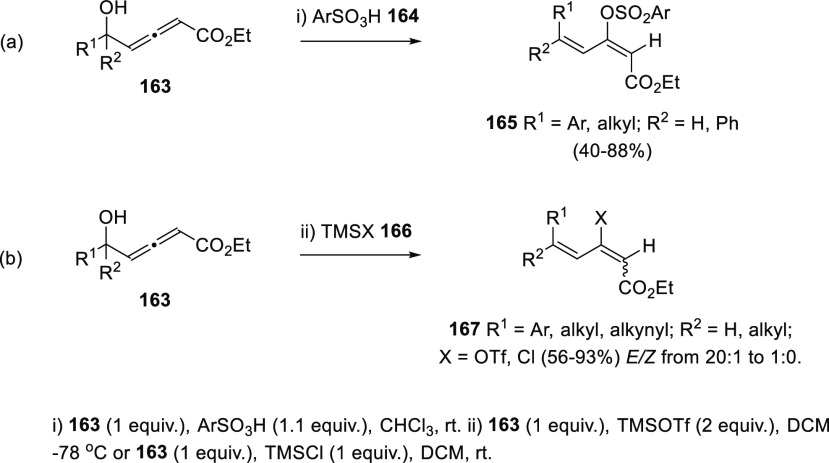
Scope of Metal-Free [3,3]-Sigmatropic Rearrangement of α-Allenols

Wang’s research group has presented an alternative procedure for the [3,3]-sigmatropic allenol rearrangement using sulfonic acids **164** instead of sulfonyl chlorides. The methodology was applied to di- and trisubstituted allenols **163** (Scheme 31, reaction a).^188^ Related work from Lee and collaborators provided *E*-dienes **167** with good yields and good to excellent stereoselectivities using trimethylsilyl triflate or trimethylsilyl chlorides **166**. In this case, triflate- and chlorine-decorated dienes **167** were respectively prepared, expanding the scope of diene functionalization. DFT calculations also pointed to a similar six membered aromatic transition state, based on hydrogen bonding. Nevertheless, the change of 1,3-migration reagent from sulfur- to TMS-derivatives seemed to induce a slight loss of stereoselectivity, leading to *E*/*Z* mixtures in rates depending on the allenol substitution (Scheme 31, reaction b).^189^

**Scheme 32:**
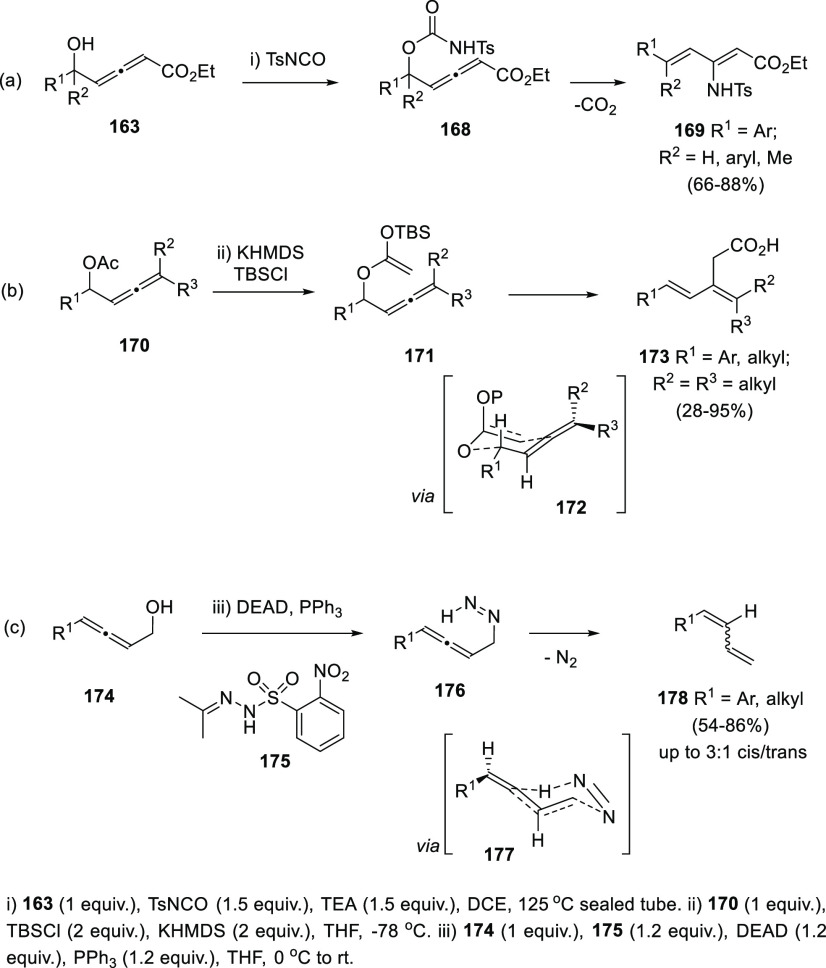
Metal-Free 1,3-Migration Strategies for the Synthesis of Dienes from α-Allenols

Different 1,3-migration strategies involving a previous reaction of the OH unit in α-allenols with coupling or protecting reagents have appeared. Thus, reaction of allenols **163** with TsNCO yielded the corresponding allenic *N*-tosylcarbamates **168**. Taking advantage of the thermal instability of these *N*-tosylcarbamates, a decarboxylative aza-Michael addition/elimination sequence generating dienes **169** has been induced by heating at 125 °C in the presence of a basic catalyst (Scheme 32, reaction a). The 4-ethoxycarbonyl substitution on the allene moiety seems to be crucial for the transformation, although both alkyl and aryl substituents at the carbinolic core are well tolerated, and excellent steroselectivities achieved.^190^

Protection of the hydroxyl group as acetate led to acetoxyallenes **170**, which were described to undergo an Ireland-Claisen-type rearrangements in the presence of a base. A [3,3]-sigmatropic reaction mechanism was therefore proposed, proceeding *via* a six membered chair-type transition state **172** (Scheme 32, reaction b). It was also described the use of *N*,*N*-dimethylacetamide dimethylacetal as protective reagent instead of acetic acid, promoting an Eschenmoser-Claisen rearrangement leading to similar results.^191^

Reaction of the alcohol unit in allenols **174** under Mitsunobu conditions using *N*-isopropylidine-*N*′-2-nitrobenzenesulfonyl hydrazine (**175**) as nucleophile, led to allenyl diazenes **176**. Those substrates were envisioned as precursors of 1,3-dienes through a reductive transposition, *via* a retro-ene-type transition state **177**. The above methodology generated unsubstituted dienes **178** in moderate to good yields. Opposite to previously mentioned [3,3]-sigmatropic rearrangements, the reaction course proceeded with a notable lack of stereoselectivity, yielding cis/trans dienes in rates from 3:1 to 1:1 (Scheme 32, reaction c).^192^

**Scheme 33:**
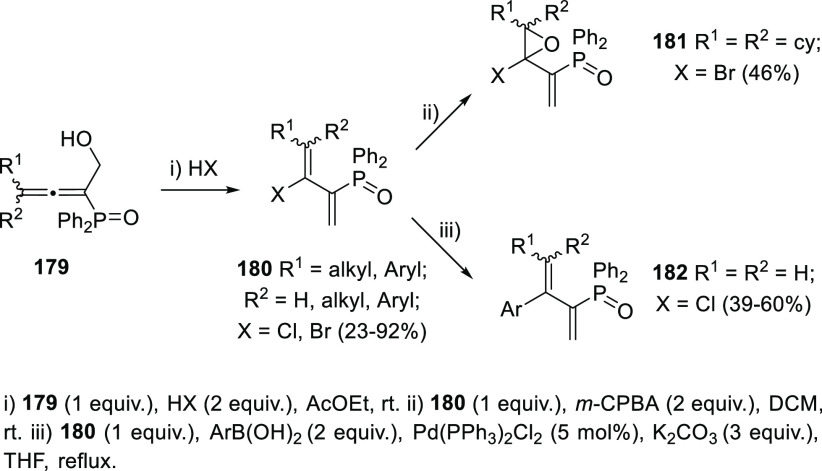
Acid Promoted Synthesis of Halogenated Phosphinoyl Dienes and Synthetic Applications

Preprepared HCl or HBr solutions in ether or ethyl acetate are reported to promote the isomerization of (1-hydroxybuta-2,3-dien-2-yl)diphenylphosphine oxides **179** into chlorinated or brominated phosphinoyl 1,3-butadienes **180**. The methodology is applied to primary alcohols, and no stereocontrol is stated at the C3–C4 double bond. Added value to this acid-promoted metal-free methodology is given by further applicability of halogenated dienes **180** on epoxidation or Suzuki-type reactions, providing tetrasubstituted epoxides **181** and aryl-substituted dienes **182**, respectively (Scheme 33).^193,194^

**Scheme 34:**
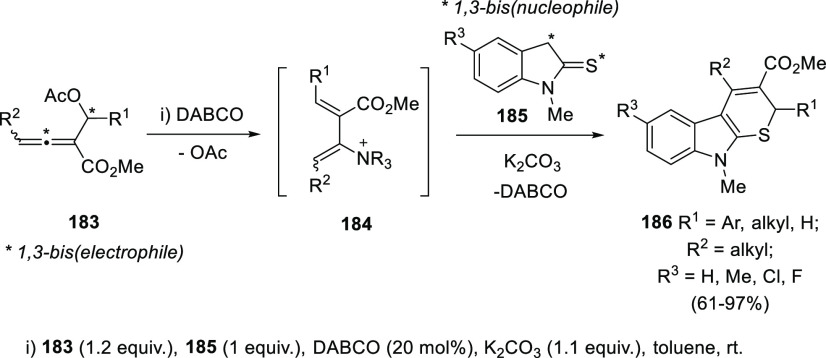
Dihydrothiocarbazole Synthesis by Reaction of Indoline-2-thiones and *In Situ* Generated Dienes

A conceptually different approach for the synthesis of dienes was based on a S_N_2′ reaction in 1-acetoxy-2-allenoates **183**. DABCO addition onto the central allenic carbon induced the generation of 1,3-dienes **184** facilitated by extrusion of the AcO group. Tong and co-workers envisioned intermediate 1,3-diene-2-amonium species **184** as adequate 1,3-bis(electrophiles) for the reaction with 1,3-bis(nucleophiles) in a formal (3 + 3) ring closing reaction. Indoline-2-thiones **185** were selected as ideal candidates, showing 1,3-bisnucleophilic nature in the presence of a weak base. Thus, reaction of 1-acetoxy-2-allenoates **183** and indolines **185** in the presence of DABCO provided dihydrothiocarbazoles **186** through a S_N_2′–S_N_2′ reaction sequence (Scheme 34).^195,196^

**Scheme 35:**
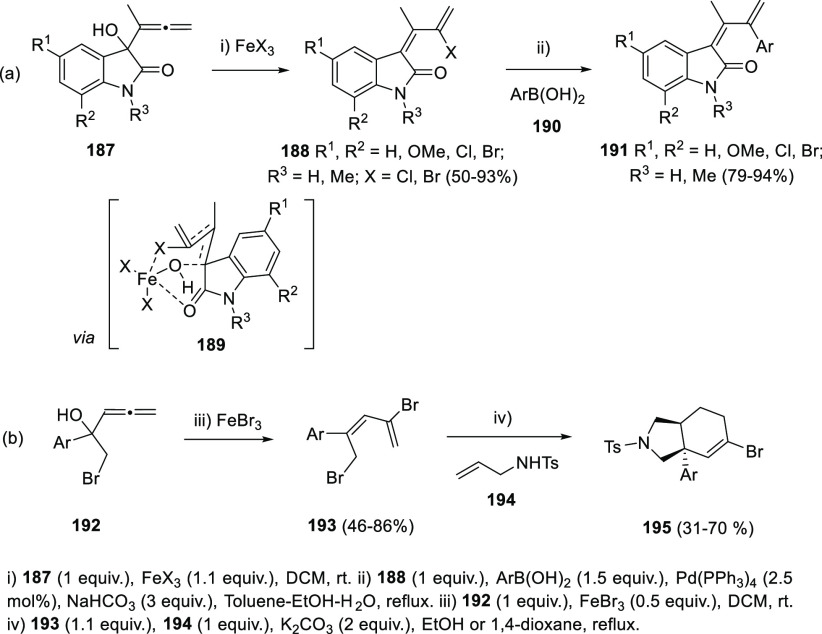
Iron-Promoted Halogenation/Rearrangement of Allenols and Synthetic Applications

Metal species are also reported to promote isomerization of allenols to dienes in both equimolecular and catalytic manner. The cobalt-catalyzed regioselective C8 dienylation of quinoline *N*-oxides with allenylcarbinol carbonates has been reported by Volla and co-workers, while the use of unprotected allenylcarbinols as the dienylating agents resulted in diminished yields.^197^ Alcaide and Almendros research group has described the use of FeBr_3_ or FeCl_3_ for the halogenation/rearrangement of 2-indolinone-tethered allenols **187** yielding 2-halo-1,3-dienes **188** (Scheme 35, reaction a). The transformation tolerated different substitution on the aromatic ring and exhibited complete *Z*-selectivity in every case. The high stereoselectivity observed could be explained considering a pseudopericyclic transition state **189**, rather than a stepwise reaction mechanism. Thus, reaction course could be initiated by coordination of the OH group to the metal salt acting as a Lewis acid. Then, a six-membered chair-type transition state **189** facilitates the cleavage of the hydroxyl with concomitant halogen delivery. Extra coordination of the metal ion with the C=O group displayed in axial position could also support the high stereoselectivity found for this transformation. Further Suzuki-Miyaura coupling reaction from 2-halo-1,3-dienes **188** and aryl boronic acids **190** provided the corresponding aryl-substituted dienes **191**, showing the synthetic applicability of the methodology.^198^

Following a similar idea, Lin and co-workers developed a FeBr_3_-mediated bromination/rearrangement reaction to yield related 2,5-dibromo-4-aryl-1,3-pentadienes **193**. Further one-pot *N*-alkylation/Diels-Alder reaction with tosyl-amines **194** provided products **195** bearing the hexahydro-1-*H*-isoindole skeleton with good yields and high diastereoselectivities (Scheme 35, reaction b).^199^

**Scheme 36:**
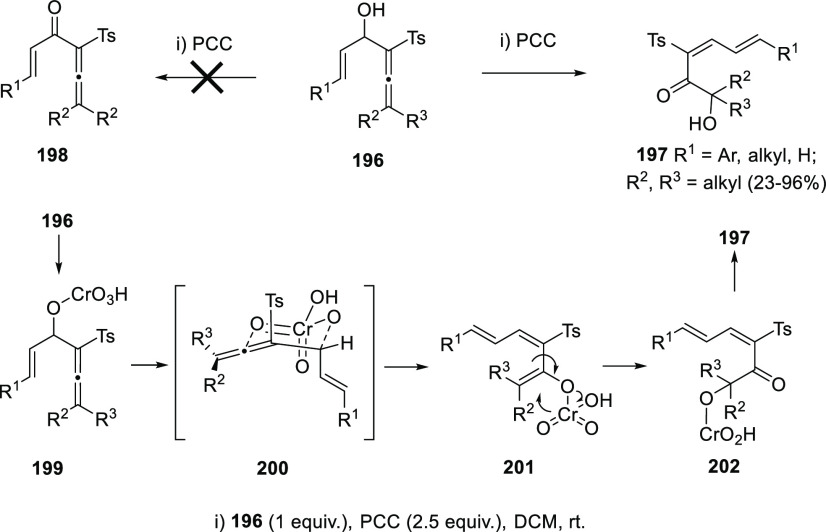
Chromium-Mediated Rearrangement of Allenyl Vinyl Alcohols

The ability of α-allenols to easily isomerize to dienes through 1,3-migration reactions was also illustrated during the attempts to oxidize allenyl vinyl alcohols to the corresponding allenyl vinyl ketones. Harmata and collaborators envisioned the synthesis of ketones **198** as starting materials for Nazarov cycloadditions using PCC as mild oxidant. Surprisingly, Cr-mediated 1,3-migration took place leading to unexpected α′-hydroxydienones **197** (Scheme 36). According to the authors, a mechanistic explanation for this result could start from formation of the chromate ester **199**, followed by 1,3-transposition through the habitual six-membered chair-type transition state **200** to give the chromium enolate **201**. Spontaneous (2,3)-sigmatropic rearrangement could produce the new chromate ester **202**, which could yield the observed dienyl ketones **197** after hydrolysis. The methodology was extended to a wide number of structures with different substituents, exhibiting moderate to excellent yields and excellent stereoselectivity.^78^

**Scheme 37:**
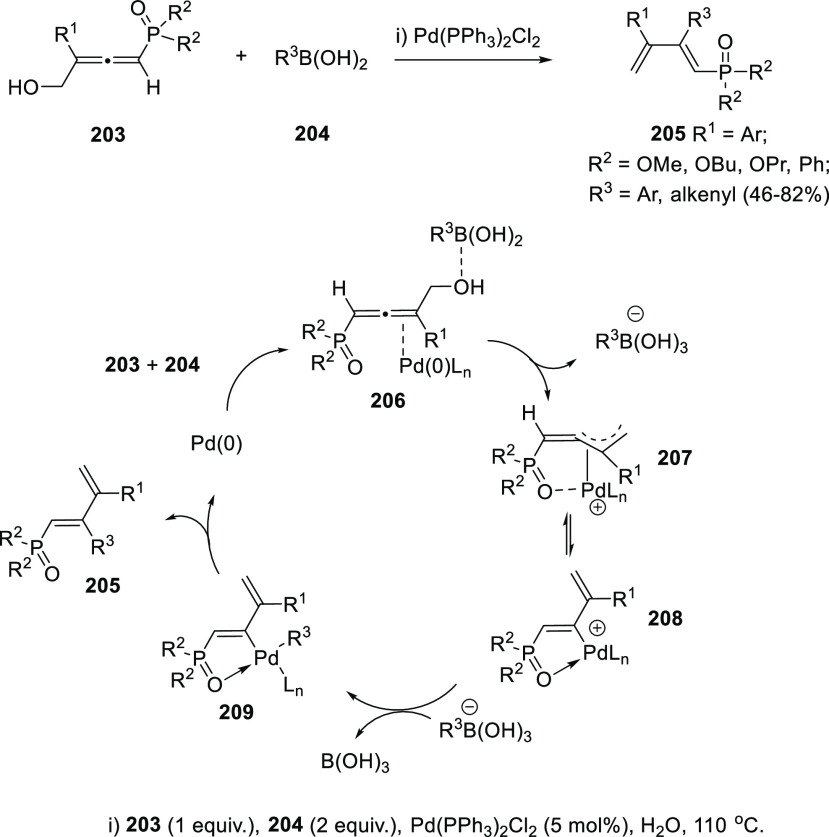
Palladium-Catalyzed Synthesis of 1-Phosphoryl 1,3-butadienes

Metal species in catalytic amounts have also been reported to promote allenol transformations into dienes showing several advantages. As previously mentioned, phosphinoyl allenols were described to react in the presence of acid solutions yielding phosphinoyl dienes despite of low efficiency and lack of stereoselectivity (**179** to yield **180** in Scheme 33). Nevertheless, related 4-phosphoryl-2,3-allenols **203** have been recently found to provide the corresponding 1-phosphoryl 1,3-butadienes **205** in excellent diastereoselectivities using palladium catalysis through a Suzuki-Miyaura cross-coupling reaction with aryl boronic acids **204** (Scheme 37). A plausible reaction mechanism could start with coordination of the metal species to the terminal C–C double bond, and simultaneous activation of the OH groups with the boronic acid in complex **206**. C–O bond cleavage would then take place generating the corresponding π-allyl palladium complex **207**. The high diasteroselectivity resulting from this transformation could be explained by the extra coordination of the metal unit with the P(O) group, leading to the stabilized vinyl palladium complex **208**. Transmetalation and reductive elimination in species **209** would provide observed phosphinoyl dienes **205** and regenerate Pd(0) to the catalytic cycle (Scheme 37).^200^

**Scheme 38:**
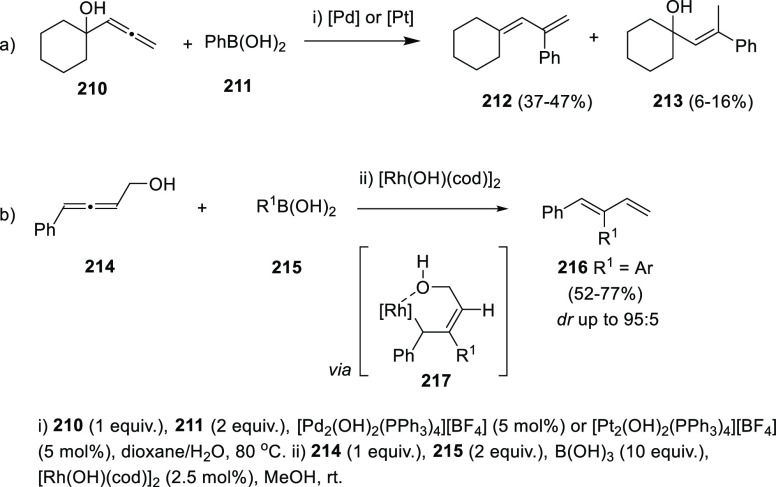
Metal-Catalyzed Synthesis of Dienes in the Presence of Boronic Acids

Palladium and platinum catalysis has also been employed in the transformation of simple allenols and boronic acids. Exploring the addition of arylboronic acids onto a wide variety of allenes, one example of 1,3-diene synthesis is reported when allenol **210** is submitted to Pd or Pt conditions in the presence of boronic acid **211**. Nevertheless, chemoselectivity is not complete, and addition of boronic acids without dehydratation is observed (Scheme 38, reaction a).^201^

A rhodium-catalyzed alternative for this transformation was described using 4-arylbuta-2,3-dien-1-ols (**214**) as starting material and different aryl boronic acids **215**. In this case, carbometalation across the phenyl-substituted allenic bond, followed by δ-elimination of Rh(I)–OH was proposed as mechanistic rationale. Again, metal catalysis provided higher selectivity toward the *Z*-dienes **216** compared with the metal-free analogous transformation. Nevertheless, the lack of an extra coordination site such as the phosphoryl group in allenols **203** resulted in a slight decrease in the *Z*/*E* ratio, observing mixtures of diastereomers (from 89:11 to 95:5) in dienes **216** (Scheme 38, reaction b).^202^

**Scheme 39:**
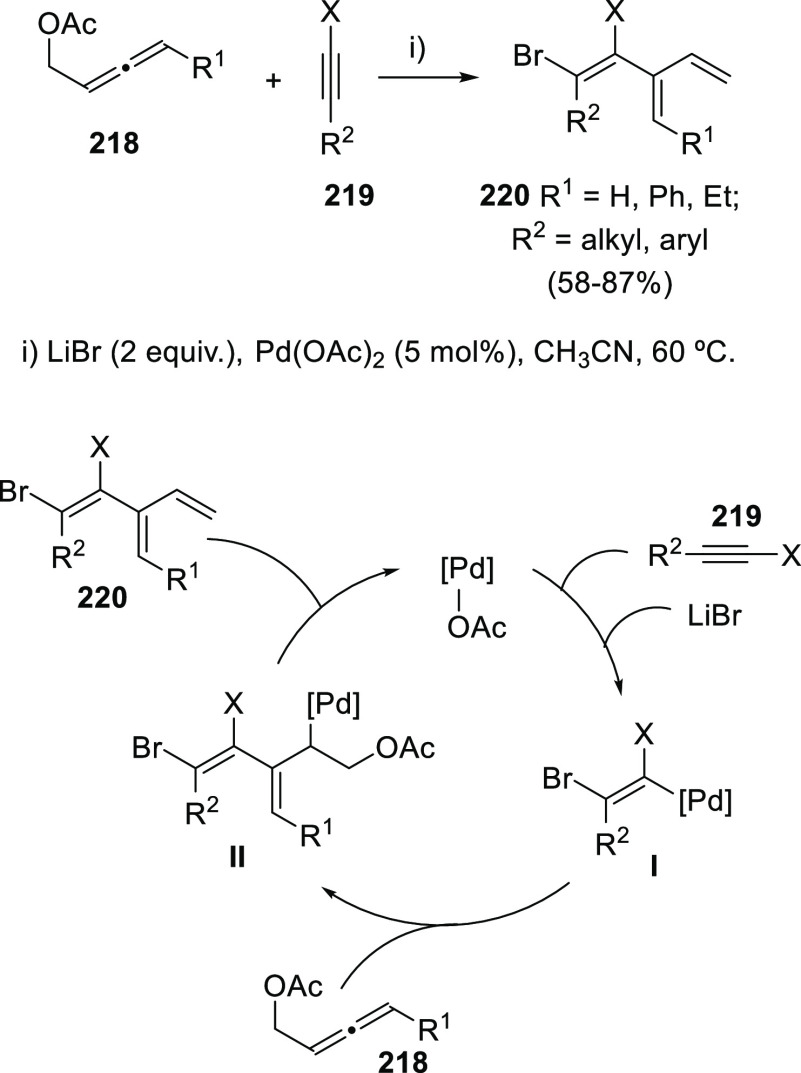
Palladium-Catalyzed Synthesis of Trienes from Allenyl Acetates and Mechanistic Rationale

The palladium-catalyzed preparation of (1*Z*)-1,2-dihalo-3-vinyl-1,3-dienes **220** has been accomplished in a stereoselective manner through the coupling between allenol esters, namely 2,3-butadienyl acetates **218**, and haloalkynes **219** in the presence of lithium bromide (Scheme 39, top). Particularly interesting is the finding that haloalkynes show increase reactivity in comparison with allenes or acetylenes under the halopalladation reaction conditions. A plausible reaction path is depicted in Scheme 39 (bottom). The initial formation of alkenyl-palladium intermediates **I** should occur by *trans*-addition of the halide toward haloalkynes **219**. Next, the carbopalladation reaction with allenol acetates **218** should form allyl-palladium intermediates **II**. β-Heteroatom elimination releases trienes **220** with concomitant regeneration of the catalytic species.^203^

**Scheme 40:**
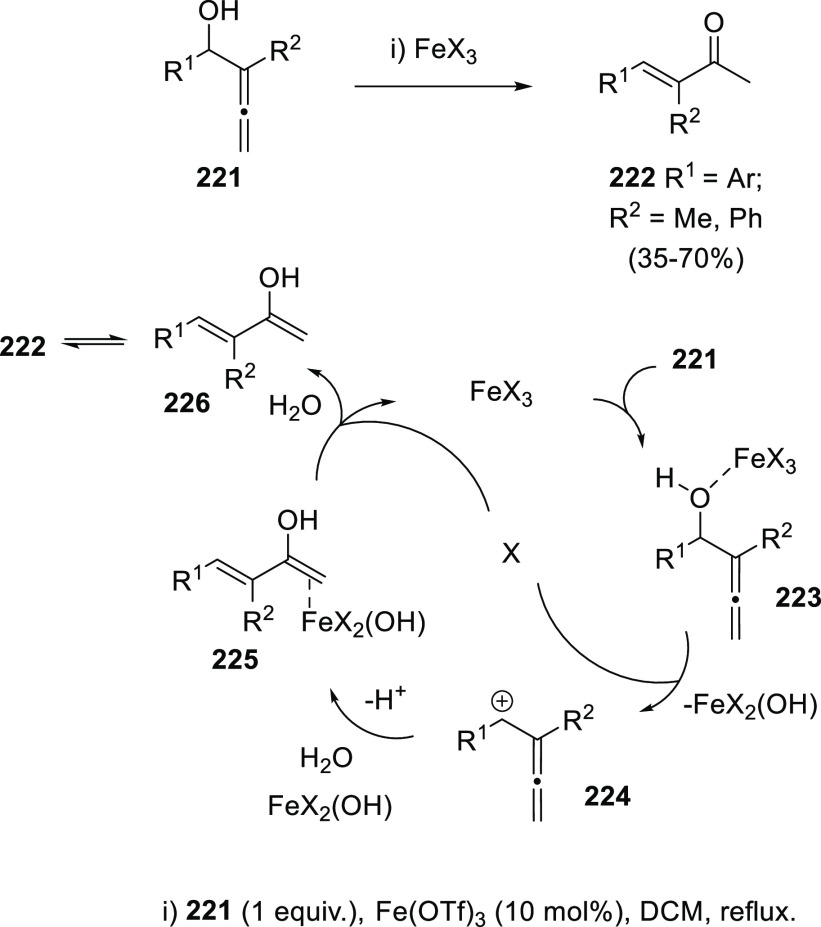
Synthesis of Enones by Iron-Catalyzed Meyer-Schuster Rearrangement and Mechanistic Rationale

A different mechanistic pathway was proposed to explain the results observed from the reaction of different α-allenols **221** in the presence of catalytic amounts of iron triflate or iron trichloride. Opposite to the above-mentioned halogenation/rearrangement reaction of allenols promoted by iron halides in equimolecular manner (compounds **191** and **193** in Scheme 35), catalytic addition of similar metal species yielded the enone skeleton **222** through a Meyer-Schuster-type rearrangement (Scheme 40). The methodology showed best results for aryl- and heteroaryl-substituted allenols, and Fe(OTf)_3_ as metal catalyst, avoiding halogenated byproducts as observed in the presence of FeCl_3_, or decomposition products when acid catalysis was employed. The high *E*-selectivity observed in final enones **222** was found to be independent of the geometry in the starting materials, pointing to a stepwise reaction mechanism. Thus, first coordination of the metal species to the hydroxyl group in intermediate **223** could led to carbocation **224** by C–O bond cleavage. Addition of one molecule of water could then generate the metalated complex **225**, evolving to the experimentally observed enones **222** through sequential demetalation/isomerization processes (Scheme 40). A related mechanistic pathway has been recently proposed by Gao and Xu et al., for the acid-catalyzed synthesis of ketophosphine oxides, although in both cases an alternative mechanistic pathway describing the inverse addition/elimination sequence may not be discarded.^204,205^

**Scheme 41:**
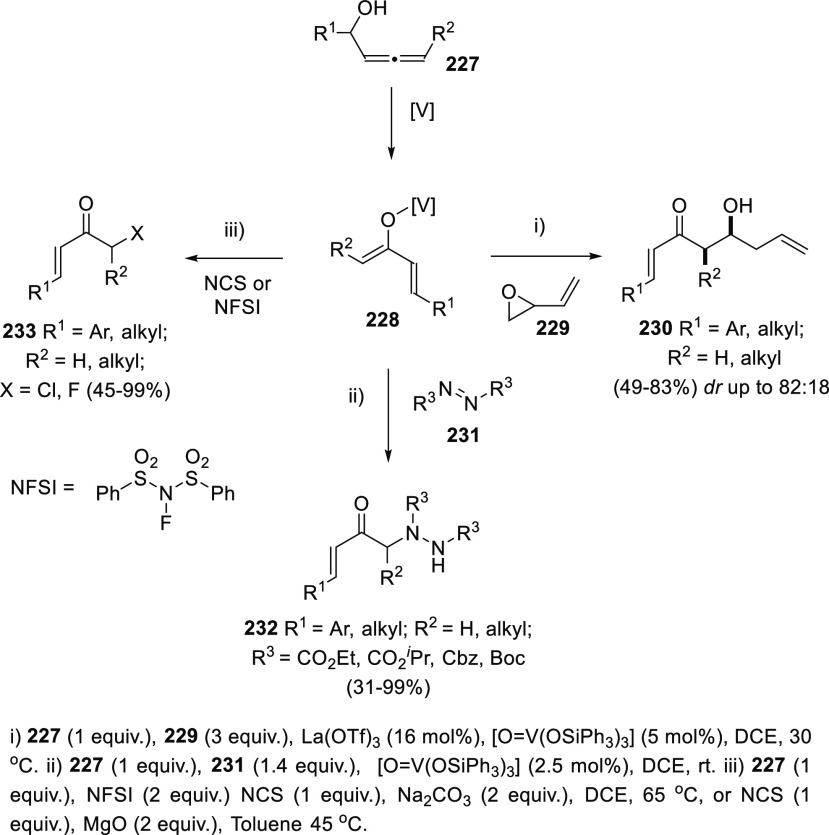
Vanadium-Catalyzed Transformations of α-Allenols

Trost’s research groups have extensively investigated the metal-catalyzed Meyer-Schuster rearrangement of allenols **227** from a different perspective. Instead of delivering the corresponding enones from demetalation/isomerization of species **228**, intermediates **228** were envisioned as coupling reagents with both electrophiles and nucleophiles, providing a wide and diverse family of functionalized ketones. Thus, reaction of vanadium enolate **228** with vinyl epoxides **229** as masked aldehydes in the presence of a Lewis acid, provided aldol products **230** (Scheme 41, right).^206^ Vanadium enolates in the presence of diazocompounds **231** as electrophiles provided ketones **232** from a direct sigmatropic amination reaction (Scheme 41, bottom).^207^ Noteworthy, reaction of allenes with diazocompounds are well-known to yield vinyl diazines through Alder-ene mechanisms.^208,209^ On the other hand, reaction of vanadium enolates **228** with different nucleophile halogen sources such as NCS or NFSI provided the corresponding α-haloketones **233** with moderate to excellent yields (Scheme 41, left).^210^

**Scheme 42:**
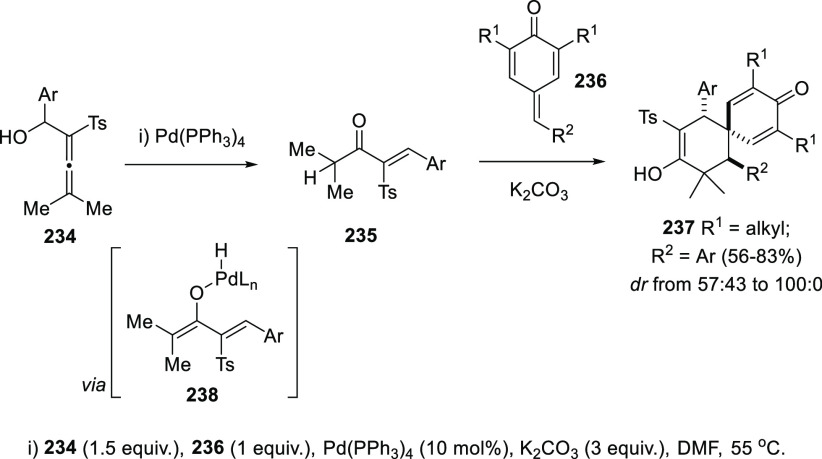
One-Pot Synthesis of Spirocyclic Compounds from Allenols Involving Palladium-Mediated Enone Formation

The allenol-enone metal-catalyzed isomerization has been recently proposed as an intermediate step toward the synthesis of spirocyclic scaffolds in compounds **237**. First, treatment of sulfonyl allenols **234** with a Pd catalyst yielded the corresponding enones **235**, through a 1,3-migration rearrangement via the corresponding metal-enolate **238**. Enones **235** evolved in the presence of a base and *p*-quinone methides **236** to the observed adducts **237** through a cascade Michael-type addition/ring closing reaction (Scheme 42).^211^

**Scheme 43:**
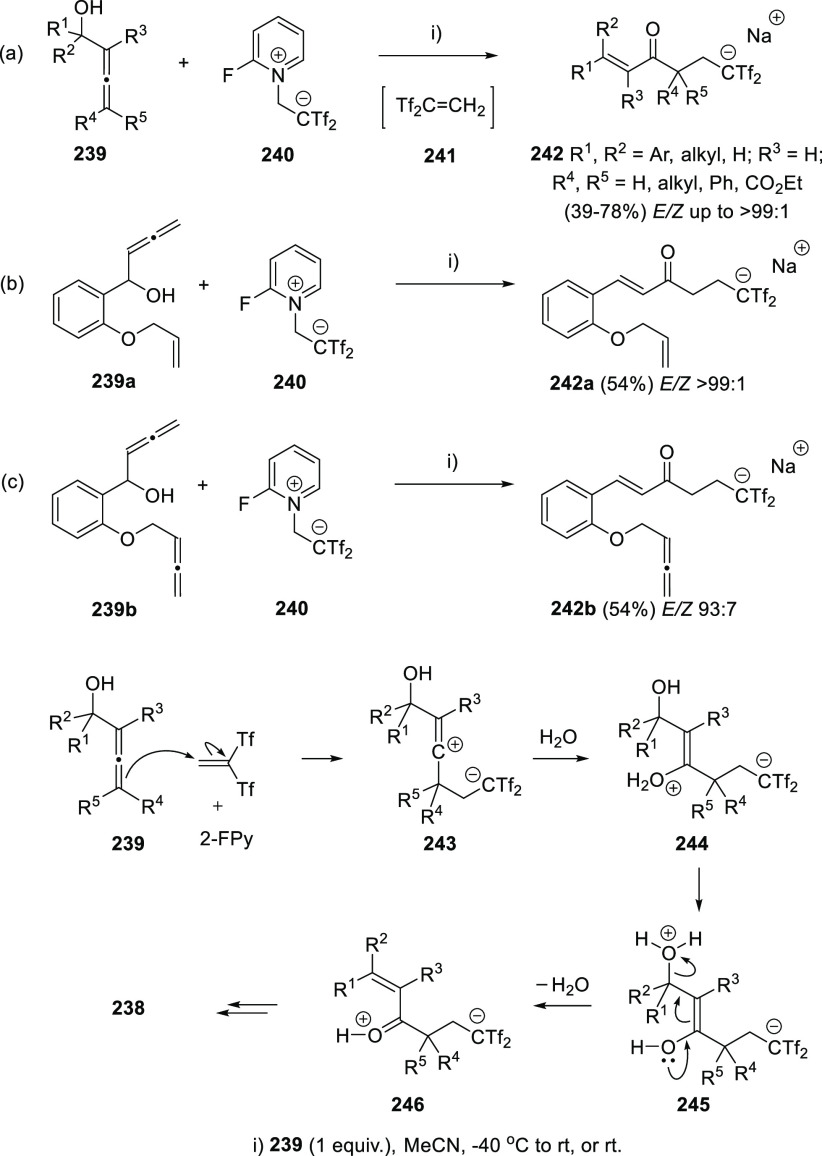
Allenol-enone Transformation by Electrophilic Attack of Bis(triflyl)ethene

Bis(trifyl)enones **242** have been recently prepared from α-allenols **239** unravelling a reversal regioselectivity for the reaction of allenes with electrophiles. Almendros et al. have reported the reaction of allenols **239** with the *in situ* generated strong electrophile Tf_2_C=CH_2_ (**241**).^212^ The reaction proceeds under mild reaction conditions in the absence of catalysts or additives (Scheme 43, reaction a). Interestingly, complete selectivity was found in the reaction of substrates **239a** or **239b** bearing an alkene or an extra allene unit (Scheme 43, reaction b and c, respectively), illustrating the divergent chemical behavior of allenes *versus* allenols. Thus, addition of 2-(2-fluoropyridinium-1-yl)-1,1-bis(triflyl)ethan-1-ide (**240**) to the reaction media spontaneously generates bis(trifyl)ethene (**241**), which undergoes a selective electrophilic attack toward the terminal allenic carbon in **239**, opposite to the commonly reported central carbon atom electrophilic attack. The proposed reaction mechanism is followed by addition of water to the central positively charged carbon in **243** and by a 1,5-proton shift in the resulting species **244** to generate intermediate **245**. Dehydratation would finally yield the experimentally observed enones **242** (Scheme 43, bottom). Computed calculations for the reaction profile supported the mechanistic hypothesis, stating a rare electrophile-mediated transformation of allenols into enones instead of the usual nucleophile-based procedures. Also, the unexpected regioselectivity leaves a door open for a further exploration of the yet intriguing reactivity of the allenol moiety.

**Scheme 44:**
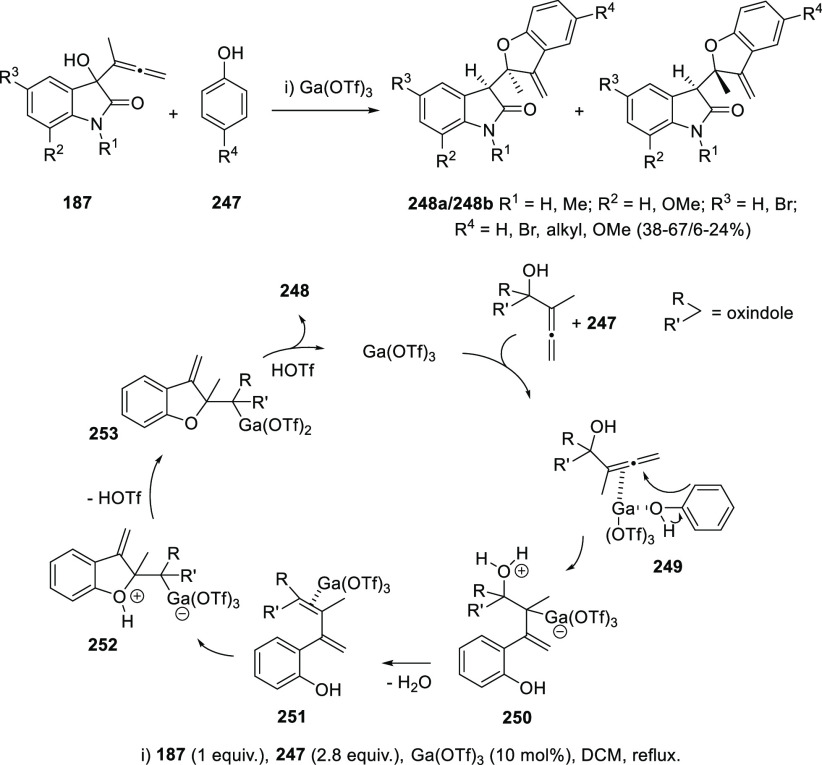
Gallium-Catalyzed Synthesis of Dihydrobenzafurans by Phenol Addition to α-Allenols

The ability of allenols to isomerize into dienes has been applied to more particular transformations in the context of the intermolecular addition of phenols. Ga(OTf)_3_ catalyzes a cascade process from oxindole-based allenols **187** and phenols **247** providing dihydrobenzofuran compounds **248** with moderate to good yields and practical diasetereoselectivities (Scheme 44). A mechanistic proposal to explain this result could start by a double coordination of the Lewis acid catalyst with both the OH unit from the phenol and the inner double bond from the allenol in complex **249**. Then nucleophilic attack from the ortho position of the phenol to the central allenic carbon could generate intermediate **250**. Loss of water would then lead to the diene skeleton **251**, which may undergo intramolecular oxycyclization to build the dihydrobenzofuran system in oxonium ion **252**. TfOH extrusion and demetalation would then provide the observed oxindole-functionalized dihydrobenzofurans **248** (Scheme 44).^213^

##### 3.1.1.2 Synthesis of Enynes

**Scheme 45:**
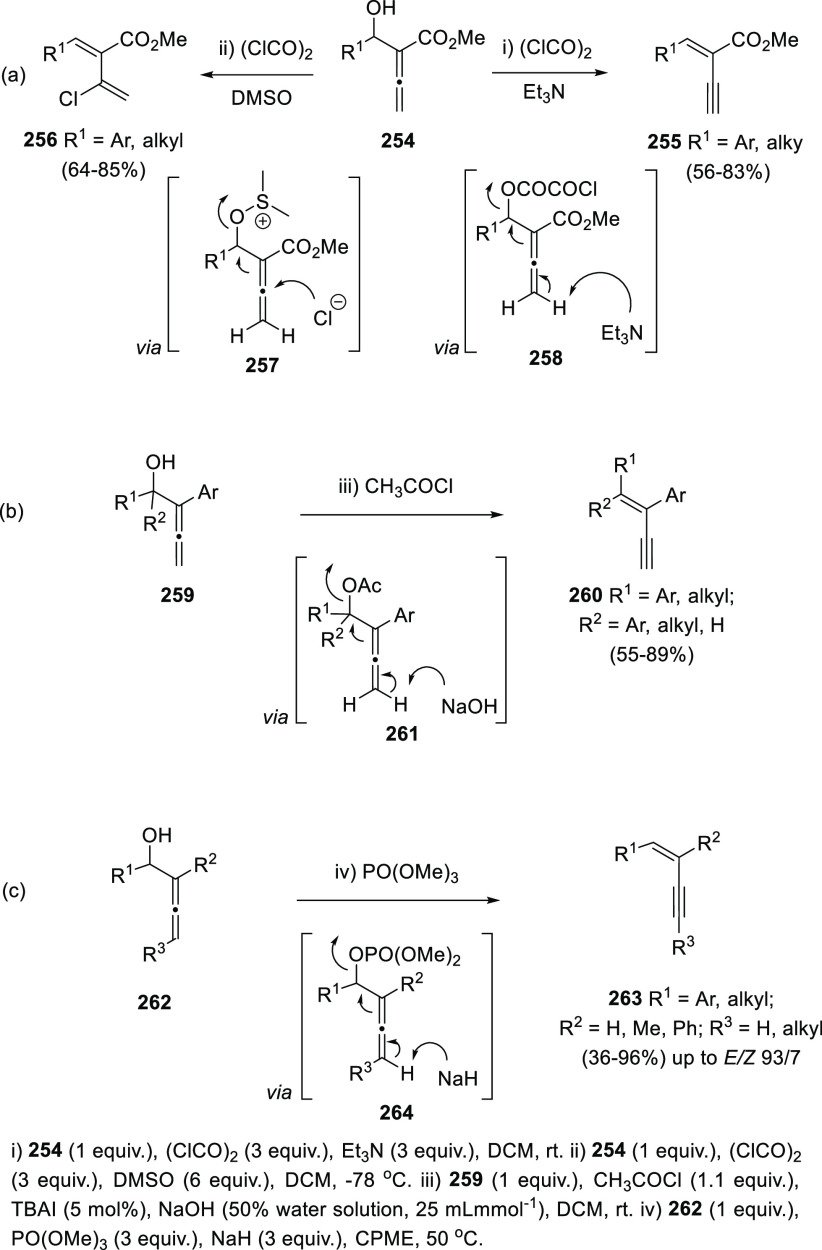
Synthesis of Conjugated Enynes by Metal-Free OH-Activation/Elimination

During the attempts to oxidize the alcohol group in different allenols employing the Swern protocol, Ma and co-workers developed a straightforward methodology for the transformation of α-allenols **254** into conjugated enynes **255** and chlorinated dienes **256**. It was found that the presence of triethylamine as a base notably favored the enyne **255** synthesis, while the presence of DMSO promoted the halogenation/isomerization process toward the diene **256** generation (Scheme 45, reaction a).^214^ Nevertheless, complete selectivity was only achieved in certain cases. Besides, this divergent protocol was restricted to a particular type of 2,3-allenols, namely, allenols having a 2-ethoxycarbonyl substituent. This example clearly illustrates the divergent behavior of allenols under subtle modifications on the experimental reaction conditions.

Alcaide and Almendros research group has presented an alternative procedure for the same transformation avoiding competitive halogenation/isomerization processes. Related OH-activation/elimination strategy was described, employing different reagents and bases. Thus, reaction of differently substituted α-allenols **259** with acetyl chloride and NaOH in aqueous media yielded conjugated *E*-1,3-enynes **260** in good yields and complete regio- and stereoselectivity. In addition, the methodology was compatible with a wide range of functional groups, and extended to hindered tertiary alcohols, which are frequently unreactive in acetylation processes (Scheme 45, reaction b).^215−217^

Sawama and collaborators have developed a phosphate-mediated synthesis of related conjugated *E*-enynes **263** from allenols **262**. Trimethyl phosphate was used as activator of the hydroxyl group, while NaH acted as the basic reagent favoring the enyne synthesis by H_2_ release. Terminal substitution at the allene moiety **262** was tolerated, providing the synthesis of challenging inner alkynes and allowing therefore the extension of the methodology, despite of a slight decrease in the *E*/*Z* selectivity (Scheme 45, reaction c).^218^ Lee has described an efficient protocol for the direct and stereoselective conversion of allenyl acetates into (*E*)-α-ethynyl-α,β-unsaturated esters **255** using DABCO in catalytic amounts (10 mol %).^219^

**Scheme 46:**
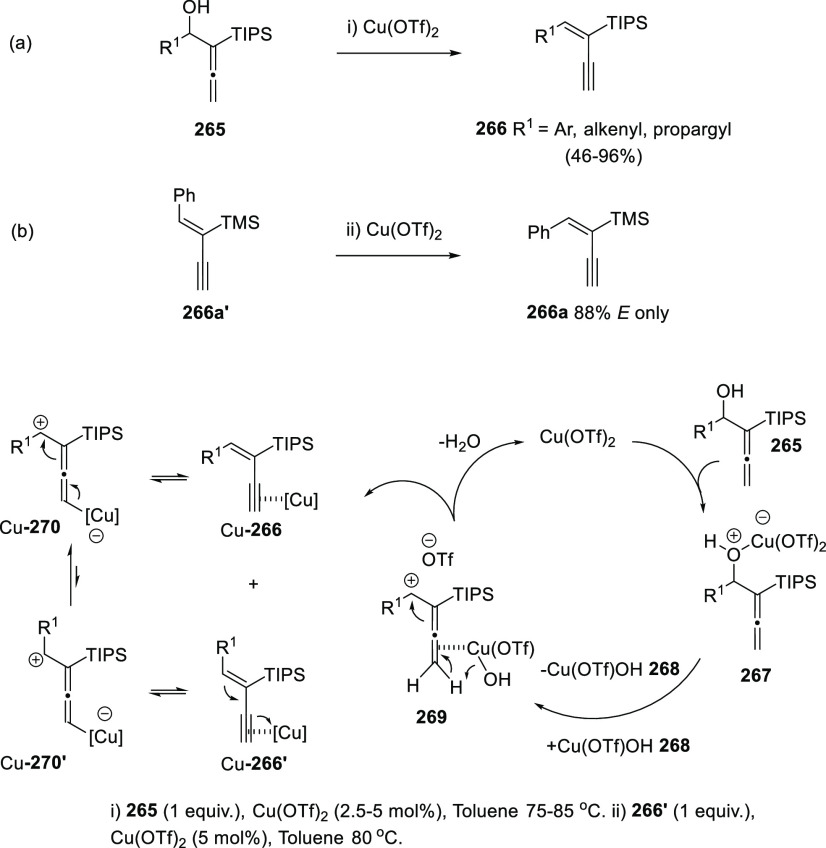
Copper-Catalyzed Synthesis of Enynes from α-Allenols

Metal catalysis has been scarcely reported for the allenol-enyne transformation. One rare contribution described the use of Cu(OTf)_2_ acting both as hydroxyl activator and proton catcher. Thus, α-allenols **265** reacted in the presence of catalytic amounts of copper triflate providing enynes **266** in good to excellent yields and complete *E*-selectivity (Scheme 46, reaction a). The methodology was reported to be useful starting from secondary aryl-substituted allenols and was also extended to the synthesis of dienynes and enedyines. The reaction is proposed to require an initial C–O bond cleavage promoted by the Lewis acidic nature of the metal salt, yielding allenic carbocation species **269** and metal complex Cu(OTf)(OH) **268**. Loss of water would then generate observed enynes **266** and regenerate the metal catalyst Cu(OTf)_2_ (Scheme 46, bottom). Further *Z-E* isomerization experiments on *Z*-enyne **266a′** under similar reaction conditions showed that formation of the observed *E*-enynes **266** should be thermodynamically controlled (Scheme 46, reaction b). Zwitterionic allenyl copper species Cu-**270** and Cu-**270′** are proposed as intermediates in the observed alkene isomerization.^220^

**Scheme 47:**
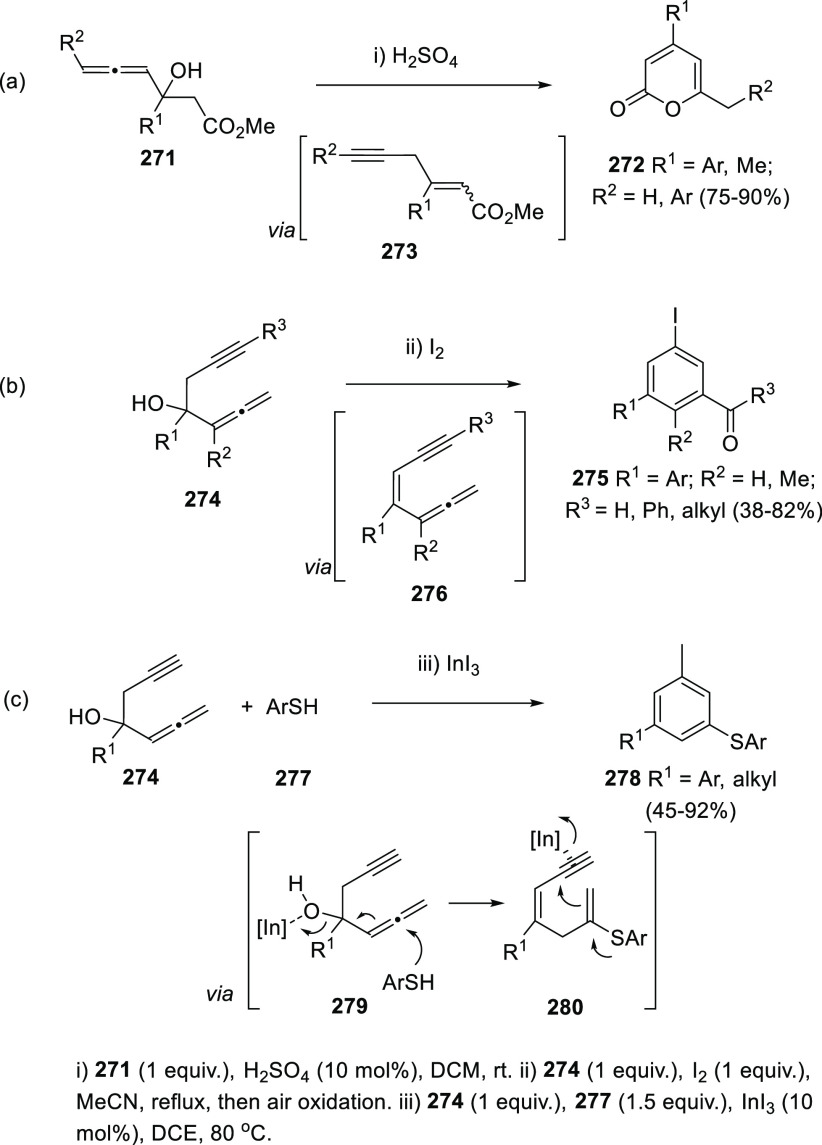
Enynes as Reaction Intermediates in the Synthesis of 2*H*-Pyran-2-ones and Substituted Benzenes from α-Allenols

Enynes have been also proposed as reaction intermediates in the transformation of allenols into the 2*H*-pyran-2-one skeleton, or into substituted benzene rings. 3-Hydroxy-4,5-dienoates **271** can be converted into differently substituted pyranones **272** under protic acid catalysis. Protonation of the hydroxyl group followed by elimination of water is proposed to generate 1,4-enynes **273** as reaction intermediates. Addition of water to the terminal propargylic carbon atom on **273** and further intramolecular transesterification could explain the obtained 2*H*-pyran-2-ones **272** (Scheme 47, reaction a).^221^

Iodine is well-known to efficiently promote the dehydratation of tertiary alcohols. When propargylic allenols **274** were treated with I_2_ in refluxing acetonitrile, iodobenzaldehydes or iodoarylketones **275** were synthesized, depending on the alkynyl substitution in **274**. In this case, allenyl enynes **276** are described as plausible intermediates for this transformation. Further iodine activation of the triple bond followed by allene–alkyne cyclization would furnish the aromatic core in **275**, while the observed carbonyl functional groups may proceed from oxidation of the iodine substituent by atmospheric oxygen (Scheme 47, reaction b).^222^

In a related approach, allenols **274** reacted with thiophenols **277** under transition metal catalysis generating 1,3,5-trisubstituted benzene rings **278**. Ring closing and aromatization reaction is triggered by the nucleophilic addition of thiophenols **277** to the central allenic carbon in intermediates **279**. InI_3_ exhibited a dual behavior as σ- and π-acid because it was used as metal source, activating both the hydroxyl group in **279** and the alkyne functional group in enyne intermediate **280** (Scheme 47, reaction c).^223^

##### 3.1.1.3 Allene Transfer Reactions

The cleavage of the C–O bond in the allenol skeleton after the appropriate hydroxyl activation may result in the generation of an allenic carbocation. When this kind of carbocation is trapped by a nucleophile without isomerization or rearrangement, the overall process results in a formal allene transfer reaction. Because of the high reactivity of the allene functional group, transfer reactions where the allene moiety remains unaltered are still rare. Nevertheless, recent reports have appeared dealing with this transformation and its synthetic applications.

**Scheme 48:**
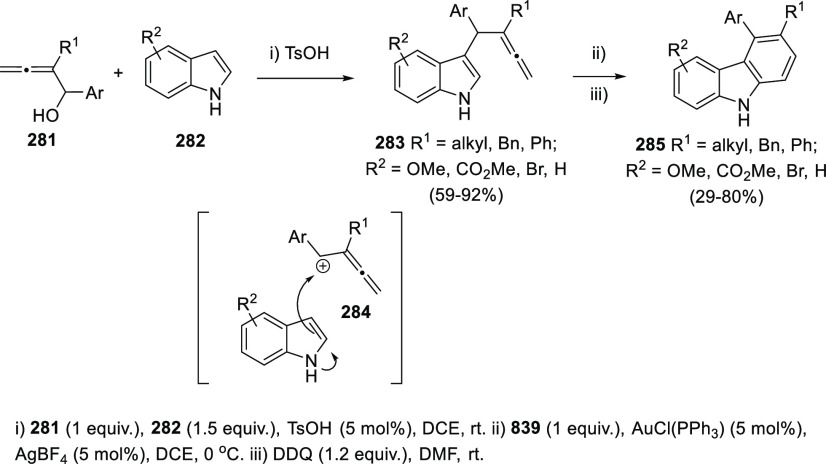
Synthesis of 3-Allenyl Indoles through Acid-Mediated Allene Transfer and Synthetic Applications

Ma and collaborators have used diverse α-arylallenols **281** as precursors of stabilized allenic carbocations **284** under acid catalysis. Thus, treatment of **281** with *p*-toluenesulfonic acid in the presence of indoles **281** as nucleophiles, yielded 3-allenyl indoles **283** with moderate to excellent yields through an allene transfer process. 3-Allenyl indoles **283** were employed as precursors for the synthesis of a family of heteroaromatic compounds **285** showing the carbazole scaffold.^224,225^ The synthetic strategy included a carbocyclization process catalyzed by gold, followed by oxidation of the resulting dihydrocarbazoles with DDQ to yield the fully aromatic structure in compounds **285** (Scheme 48).

**Scheme 49:**
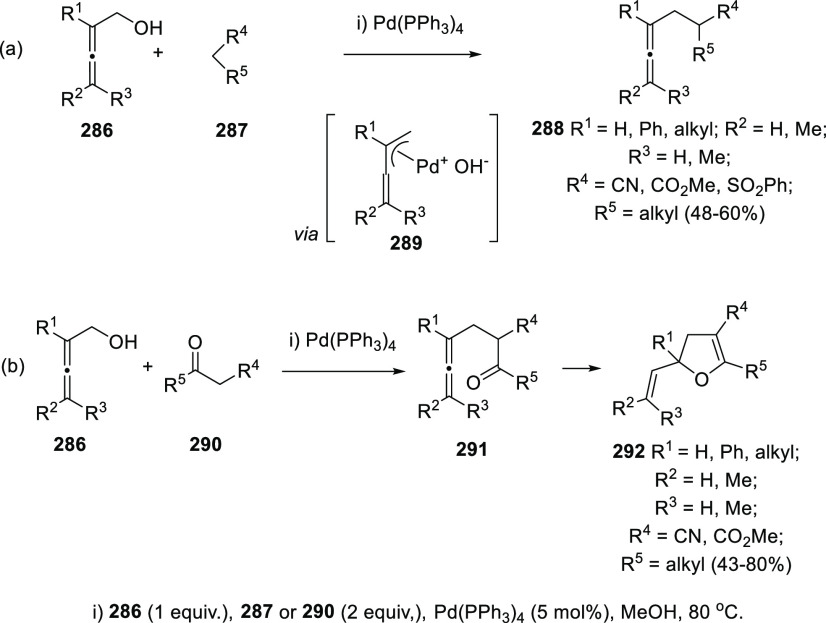
Palladium-Catalyzed Allene Transfer and Application toward the Synthesis of Vinyl Dihydrofurans

Tsukamoto’s research group has developed a metal-catalyzed variant of the allene transfer reaction, employing primary alcohols **286** and diverse pronucleophiles (**287**, **290**). The reaction is reported to possible proceed through a π-allyl palladium complex intermediate **289**, generated by the oxidative addition of Pd(0) species to allenols **286**. Nucleophilic addition toward the unsubstituted carbon on the π-allyl complex would furnish the new allene structure **288** (Scheme 49, reaction a). When the reaction takes place with ketones **290** bearing electron-withdrawing groups as pronucleophiles allenones **291** are obtained, which in situ undergo a palladium-catalyzed oxycyclization providing vinyl dihydrofurans **292** (Scheme 49, reaction b).^226^

**Scheme 50:**
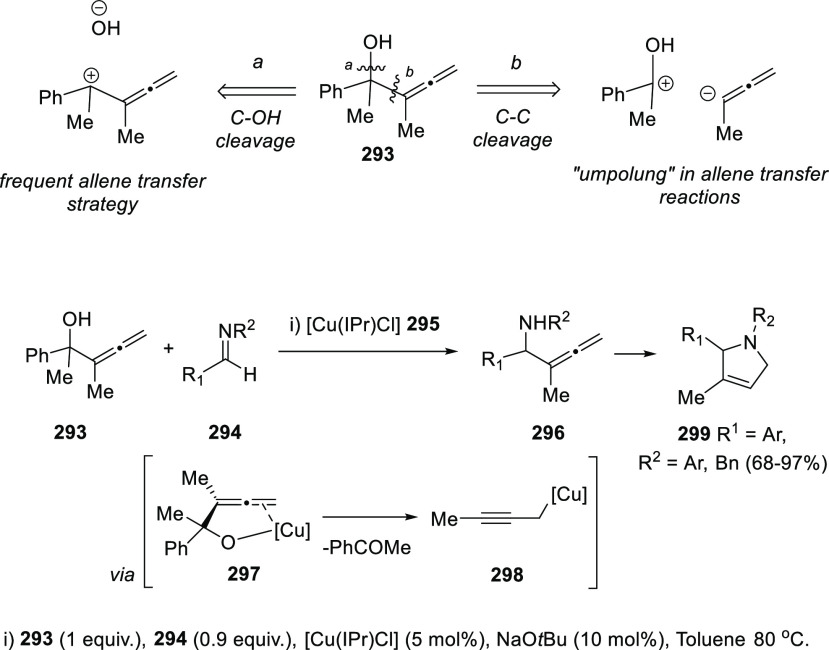
Copper-Catalyzed Allene Transfer for the Synthesis of Allenamines and *In Situ* Aza-Cyclization Reactions

Oshima and collaborators have reported a different strategy to achieve the allene transfer process. Copper carbene complexes **295** have shown great activity promoting a challenging C(sp^3^)-C(sp^3^) bond cleavage (Scheme 50, top, path *b*) in allenol structures **293**, instead of the more frequent C–OH dissociation (Scheme 50, top, path *a*). Coordination of the metal species with both the OH group and the cumullene is reported to generate metal intermediate **297**, which may evolve through PhCOMe elimination providing copper propargyl complex **298**. This strategy induced an umpolung on the normal electronic charges in allene transfer processes, allowing the reaction of allenols with electrophiles such as imines **294**. As a result, allenyl amines **296** were synthesized and *in situ* submitted for aza-cyclization reaction yielding the pyrrole scaffold in compounds **299** with good to excellent yields (Scheme 50).^227,228^

#### 3.1.2 OH as a Leaving Group in the Last Stages of the Reaction

Although less frequent, the C–OH bond dissociation can happen at the last stages of the reaction pathway, opposite to what has been so far reported in diene, enone, enyne or allene transfer procedures. Late OH release takes normally place in the form of dehydration leading to aromatic or conjugated systems, and it usually constitutes the driving force of the transformation.

This methodology has been extensively used for the synthesis of different structures exhibiting the carbazole motif, a natural occurring alkaloid showing a wide range of biological and pharmacological activities. Both platinum and gold catalysis have been found to catalyze the carbocyclization/dehydration of indole-tethered allenols to yield the carbazole skeleton in a highly efficient manner.^229−231^ Ma and collaborators have invested much effort in developing synthetic routes to carbazole-based natural products through this approach, later discussed in the natural products section.^232,233^

**Scheme 51:**
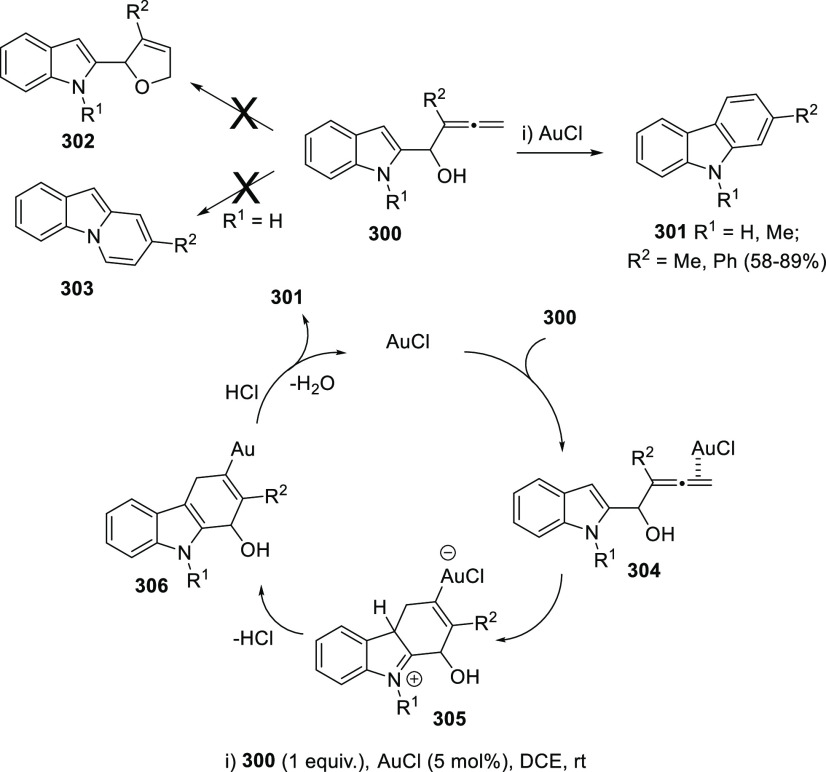
Au-Catalyzed Synthesis of Carbazoles from Indole-Tethered Allenols

The Alcaide and Almendros research group has focused its research in this field on the mechanistic insights of this transformation under gold and palladium catalysis. Indole-tethered allenols **300** may exhibit three possible reaction sites, leading therefore to the corresponding carbo-, oxy-, or aza-cyclization products **301**, **302**, and **303**, respectively. Despite the ability of gold salts to promote oxy-cyclization reactions, complete selectivity toward the carbo-cyclization process (compounds **301**) was found in the presence of AuCl as metal catalyst (Scheme 51, top). The transformation succeeded for both methyl- and sterically hindered phenyl-substituted allenols **300**, leading to the carbazole core **301** with good yields. The reaction mechanism was proposed to start with coordination of the metal to the terminal allenic bond, followed by a *6-endo* carboauration process generating the zwitterionic vinyl gold specie **305**. Loss of HCl would then lead to the neutral complex **302**. A final dehydration and protodemetalation step should furnish the experimentally observed carbazoles **301** and return AuCl to the catalytic cycle (Scheme 51, bottom).^234^

**Scheme 52:**
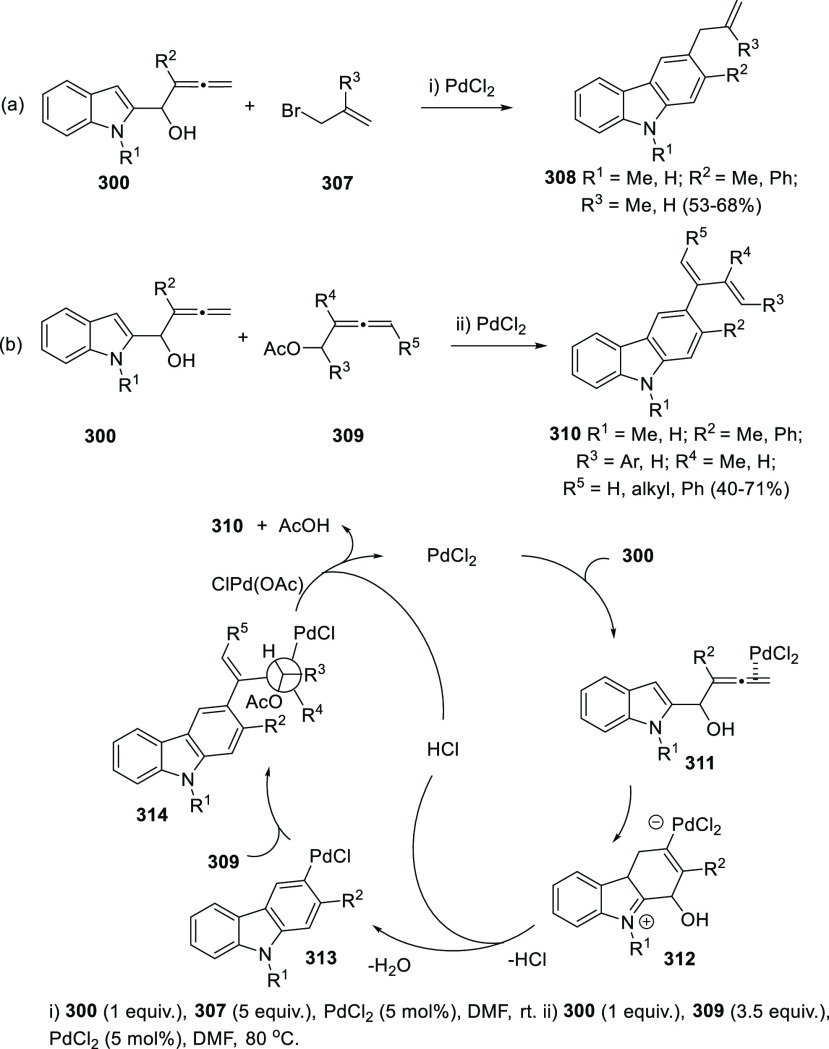
Palladium-Catalyzed Cross-Coupling Reactions of Indole-Tethered Allenols and Allyl Bromides or Acetyl Protected Allenols

Taking advantage of the more π-coordinating nature of palladium ions, a tandem reaction including a similar carbo-cyclization process followed by cross-coupling reactions of allenols **300** with allyl bromides **307** was envisioned (Scheme 52, reaction a). Different allyl-substituted carbazoles **308** were synthesized in good yields and complete regioselectivity.^234^ Noteworthy, when related cross-coupling reaction was performed in the presence of a second allenic unit **309**, pharmacologically attractive 3-(buta-1,3-dienyl) carbazoles **310** were obtained (Scheme 52, reaction b). Yields were moderate to good and a wide number of allenols bearing a different pattern of substitution were reactive under those conditions. In addition, the transformation took place in a complete chemo- regio- and stereoselective manner, showing a previously unreported cross-coupling reaction of two allenic moieties including a carbocyclization process. Interestingly, two allenol units **300** and **309** showing a different chemical behavior can be found in this reaction. Indole-tethered allenols **300** behaved as π-activated alcohols where the late C–OH cleavage leads to aromatic rings through dehydration. On the other hand, acetyl protected allenols **309** behaved as activated alcohols where nucleophilic addition toward the central allenic carbon leads to the diene skeleton, as previously illustrated in prior sections. The authors proposed a mechanistic pathway starting from coordination of the palladium ion to the terminal allenic double bond to give complex **311**, followed by *6-endo* carbopalladation generating vinyl palladium species **312**. HCl extrusion and dehydration would provide palladacarbazole **313**. Then cross-coupling reaction toward the central allenic carbon of the acetyl-protected allenol **309** would lead to intermediate **310**. Observed butadienyl carbazoles **310** could be eventually obtained by deacetoxy palladation of intermediates **314**, regenerating the catalytic species after loss of one molecule of AcOH.^235^

**Scheme 53:**
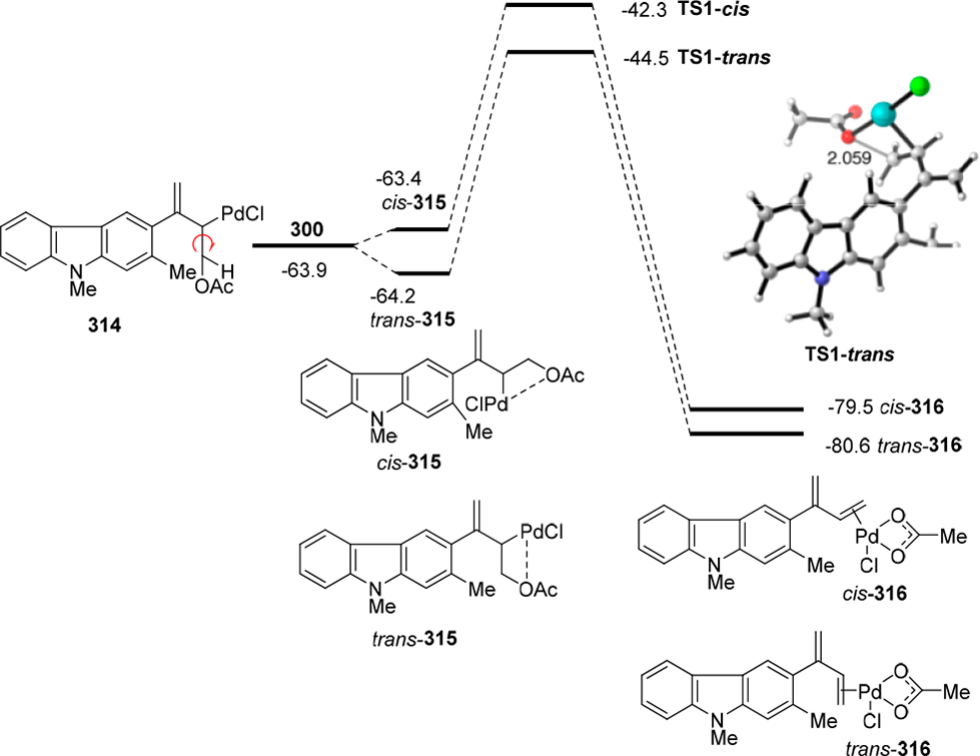
DFT Computed Reaction Profile for Deacetoxypalladation Step in Allenol–Allenol Cross-Coupling Reaction Relative free energies are given in kcal mol^–1^.

DFT calculations revealed a computed carbocyclization reaction profile notably lower in energy compared to the oxycyclization process from indole-tethered allenols **300**, supporting the chemoselectivity observed in the first step of the tandem reaction. Also, the complete stereoselectivity observed in the diene generation can be explained considering the computed results for the depalladation step (Scheme 53). Free rotation along the C–C single bond in intermediate **314** could lead to both *cis***-315** or the more stabilized *trans***-315** complex. Demetalation step is calculated to proceed through a lower energy barrier from *trans***-315** adduct, yielding the also more favored *trans***-316** coordination complex. Thus, the more plausible reaction pathway is the kinetically and thermodynamically controlled trans-deacetoxypalladation process via transition state **TS1-***trans*.

**Scheme 54:**
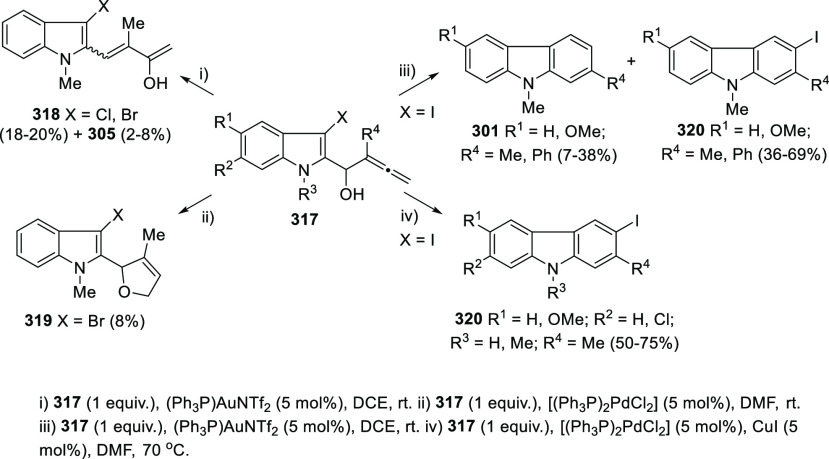
Divergent Reactivity on 3-Halo-(indol-2-yl)-α-allenols under Metal Catalysis

3-Halo-(indol-2-yl)-α-allenols **317** revealed an intriguing reactivity pattern, showing divergent behavior depending on the halide substitution. 3-Chloro- and 3-bromo-indoles reacted with gold salts to yield dienes **318** via a 1,3-hydroxyl migration in complex reaction mixtures (Scheme 54, top left). Also, traces of oxycyclization products were observed. Interestingly, palladium catalysis only provided dihydrofuran systems **319** in low yields when 3-bromo-(indol-2-yl)-α-allenols **317** were employed (Scheme 54, bottom left). Noteworthy, when iodine-substituted indoles **317** were submitted to gold-catalyzed conditions a different reactivity was observed, obtaining mixtures of the previously reported carbazole structures **301** along with novel iodocarbazole compounds **320** (Scheme 54, top right). Complete selectivity toward the iodocarbazole skeleton was achieved under palladium conditions, yielding structures **320** with moderate to good yields (Scheme 54, bottom right). Opposite to normal metal catalyzed reactions from aryl halides where the halogen atom is lost during the reaction course, the observed iodine reincorporation into the final skeleton means an atom-economic improvement and unravels an unreported reaction mechanism.^236^

**Scheme 55:**
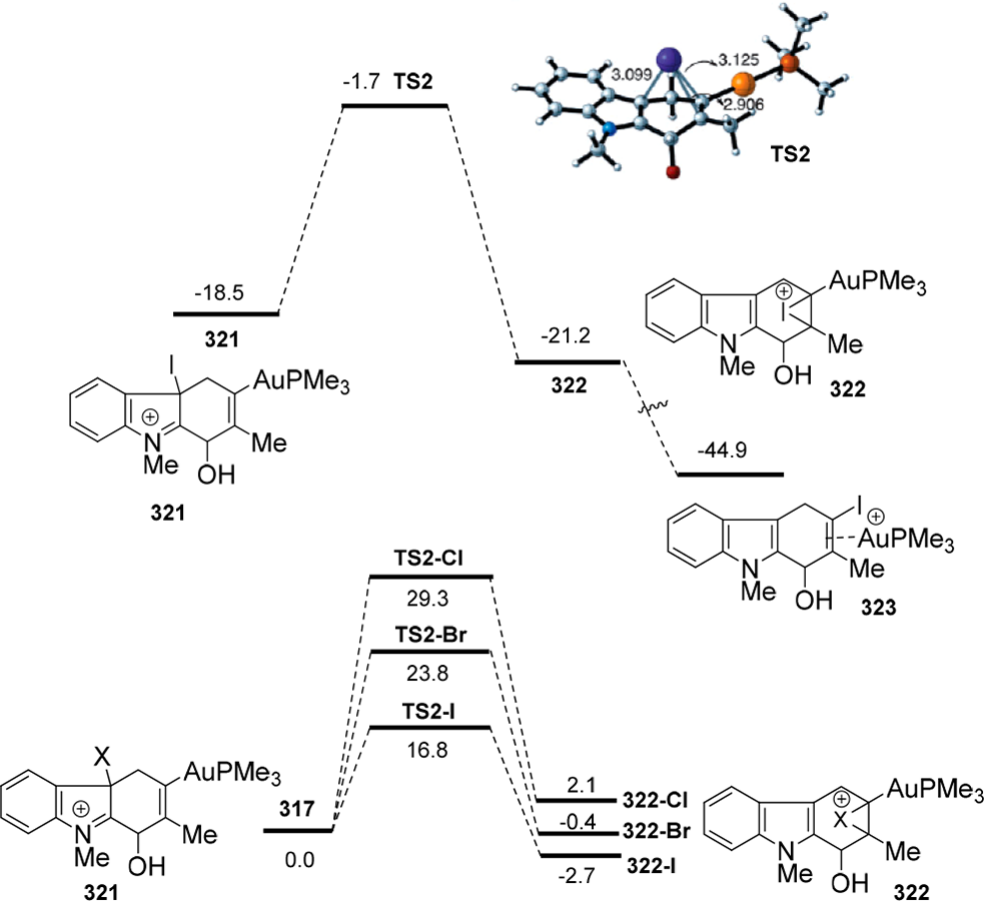
DFT Computed Reaction Profile for the 1,3-Halogen Migration Step in Metal-Catalyzed Iodocarbazole Synthesis Relative free energies are given in kcal mol^–1^.

DFT calculations supported a 1,3-intramolecular iodine migration from dihydrocarbazole intermediate **321** to generate the corresponding iododihydrocarbazole **322**. Iodonium cation **322** is proposed as the most favorable intermediate to achieve this transformation (Scheme 55, top). Also, computed reaction profile comparison of the migratory ability of chlorine, bromine, and iodine derivatives **321** supported the observed results. Activation barriers for the intramolecular 1,3-migration process are much higher for Br and Cl-substituted indoles (**TS2-Br** and **TS2-Cl**, respectively), leading therefore to diene adducts **318** or dihydrofurans **319**. On the other hand, a lower energy barrier for the 1,3-iodine migration through transition state **TS2-I** facilitates the halogen recycling toward iodocarbazoles **320** (Scheme 55, bottom).

**Scheme 56:**
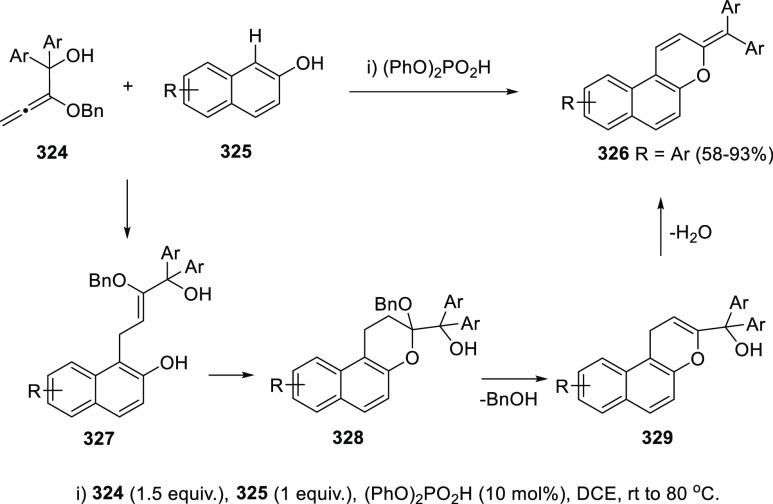
Acid-Catalyzed Synthesis of Naphthols

In a different approach, a wide family of naphthopyrans exhibiting large π-conjugation have been prepared. Naphthol (**325**) and related polyaromatic compounds reacted with conveniently substituted alkoxyallenes **324** in the presence of acid catalysts through a cascade process, providing naphthopyrans **326** in moderate to excellent yields. The reaction sequence includes a first allylation step to generate allyl naphthols **327**, followed by oxycyclization, loss of one molecule of HOBn to build intermediate **315**, and final dehydration to give the observed polyaromatic structures **326**. Late C–OH cleavage inducing the extended conjugation in systems **326** is assumed as the driving force of the overall transformation (Scheme 56).^237,238^

### 3.2 Allenols as Bidentate Nucleophiles–Electrophiles

The inherent reactivity of hydroxyl groups and activated allene moieties as nucleophiles and electrophiles respectively, has prompted the oxycyclization reaction as one of the most extensively and traditionally reported transformation. The *5-Endo-trig* cyclization leading to the dihydrofuran skeleton, *6-endo-trig* processes providing dihydropyran motifs and cocyclization processes such as cyclocarbonilations leading to lactones have been widely employed in organic synthesis, exhibiting diverse applications in catalysis or natural products preparation.^239−250^ During the past decade the investigations in this field have been focused on developing new and more selective strategies for the oxycyclization reaction, greener and more economic procedures, and more sophisticated transformations for the synthesis of challenging molecular targets through tandem processes. Opposite to the π-activated alcohol reactivity discussed in the previous section, bidentate reactivity is not limited to α-allenol systems. Although less frequently reported, reactivity from β-, γ-, and δ-allenols will be also discussed. In addition, the oxycyclization of allenols has been largely employed as model reaction for the design of new catalysts with improved reactivity.

**Scheme 57:**
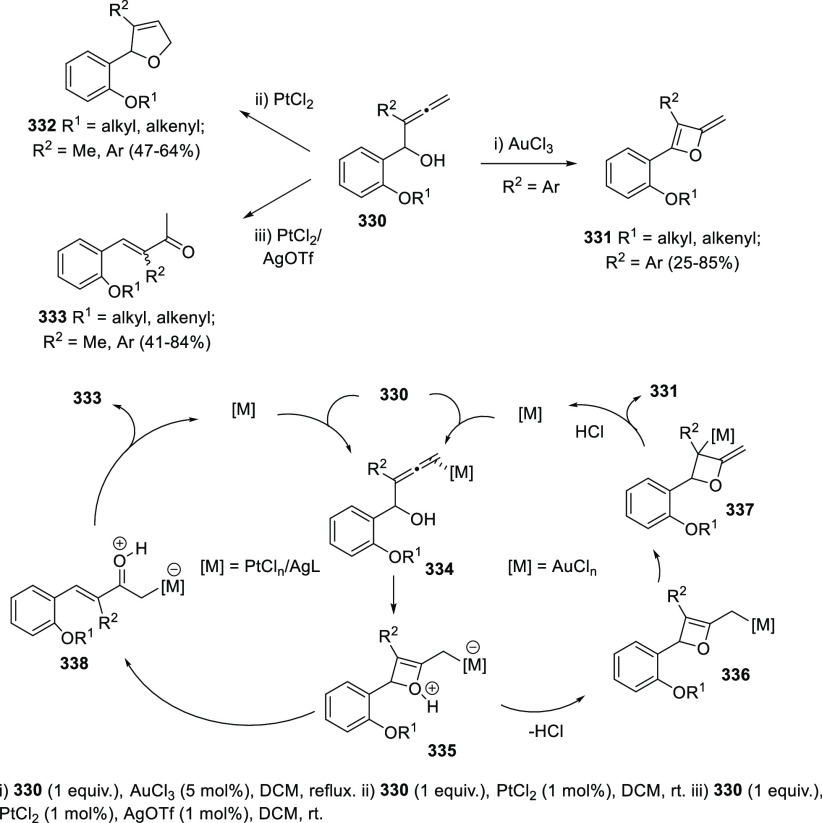
Metal-Catalyzed *4-exo-dig *versus* 5-endo-dig* Oxycyclizations of α-Allenols

Alcaide and Almendros research group has devised a dual selectivity strategy for the oxycylization of α-allenols based both on the metal catalyst and on the allene substitution. To achieve a rare *4-exo-dig vs* the most common *5-endo-trig* cyclization, aryl-substituted allenes **330** were synthesized to induce an extra stabilization on the η^2^ gold intermediate complexes and to promote the nucleophlic attack toward the central allenic carbon. Interestingly, the selectivity toward the oxetene adducts **331** was improved by raising the temperature, indicating thermodynamic control over the *4-exo-trig* products **331** (Scheme 57, top right). DFT calculations also supported this result, pointing to a reaction mechanism proceeding through a zwitterionic oxetene gold complex **335**, which after loss of HCl and 1,3-gold migration could provide the rearranged neutral oxetane **337**. A rare β-hydride elimination in gold catalysis could explain the observed oxetene adducts **331** (Scheme 57, bottom right).^251^

The use of platinum salts in substrates **330** revealed a divergent behavior toward the cycloetherification process. While PtCl_2_ cleanly provided the expected dihydrofuran systems **332**, the addition of AgOTf promoted a dramatic change in the reactivity yielding exclusively substituted enones **333** (Scheme 57, top left). Supported on the precedents reported by the same group, the authors proposed a reaction mechanism passing by similar oxetene intermediates **335**. A ring opening process instead of metal-migration to yield complex **338**, followed by deprotopalladation would furnish the observed enones **333** (Scheme 57, bottom left). In addition, control experiments indicated the active role of silver ions in the reaction mechanism, through a yet not fully understood bimetallic catalytic species.^252^

**Scheme 58:**
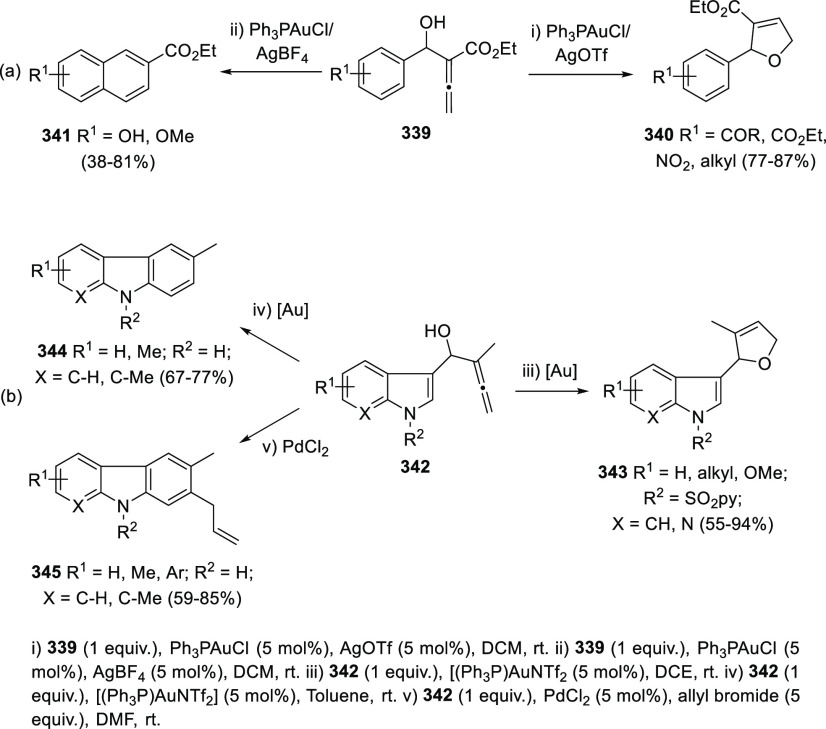
Cycloetherification *vs* Carbocyclization under Gold and Palladium Catalysis

Cycloetherification *versus* carbocyclization/dehydration has also been recently studied as a substrate-dependent methodology in metal-catalyzed experiments. α-Aryl-α′-hydroxyallenic esters **339** undergo *5-endo* oxycyclization to yield aryl-substituted dihydrofurans **340** when the aryl moiety is decorated with electron withdrawing or mild electron donating groups (Scheme 58, reaction a, right).^253^ Nevertheless, and under similar reaction conditions, allenoates **339** were previously reported to provide functionalized naphthalene derivatives **341** when the aryl unit bears strong electron donating substituents (Scheme 58, reaction a, left). In this case, the enhanced nucleophilicity of the aromatic ring seems to be responsible for the reactivity switch of hydroxyallenic esters **339** toward a sequential carbocyclization/dehydratation, which is also favored by the extra stability of final aromatic compounds **341**.^254^ A related approach from novel (indol-3-yl)-α-allenols **342** on the selective oxycyclization *versus* carbocyclization/aromatization process which can be easily modulated by changing the substitution on the pyrrolic nitrogen in **342** has been described. Thus, deactivated indoles yielded dihydrofuran derivatives **343** by gold-catalyzed cycloetherification processes with moderate to excellent yields, also allowing a wide functional group compatibility (Scheme 58, reaction b, right). On the other hand, *NH*-indoles **342** provided the carbazole skeleton **344** through a previously described tandem carbocyclization/dehydratation reaction. Moreover, cross-coupling reaction in the presence of allyl bromides and palladium catalysts generated the corresponding allyl-carbazoles **345** in good yields and in a regio- and chemo-selective fashion (Scheme 58, reaction b, left).^255^

Allenyl acetates have also been used as competent substrates in cyclization reactions. Thus, Zhang reported a gold-catalyzed formal [3 + 3] benzannulation strategy for the preparation of polysubstituted benzyloxy arenes from 4-(benzyloxy)hexa-1,4,5-trien-3-yl acetates,^256^ while Mukai described a protocol for the formation of indole-2,3-quinodimethanes utilizing a potassium carbonate-promoted aminocyclization of 2-(2-((*tert*-butoxycarbonyl)amino)aryl)buta-2,3-dien-1-yl acetates with concomitant acetic acid release^257^ In an earlier report^258^ Cha prepared ethynyl-substituted ciclopropanes through the reaction of β-allenyl tosylates by basic treatment with LDA, in a cyclization which is supposed to proceed by sequential deprotonation of the internal allene hydrogen and cyclization with concurrent 4-methylbenzenesulfonic acid loss.

Different reports on the oxycyclization reactions selectivity including a competitive ring expansion *versus* cycloetherification in 3-allenyl-3-hydroxyindolones,^259^ counterion-controlled double bond isomerization in the oxycyclization of δ-allenols,^260^ or substrate-dependent *5-endo*- **versus* 6-endo*- in phosphorus-based allenols have appeared.^261^

**Scheme 59:**
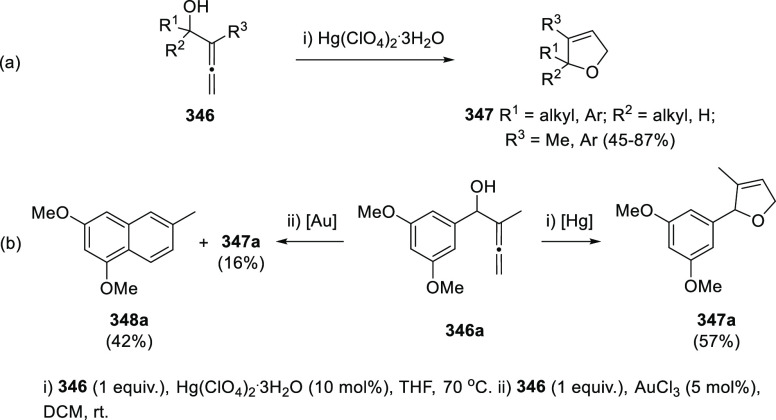
Hg-Catalyzed Cycloetherification of α-Allenols

Much effort has also been invested in developing different procedures to achieve the cycloetherification reaction from more economic or greener perspectives. In this regard, mercury salts were found to catalyze the oxycyclization of α-allenols **346** in a cheaper approach, compared to the most frequent precious metal-based methodologies. Thus, inexpensive and water-tolerant Hg(ClO_4_)_2_·3H_2_O provided the dihydrofuran skeleton **347** in good yields and wide scope, as it happens in sterically hindered tertiary allenols (Scheme 59, reaction a). In addition, complete selectivity toward the oxycyclization was observed even when electron rich aryl allenols **346a** were employed, which normally led to mixtures **348a/347a** under gold catalysis (Scheme 59, reaction b).^262^

**Scheme 60:**
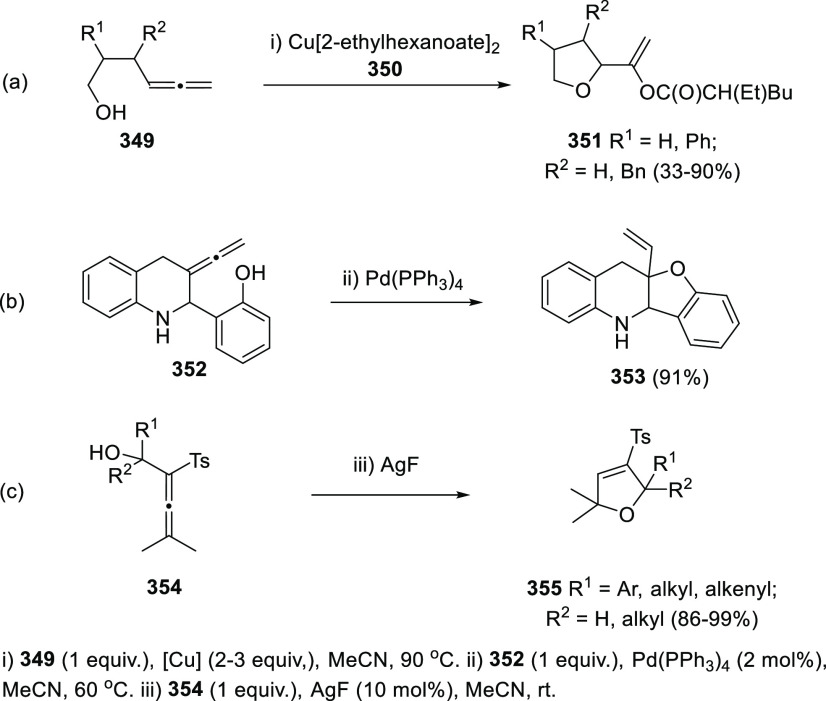
Metal-Mediated Cycloetherification of Diverse Allenols

Recent metal-based alternatives to the classic cycloetherification reaction include the use of stoichiometric amounts of copper carboxylates **350** for the dioxygenation of allenols **349**. Dihydrofuran systems **351** decorated with the vinyl carboxylate ester functionality were obtained through a *5-exo-trig* cyclization of γ-allenols (Scheme 60, reaction a).^263^ Exocyclic γ-allenol **352** has been also described to react through a *5-exo-trig* cyclization path in a Pd(0) catalyzed reaction (Scheme 60, reaction b).^264^ On the other hand, silver fluoride has been effectively used for the *5-endo-trig* oxycyclization of highly substituted and sterically encumbered α-allenols **354**, leading to dihydrofurans **355** exhibiting excellent yields and wide group compatibility (Scheme 60, reaction c).^265^

**Scheme 61:**
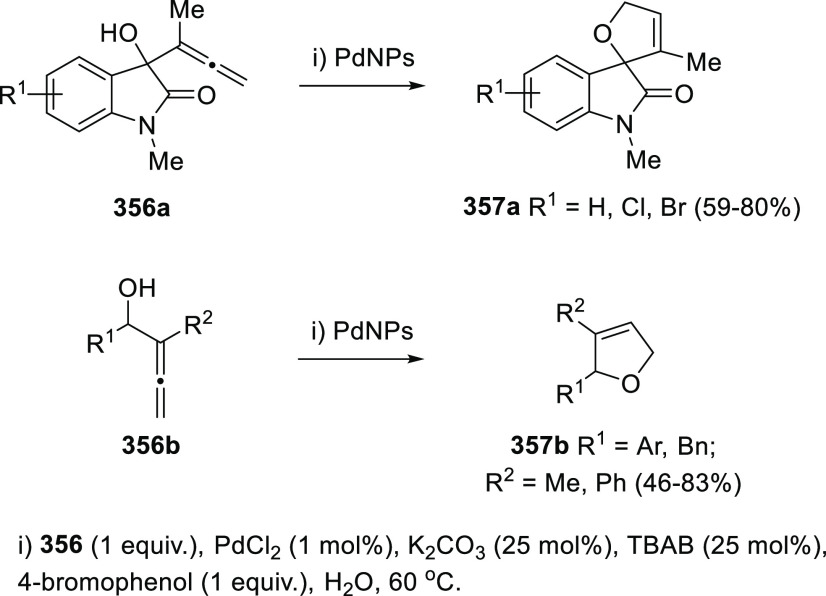
Heterogeneous Palladium-Catalyzed Oxycyclization of α-Allenols

Palladium nanoparticles (PdNPs) have shown high efficiency catalyzing the oxycyclization of differently substituted α-allenols **356a** and **356b**. Preformed nanoparticles using PdCl_2_ as metal source, K_2_CO_3_ as reducing agent and TBAB as stabilizer led to a wide family of dihydrofuran systems **357a** and **357b** in similar yields as the ones reported through the classic precious-metal approaches in homogeneous conditions (Scheme 61). Interestingly, phenols were needed as additives to achieve higher yields and conversions. TEM analysis showed an average particle size of 2.2 nm, and recycling experiments indicated a slight loss of the catalytic activity of solely 8% after four cycles, pointing to a low grade of bleaching in the catalytic system. Although higher temperatures are required compared to analogous homogeneous strategies, the lower catalyst loading of 1 mol %, the higher recycling performance, and the use of water as solvent establish the PdNP methodology as an effective greener procedure.^266^

**Scheme 62:**
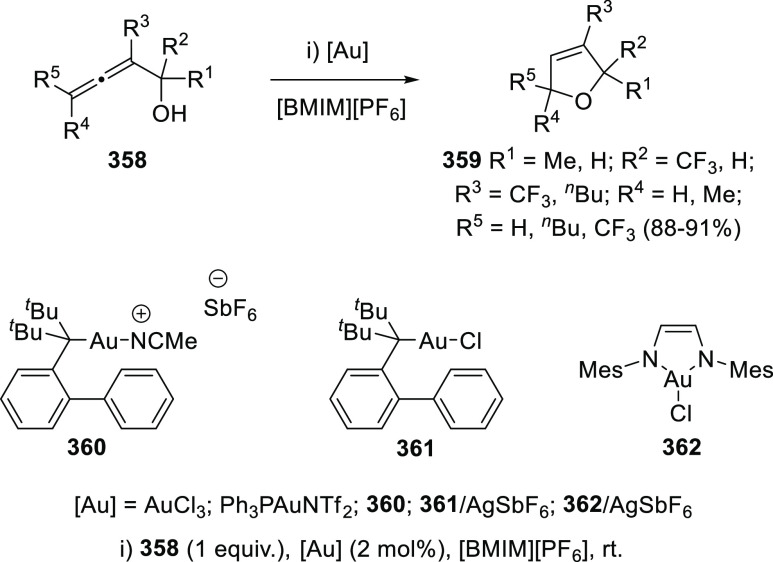
Cycloetherification of Trifluoromethylated Allenols in Ionic Liquids

An alternative strategy for the catalyst recycling has been recently reported by Krause and collaborators, based on the use of gold catalysis in ionic liquids. Different trifluoromethylated allenols **358** were selected as model substrates for the *5-endo-trig* cyclization reaction using both cationic and neutral gold species (Scheme 62). 1-Butyl-3-methylimidazolium hexafluorophosphate [BMIM][PF_6_] was selected as the best ionic media, allowing full conversions in most of the cases. Allenols **358** formed droplets when added to [BMIM][PF_6_], creating a heterogeneous system and allowing the gold catalyst recovering. A low decline of the yield in just 8% after 5 runs shows the practicality of the procedure. Also, mechanistic studies revealed a remarkable kinetic change when allenols bearing a R^4^ = CF_3_ substituent were submitted to Au-cycloeteherification conditions. In this case, formation of the π-complex was identified as the rate-determining step, opposite to the most habitual protodeauration reaction as the regulating step in gold-catalyzed oxycyclizations. This change is probably due to the stronger deactivating effect of the CF_3_ group when located at the terminus of the allenic moiety.^267^

**Scheme 63:**
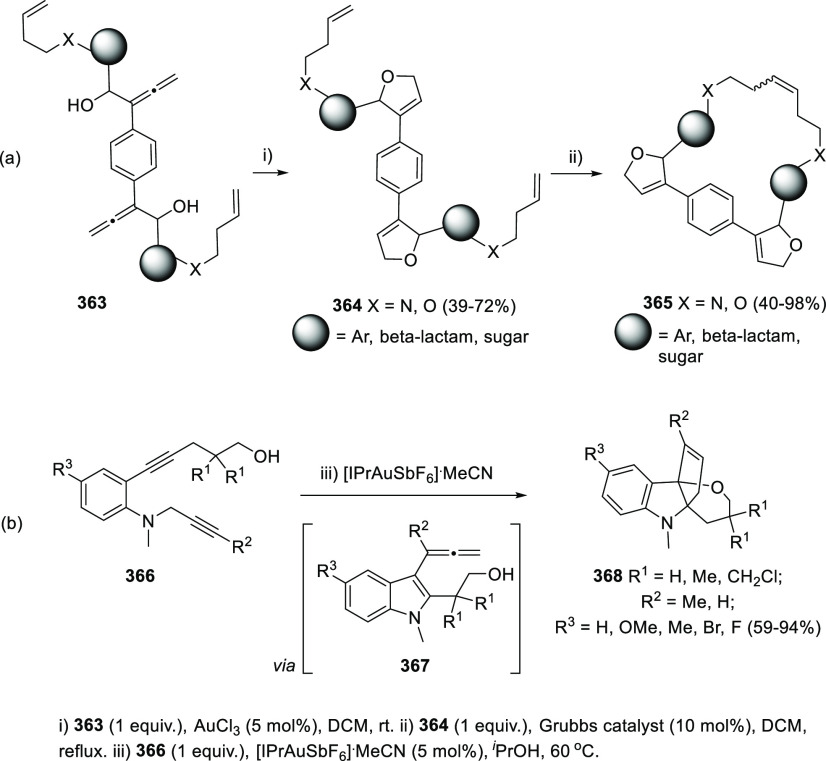
Gold-Catalyzed Oxycyclization of Allenols for the Synthesis of Cyclophanes and Tetracyclic Indolines

Gold catalyzed-cycloetherification has also been involved in the preparation of different compounds exhibiting an added value as molecular materials or naturally occurring alkaloid fragments. The *5-endo-dig* cyclization of α-allenols **363** has been employed as one of the key steps en route to cyclophanes **365**. Thus, double oxycyclization of allenols **363** led to bis(dihydrofurans) **364**. Ruthenium-mediated ring closing metathesis yielded the expected aromatic, sugar- or beta lactam-based cyclophanes **365** (Scheme 63, reaction a).^268^

In a different approach, the tetracyclic indoline skeleton **368** was synthesized by a gold catalyzed cascade reaction, including a propargyl migration in aniline based compounds **366** to yield allenic indole intermediates **367**. Further cyclization and rearrangement provided the observed bridged indolines **368**. Challenging three-dimensional polycyclic skeletons are furnished, building three rings and four C–C bonds in one sole operational step (Scheme 63, reaction b).^269^

**Scheme 64:**
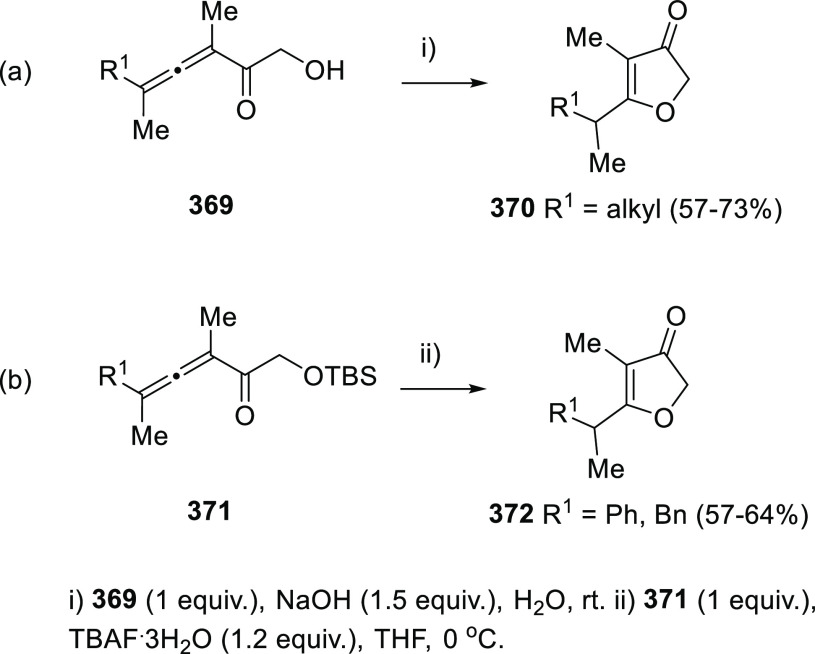
Metal-Free Conversion of Allenic Hydroxyketones into 3(*2H*)-Furanones

A metal-free oxycyclization of allenic hydroxyketones **369** in aqueous media has been reported. Inexpensive NaOH promotes the nucleophilic attack of the OH group toward the central allenic carbon through oxa-Michael-type reaction, yielding 3(*2H*)-furanones **370** with good yields (Scheme 64, reaction a). Moreover, cyclization may be achieved spontaneously by TBAF-mediated deprotection of silyl allenic ethers **371** (Scheme 64, reaction b). Noteworthy, gold catalysis failed promoting the oxycyclization of allenic hydroxyketones **372**, stablishing the reported metal-free strategy as an alternative for the most common catalytic procedures.^270^

**Scheme 65:**
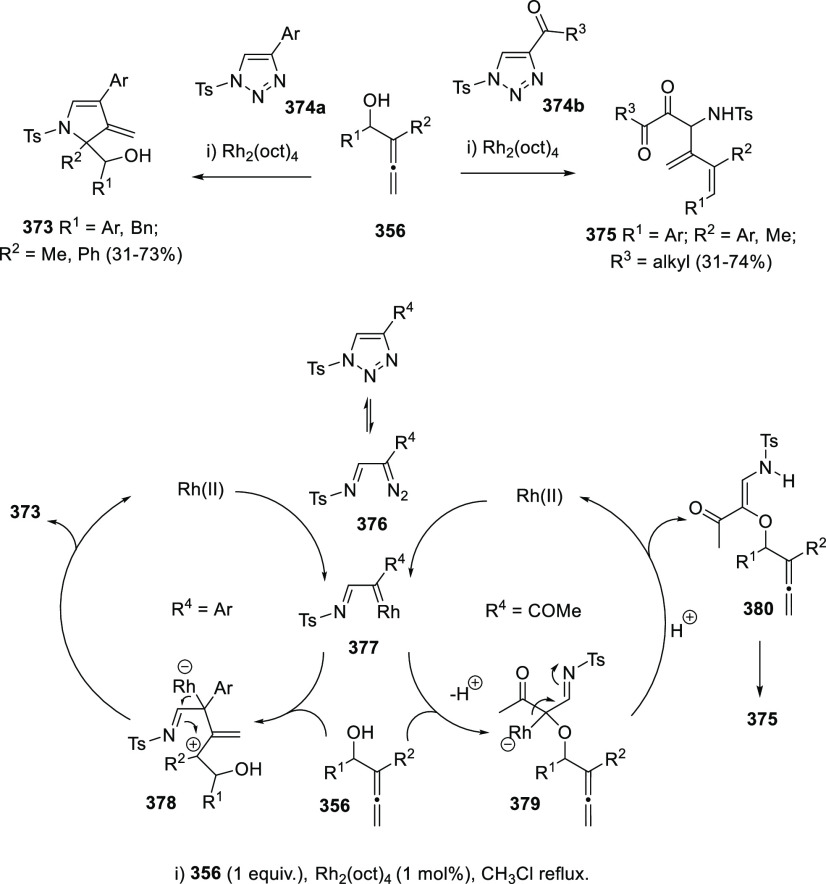
Rhodium-Catalyzed Nucleophilic Addition of Allenols to 4-Substituted-1-tosyl-1,2,3-triazoles

Despite that most of the transformations involving nucleophilic hydroxyls embedded in allenol moieties lead to cyclic final structures, mainly 5- or 6-membered oxacycles, some reports have appeared presenting the synthesis of open-chain products. Alcaide and Almendros research group have envisioned a change on the selectivity of the nucleophilic attack of allenols to rhodium carbenoids derived from triazoles, depending on the heterocycle substitution. Reaction of allenols **356** with 4-aryl-substituted triazoles **374a** yielded pirrolines **373**,^271^ while 4-acetyl-substituted triazoles **374b** provided diketones **375**, under otherwise identical reaction conditions (Scheme 65, top).^272^ Thus, when 1-tosyl-1,2,3-triazoles **374a** presented an aryl group in C4, allenols **356** behaved as *C*-nucleophiles, generating intermediates **378** as the product of the nucleophilic addition of the central allenic carbon onto the rhodium carbenoid in intermediates **377**. Further aza-cyclization of **378** yielded the experimentally observed pirrolines **373** (Scheme 65, bottom, left). Nonetheless, when 4-acetyl-1-tosyl-1,2,3-triazoles **374b** were employed, allenols **356** selectively behaved as *O*-nucleophiles, leading to species **379** as reaction intermediates. Then, regeneration of the ruthenium species and protonation would provide allenyl vinyl compounds **380**, which spontaneously evolve through a Claisen-type rearrangement to the observed final structures **375**, exhibiting both the interesting 1,2-diketone and *Z*-1,3-diene frameworks (Scheme 65, bottom, right).

**Scheme 66:**
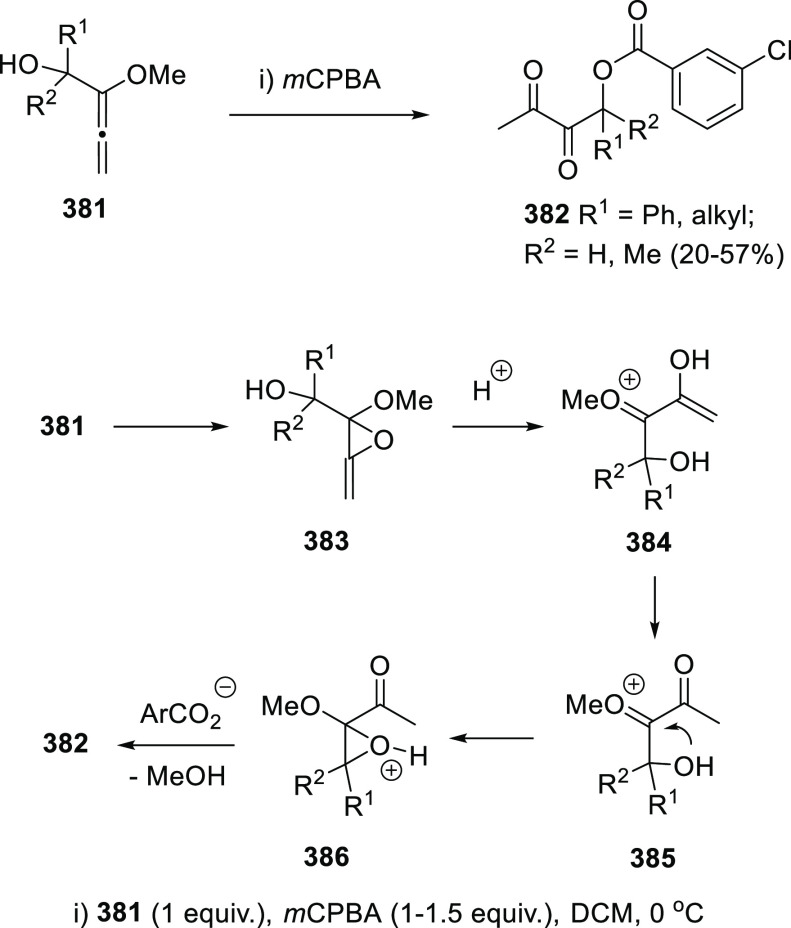
*m*CPBA-Mediated Synthesis of 1,2-Diketones from α-Hydroxyl Methoxyallenes

Zimmer, Reissing, and collaborators accessed the 1,2-diketone framework from α-allenols through a different pathway. Reaction of α-hydroxy methoxyallenes **381** with *m*CPBA yielded acyloxy-substituted 1,2-diketones **382** in reasonable yields. A mechanism rationale for this transformation would start from selective epoxidation of the proximal allenic double bond in **381** to generate intermediate **383**. Acid-promoted ring opening and tautomerization would then lead to dicarbonyl **385**, which may suffer intramolecular nucleophilic attack of the hydroxyl group of the former allenol moiety providing oxiranium **386**. Nucleophilic attack of 3-chlorobenzoate would promote the ring opening of cationic intermediates **386** generating the observed diketones **382** after loss of methanol (Scheme 66).^273^

**Scheme 67:**
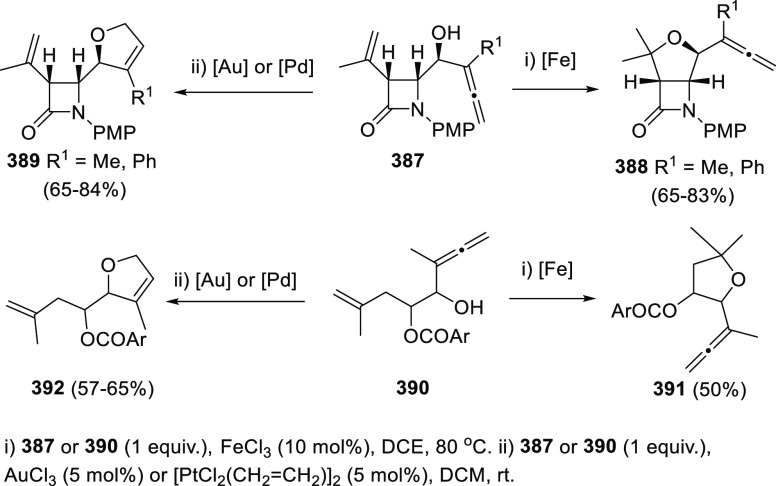
Allenol *vs* Alkenol Reactivity in Metal Catalyzed Reactions of Enallenols

Enallenols, multifunctional molecular targets bearing an alkene and allene moieties along with a hydroxyl group, have attracted recent interest due to their divergent and intricate reactivity. Alkenol *versus* allenol selective reactivity has been encountered in metal-catalyzed reactions of different enallenols such as β-lactam-based compounds **387** or acyclic derivatives **390**. Noteworthy, FeCl_3_ was found to exclusively provide alkenol oxycyclization adducts **388** and **391**, leading therefore to the tetrahydrofuran skeleton (Scheme 67, right). On the other hand, gold and platinum salts selectively catalyzed the allenol *5-endo-trig* oxycyclization of **387** and **390** generating the dihydrofuran motifs **389** and **392** in a chemoselective manner (Scheme 67, left).

**Scheme 68:**
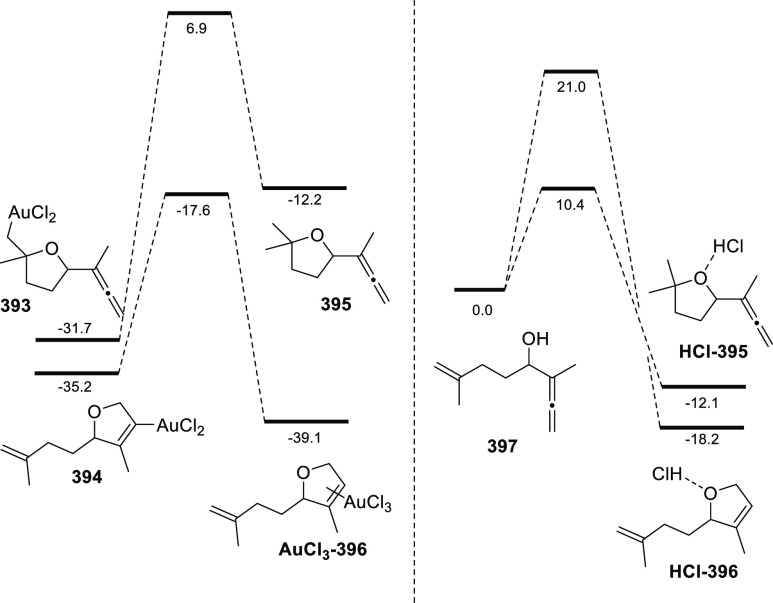
Computed Reaction Profile for Allenol *vs* Alkenol Cyclization of Enallenols Relative free energy data are given in kcal mol^–1^. HCl-mediated oxycyclization is taken as model reaction for the Lewis acid-FeCl_3_ alkenol oxycyclization reaction.

Density functional theory calculations showed coincident results according to the experimental observations. Thus, in both gold-based alkenol (reaction profile from **393**) and allenol (reaction profile from **394**) cyclizations, protodemetalation was identified as the bottleneck step of the whole process, finding a much lower energy barrier for the allenol cyclization resulting in the formation of dihydrofuran **396** (Scheme 68, left). Nevertheless, activation of the hydroxyl unit in substrates **397** should be the starting point of the catalytic cycle when iron salts are present, triggered by their strong Lewis acidity. In this case, alkenol cyclization pass by a notably lower energy barrier to yield the tetrahydrofuran skeleton **395** under kinetic control (Scheme 68, right).^274^

**Scheme 69:**
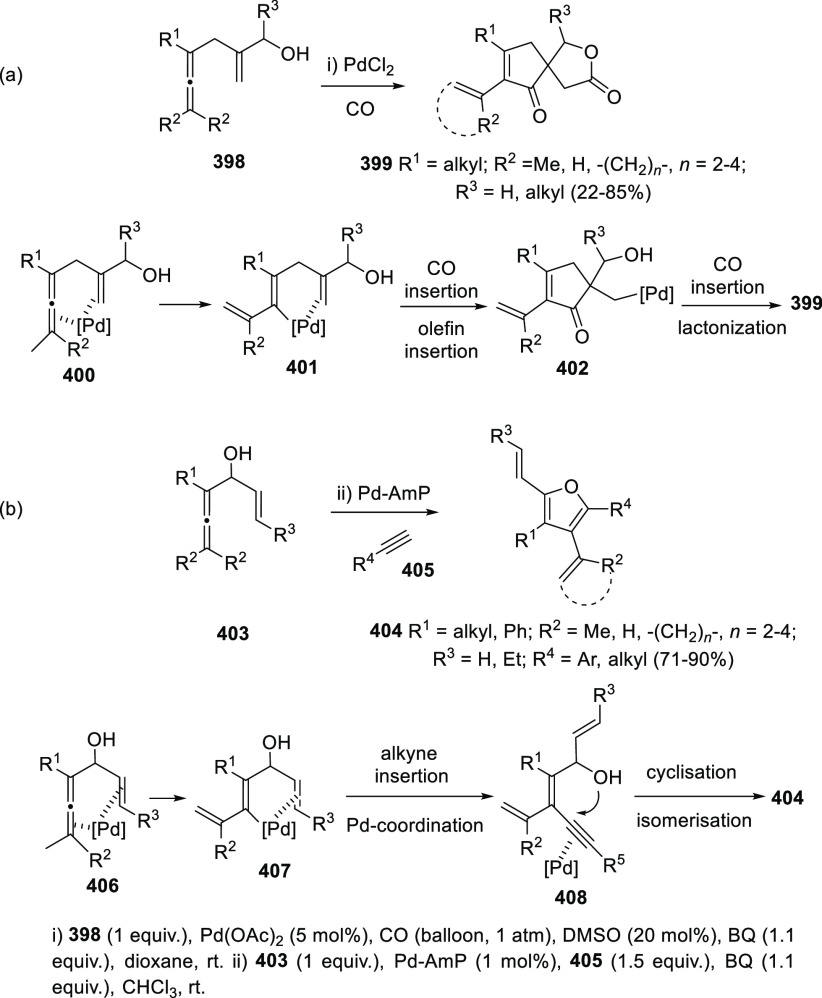
Palladium-Catalyzed Cascade Processes of Enallenols

Different reports on enallenol chemistry have stated the importance of designing the appropriate enallenol skeleton to modulate its reactivity. Bäckvall and co-workers have explored enallenol cyclization reactions under palladium catalysis in the presence of different cocyclization partners. In both substrates **398** and **403**, where the previously mentioned allenol *versus* alkenol competitive cyclization is not feasible, palladium catalyzed cocyclization reaction should start by dual coordination of the metal ion with both allene and alkene moieties (coordination intermediates **400** and **406**). Thus, when enallenols **398** were submitted to Pd(OAc)_2_ tretament under CO atmosphere, spirolactones **399** were obtained as sole reaction products in moderate to good yields. The process includes a cascade oxidative carbonylation-olefin insertion to form intermediates **402**, followed by a second CO insertion-lactonization sequence providing spirocycles **399** (Scheme 69a).^275^ Noteworthy, three C–C single bonds, one C–O bond, and an all-carbon quaternary center are generated in one single operational step. On the other hand, when enallenols **403** were treated with palladium catalysts in the presence of terminal alkynes **405** as cocyclization partners, substituted furans **404** were obtained with good to excellent yields. In this case, a heterogeneous palladium catalyst was employed, based on an aminopropyl-decorated siliceous mesocellular foam which hosts palladium nanoparticles, exhibiting great performance and high recyclability. Again, palladium insertion into the central allenic carbon would yield intermediate **407**, which may evolve through alkyne insertion to adduct **408**. Nucleophilic attack of the hydroxyl unit to the Pd-activated alkyne and further isomerization would furnish the observed furans **404** (Scheme 69b).^276^

**Scheme 70:**
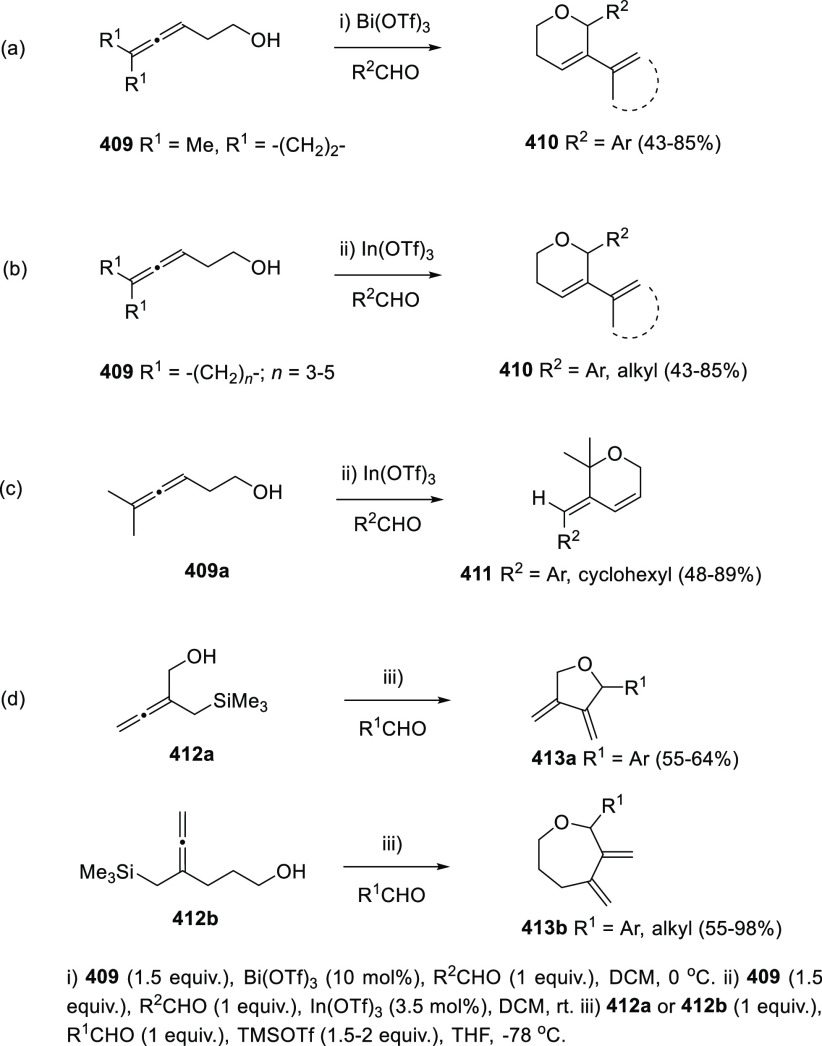
Prins-type Co-cyclization of Allenols and Aldehydes

Co-cyclization of allenols with aldehydes through Prins-type processes have also been reported, providing the synthesis of oxacycles with different ring sizes. β-Allenols **409** react with aromatic aldehydes in the presence of Bi(OTf)_3_ as Lewis acid catalyst to yield the dihydropyran skeleton **410** in moderate yields (Scheme 70, reaction a).^277^ When allenols **409** were treated with In(OTf)_3_ as Lewis acid catalyst, major efficiency in terms of catalyst loading toward the cocyclization adducts **410** was found (Scheme 70, reaction b).^278^ Interestingly, reaction of 5,5-dimethyl substituted β-allenol **409a** and aldehydes under In(OTf)_3_ catalysis exhibited a special behavior. After the expected Prins-type cocyclization of **409a** and the corresponding aldehydes, indium-mediated ring opening and further rearrangement took place, generating the alternative dihydropyran structures **411** in practical yields (Scheme 70, reaction c).^278^ In addition, tetrahydrofuran-based compounds **413a** were achieved by reaction of α-hydroxy allenylsilane **412a** with α,β-unsaturated aldehydes under acid catalysis. Also, larger ring sizes were accessible through this methodology in excellent yields, such as 3,4-dimethylidene oxepanes **413b** obtained from the reaction of γ-allenol **412b** using both aromatic and aliphatic aldehydes (Scheme 70, reaction d).^279^

**Scheme 71:**
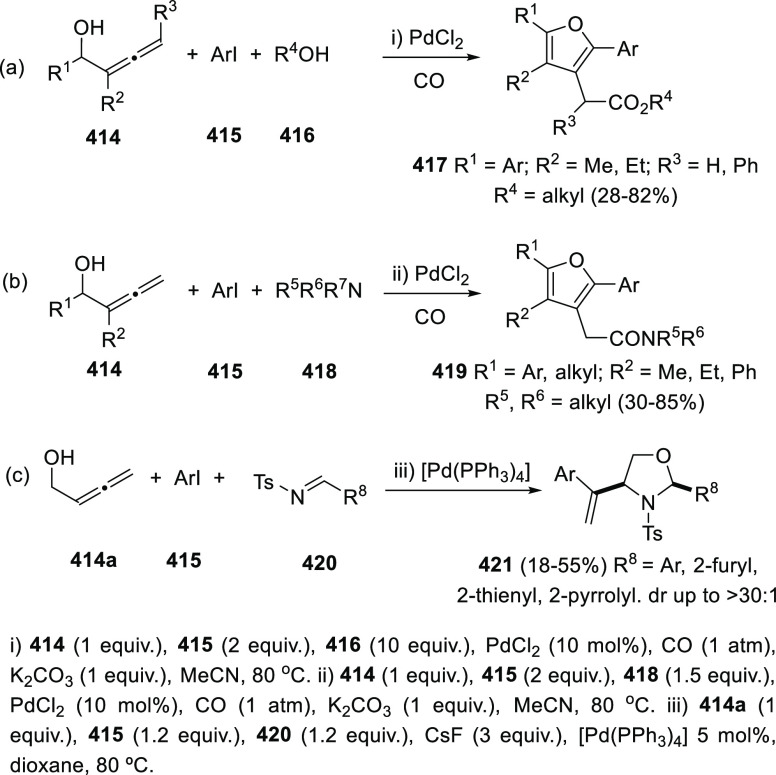
Palladium-Catalyzed Multicomponent Synthesis of Tetrasubstituted Furans and Oxazolidine Derivatives

One recurrent strategy to achieve the synthesis of poly substituted furans or dihydrofurans lays on the metal-catalyzed oxycyclization of α-allenols, followed by a cross-coupling process using diverse reagents such as aryl halides. Taking advantage of the low redox potential of palladium, many reports have appeared describing cascade processes of allenols promoted by palladium salts.^280−288^ Thus, a multicomponent reaction of α-allenols **414** with aryl iodides **415**, aliphatic alcohols **416** and carbon monoxide led to tetrasubstituted furans **417** through an oxidative addition/carbonylation reaction sequence (Scheme 71, reaction a).^289^ In a similar approach, the presence of tertiary amines **418** instead alcohols **416** in the reaction media provided the corresponding methylene acetamide-decorated furans **419** (Scheme 71, reaction b).^290^ Gong and collaborators have reported a related methodology using allenol **414a**, aryl iodides **415** and imines **420** as reaction partners also under palladium catalysis to provide an extense family of oxazolidine derivatives **421**. In this case, the proposed reaction mechanism includes carbopalladation onto the central carbon atom of the allene moiety, nucleophilic attack of the oxygen onto the C–C double bond of the imine, and latter ring closing step through nucleophilic addition of the nitrogen onto the inner π-allylic carbon atom (Scheme 71, reaction c).^291^

**Scheme 72:**
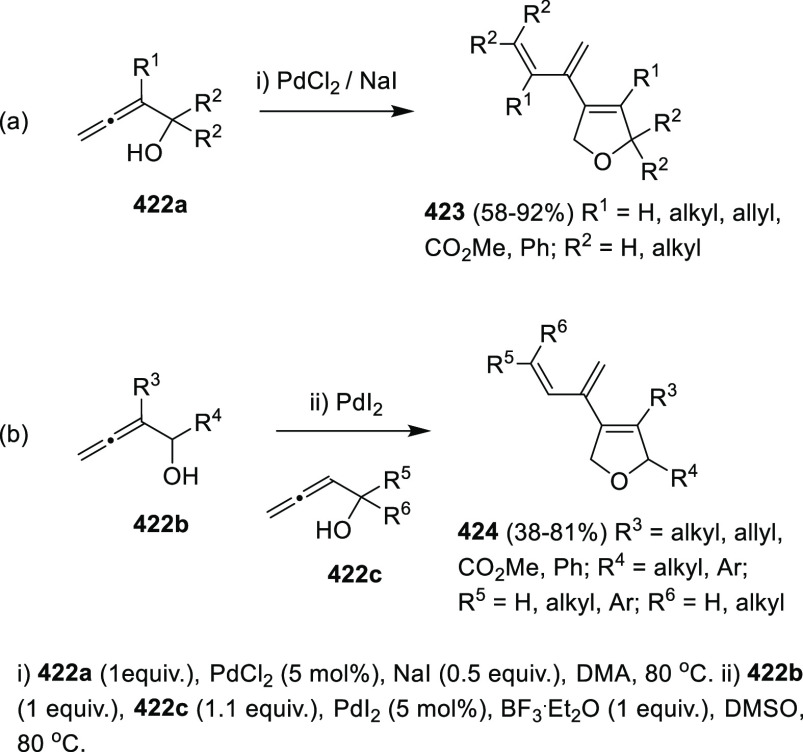
Palladium-Catalyzed Homo- And Heterodimeric Cross-Coupling Reaction of Allenols

Palladium species have also been found to be useful catalyzing homodimerization and heterodimerization processes of allenols. PdCl_2_ in the presence of NaI as additive has shown great activity promoting the tandem oxycyclization–cross-coupling reaction of 2-substituted allenols **422a**, yielding 4-(1′,3′-dien-2′-yl)-2,5-dihydrofurans **423** (Scheme 72, reaction a).^292^ Interestingly, the reaction of two different allenic species under similar reaction conditions allowed the synthesis of substituted dihydrofurans **424**, as a result of the chemoselective oxycyclization of 2-substituted allenols **422b**, followed by cross-coupling reaction with 2-unsubstituted allenols **422c** (Scheme 72, reaction b).^293^

**Scheme 73:**
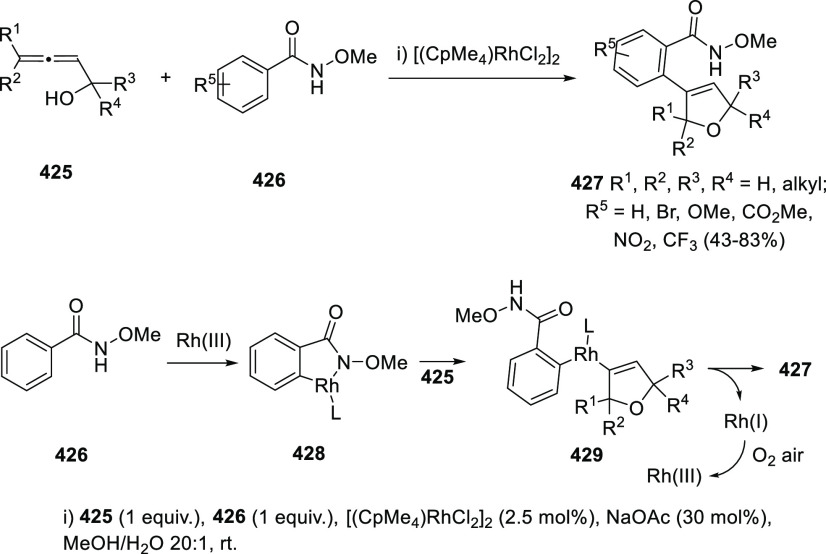
Rhodium-Catalyzed Oxycyclization/Cross-Coupling Reaction of α-Allenols and *N*-Methoxybenzamides

Besides the classical palladium-cross-coupling strategies, the past decade has started to witness the use of different metals to improve the efficiency and expand the scope of the methodology. Rhodium catalysis has been used to promote an oxycyclization–cross-coupling reaction of allenols with diverse benzamides including a challenging arene C–H bond insertion. Reaction of α-allenols **425** with Rh(III) species in the presence of *N*-methoxybenzamides **426** smoothly generated substituted dihydrofurans **427** with moderate to good yields. Notably, a wide substitution pattern on the allene moiety is tolerated, including sterically hindered substrates and tertiary alcohols. The most plausible reaction pathway would start by rhodation of benzamides **426** through the C–H bond, facilitated by coordination with the amide unit in complex **428**. Further coordination with the allene moiety of **425** and oxyrhodation would afford intermediate **429**, which could easily undergo reductive elimination to yield dihydrofurans **427** and liberate Rh(I) species. Atmospheric oxygen was used to reoxidize the catalytic species, returning the Rh(III) to the cycle (Scheme 73).^294^ A mechanistically different arene functionalization dealing with the intermolecular cyclization reaction of β-allenols in the presence of indoles catalyzed by a platinum salt, has been reported to afford C3-substituted indole derivatives with a tetrahydro-2*H*-pyran ring.^295^

**Scheme 74:**
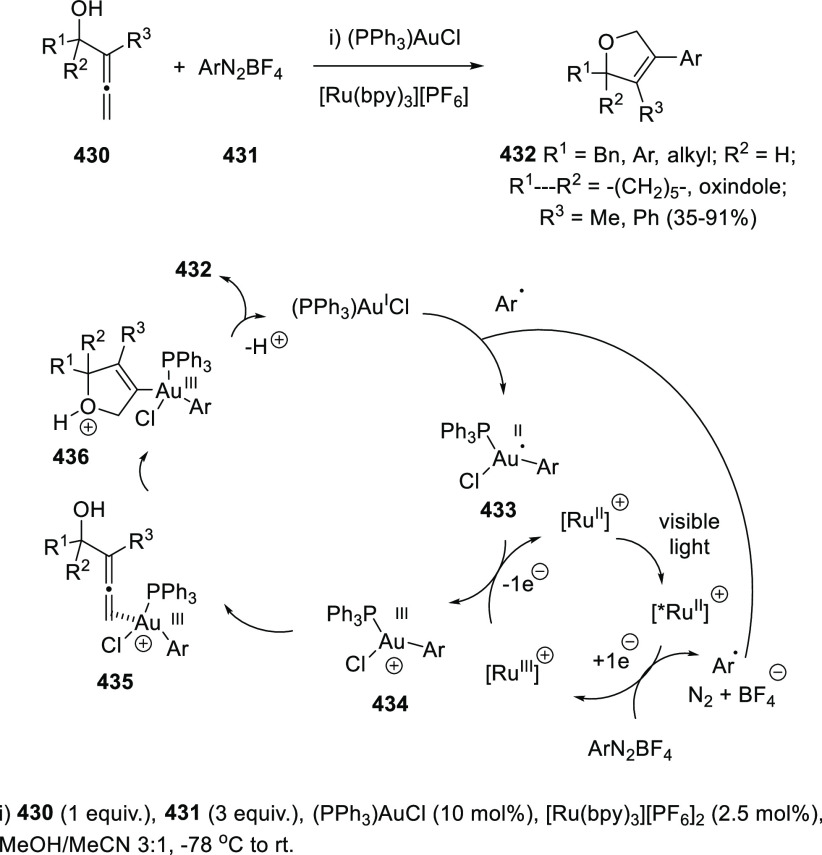
Gold-Catalyzed Photoredox Cross-Coupling of α-Allenols and Diazonium Salts

Opposite to palladium or rhodium species, the high redox potential of gold makes the oxidative addition/reductive elimination steps hard to perform on gold-mediated transformations, and consequently inadequate for cross-coupling reactions. On the other hand, the well-known ability of gold salts to catalyze allenol oxycyclizations, frequently the first step in cross-coupling processes, has prompted different research groups to find solutions to circumvent this problem. Thus, photoredox catalysis has been successfully applied to achieve a gold-mediated cross-coupling reaction of allenols and diazonium salts. Allenols **430** reacted with aryldiazonium salts **431** in the presence of AuClPPh_3_ and [Ru(bpy)_3_][PF_6_]_2_ as photoactive catalyst under visible light. The reaction provided a wide family of 2,3,4-trisubstituted dihydrofurans **432** in a regioselective manner (Scheme 74). Yields were moderate to excellent, finding the best results when deactivated aryldiazonium salts **431** were employed. In addition, diverse functionalities were well tolerated, such as CF_3_, Br or OMe. A mechanistic proposal may start from oxidative arylation of the gold species promoted by single electron transfer from photoactivated ruthenium complex, generating Au(III) species **434**. Coordination of **434** onto the allenic moiety of **430** resulted in complex **435**, which would induce the oxycyclization step, generating intermediate **436**. Reductive elimination would then recover the Au(I) species to the catalytic cycle and explain the formation of the observed 4-aryl-dihydrofurans **432** after deprotonation.^296,297^

**Scheme 75:**
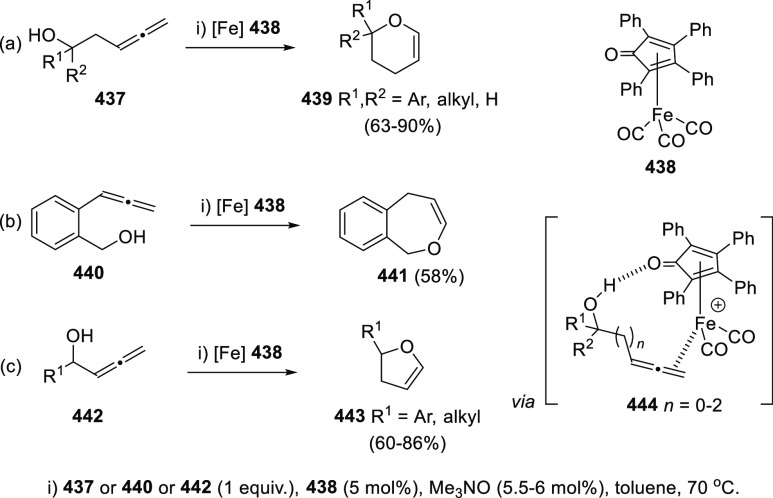
Metal–Ligand Cooperative Catalysis in Cycloetherification of α-, β-, and γ-Allenols

The reactivity of allenols has also been used in catalysis as a platform for testing the versatility of recently developed metal complexes. The allenol oxycyclization process has been applied as a model reaction to investigate new catalytic pathways along with the design and tuning of novel catalysts. In this context, Rueping and collaborators have informed of a rare metal–ligand dual catalysis for the cycloisomerization of β-allenols. Allenes **437** bearing a β-hydroxyl unit reacted with catalytic amounts of iron cyclopentadienone complex **438**, yielding 3,4-dihydro-2*H*-pyrans **439**, through a selective *6-endo-trig* oxycyclization/double bond isomerization process (Scheme 75, reaction a). The methodology was extended to both aromatic and aliphatic substituted allenols, providing the pyran skeleton in good yields. In addition, benzoxepine structure **441** was also accessible under similar reaction conditions through a more challenging *7-endo-trig* heterocyclization (Scheme 75, reaction b).^298^ The cooperative metal–ligand catalysis strategy was further applied to the synthesis of dihydrofuran systems **443** through a *5-endo-trig* cycloetherification of α-allenols **442** (Scheme 75, reaction c).^299^ Experimental and computational investigations revealed the role of the cyclopentadienone ligand as proton scavenger in a doubly activated intermediate complex **444**. Also, it is proposed to act as proton shuttle/proton acceptor facilitating the 1,2-H shift to overcome the eventual double bond isomerization.

**Scheme 76:**
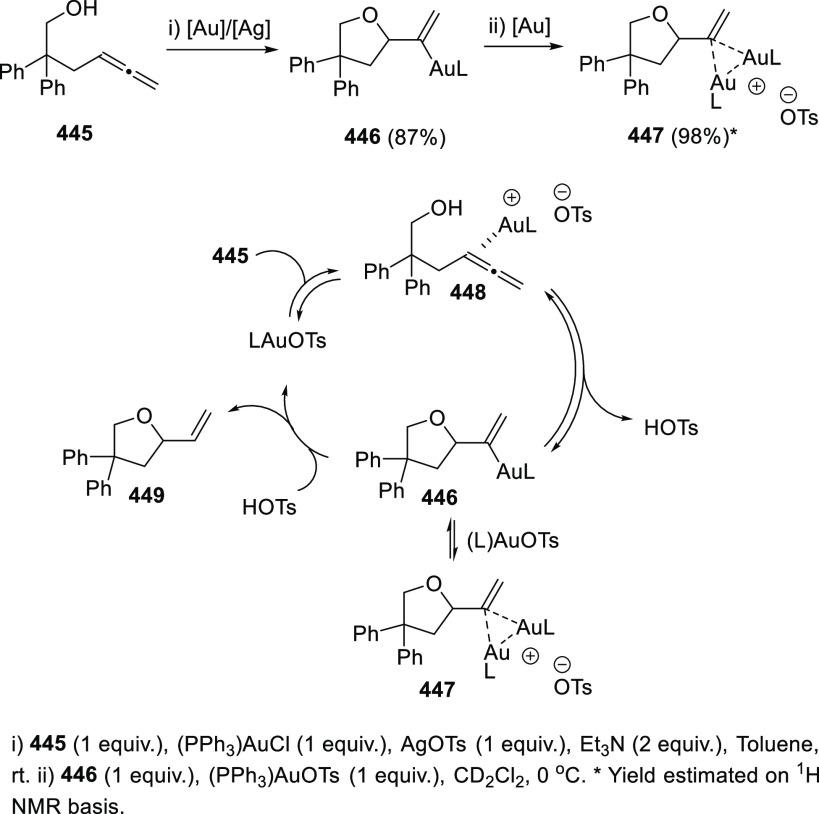
Mechanistic Pathway for the Gold-Catalyzed Hydroalkoxylation of Allenols

Widenhoefer and co-workers have selected the oxycyclization of 2,2-diphenyl-4,5-hexadien-1-ol (**445**) furnishing vinyl tetrahydrofuran **449** to perform the first mechanistic investigation on gold-catalyzed hydroalkoxylations. Experimental observations gave light to two major significant conclusions: (i) reversibility across the C–O bond formation step, and (ii) the presence of outer sphere bis(gold) vinyl species **447** acting as catalyst reservoir. Intermediate **447** was characterized from in-solution samples after treatment of stable vinyl gold complex **446** with (L)AuOTs at low temperature (Scheme 76, top). Then NMR monitoring experiments of the catalytic hydroalkoxylation of allenols **445** indicated the uninterrupted presence of bis(gold) vinyl species **447** during the whole reaction time. According to the results obtained from kinetic and deuteration experiments, the proposed catalytic cycle would start with a reversible C–O bond formation to generate mono(gold) vinyl intermediate **446** and HOTs. Aggregation should take place at this point to produce bis(gold) intermediate **447** in an outer-sphere equilibrium with mono(gold) species **446**. Also, kinetic experiments pointed the protodeauration of intermediate **446** as the rate-determining step, excluding a possible disproportionation of bis(gold) vinyl intermediate **447** as an alternative to yield the final tetrahydrofuran **449** (Scheme 76, bottom).^300^

**Scheme 77:**
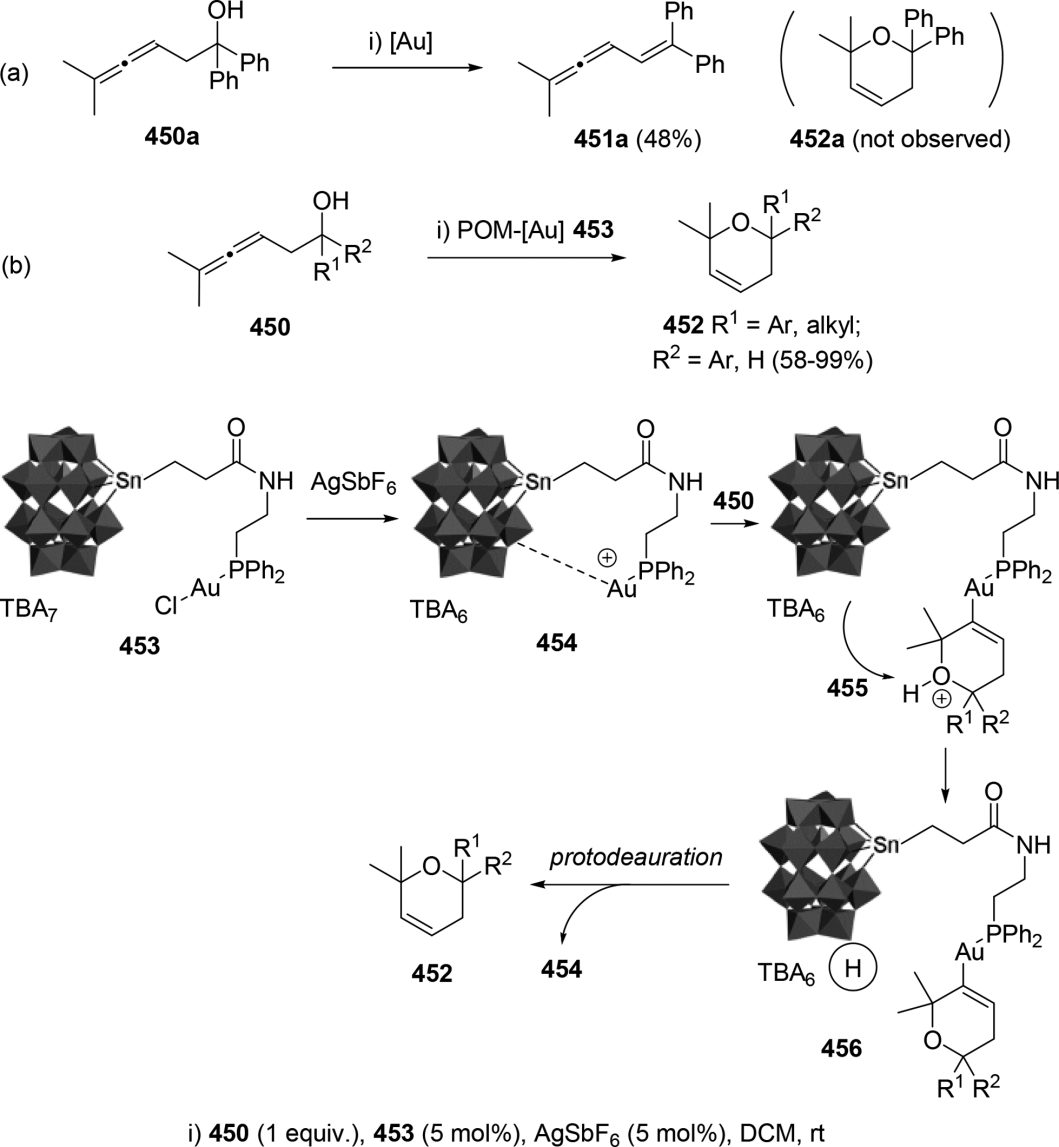
POM-Au Catalyzed Hydroalkoxylation of Acid-Sensitive Allenols

Related bis-benzylic β-allenol **450a** undergoes elimination in the presence of the major part of gold catalysts to yield conjugated vinyl allenes **451a** instead of the oxycyclization product **452a** (Scheme 77, reaction a). Same result has been observed on similar acid-sensitive allenols **450**, due to the acidic nature of gold salts and complexes. Lacôte et al. have envisioned a gold self-buffering catalytic system to solve this problem and achieve the cycloetherification products **452** in substrates were dehydratation competes. Polyoxometalate (POM)-based catalyst **453** were designed and synthesized, using organotin-substituted polyoxotungstate [P_2_W_17_O_16_{Sn-(CH_2_)_2_–CO}]^6–^ as POM surface and ω-amino gold phosphine complexes to provide the active metal site. Thus, acid-sensitive allenols **450** were treated with a catalytic amount of **453** and AgSbF_6_ to induce the activation of the gold specie as cationic gold. Conversions of allenols **450** into dihydropyran structures **452** were complete in all cases, with moderate to excellent yields and no elimination byproducts identified (Scheme 77, reaction b). Interestingly, mechanistic insights revealed a multiple role from the POM-Au catalyst: Coordination of the cationic gold to the surface may stabilize the intermediate **454**, reducing catalytic activity at the same time it improves catalyst recyclability. The POM surface should be also acting as proton catcher, buffering the reaction media and facilitating the formation of intermediate **456**. Nevertheless, eventual protodeauration step to yield final adducts **452** would need a disfavored proton release from the POM skeleton, explaining the low reaction rates (up to 5 days to completion) compared to regular homogeneous gold-catalyzed hydroalkoxylations. Finally, special solubility of POM systems provides catalyst recycling, constituting this approach as a greener and more economic strategy.^301^

**Scheme 78:**
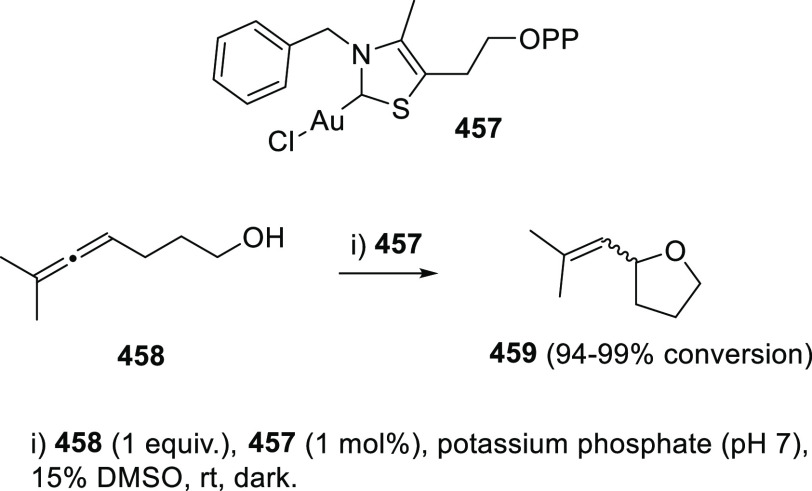
Thiazolium Gold(I) Carbene-Catalyzed Hydroalkoxylation of Allenols

During the past decade, different contributions on gold catalysis have appeared describing both a rationale design as well as synthesis and applications of novel catalysts that could be able to provide higher efficiency and greener procedures. Noteworthy, the hydroalkoxylation of allenols has been frequently chosen as model reaction. Hilvert’s research group has described the synthesis of thiazolium gold(I) carbenes **457**, as a greener alternative to the well-known imidazolium analogous. The higher hydrophilicity of thiamine units, together with the presence of a pyrophosphate group, improves the catalyst stability, and allows the use of aqueous media in hydroalkoxylation reactions. Thus, γ-allenol **458** could be successfully transformed (up to 98% conversion) into tetrahydrofuran **459** under mild reaction conditions and open-air experiments (Scheme 78).^302^

**Scheme 79:**
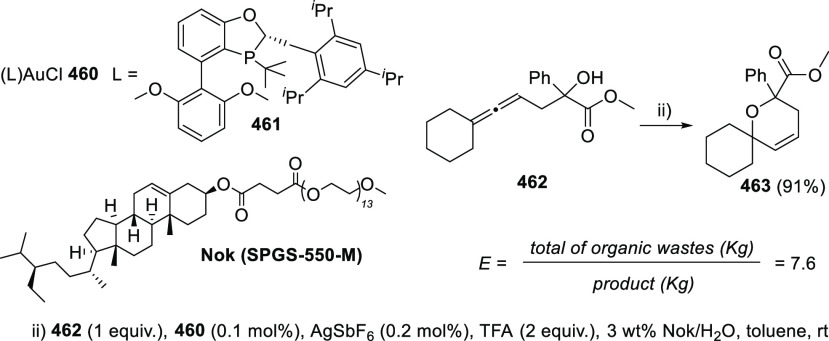
Micellar-Supported Gold(I) Catalyzed Hydroalkoxylation of Allenols

In the pursuit of more economic and environmentally friendly strategies including precious metal catalysis, Lipshutz and co-workers have recently reported the use of gold(I) salts in micellar systems. Surfactant Nok (SPGS-550-M) was employed in combination with the newly synthesized gold(I) salt **460**, showing an improved lipophilicity (Scheme 79). The micellar cavities based on aggregation of Nok molecules in aqueous media behaved as organic-based nanoreactors, encapsulating the hydrophobic reagents and therefore increasing their effective concentration. Thus, both uses of solvent and catalyst could be minimized, resulting in high conversions with catalyst loadings of 0.1 mol %. Also, an E factor of 7.6 for the oxycyclization of allenols **462** into spirocyclic systems **463** indicates the promising greener advantages of the micellar-based strategy.^303^

**Scheme 80:**
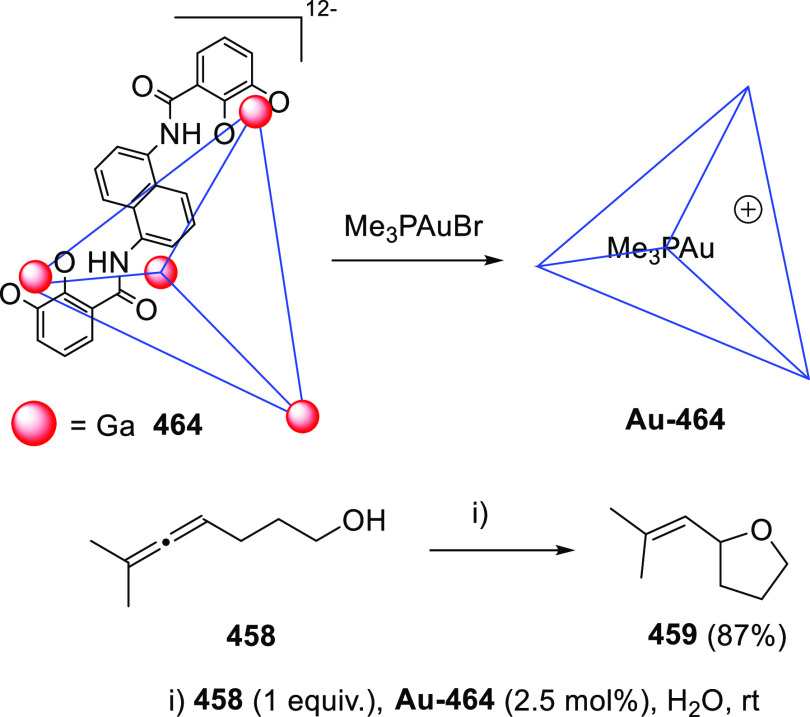
Supramolecular Gold GaL_6_-Hosted Catalyst and Its Application to Hydroalkoxylation Reaction

Bergman, Raymond, Toste, and collaborators have introduced supramolecular chemistry in gold catalysis from a different perspective. Gallium-based tetrahedral macromolecule **464** has been used as a supramolecular host for cationic gold species, acting as an enzyme-mimic catalyst. Again, hydroalkoxylation of allenol **458** has been selected as model transformation, to study the catalytic activity of Au-**464** species (Scheme 80). Noteworthy, encapsulation of the gold salt induces ionic bond dissociation, resulting in more active “naked” cationic gold species. Thus, the catalytic activity is 8-fold increased compared to regular homogeneous cationic gold procedures, and up to 67 catalytic turnovers were observed.^304^

**Scheme 81:**
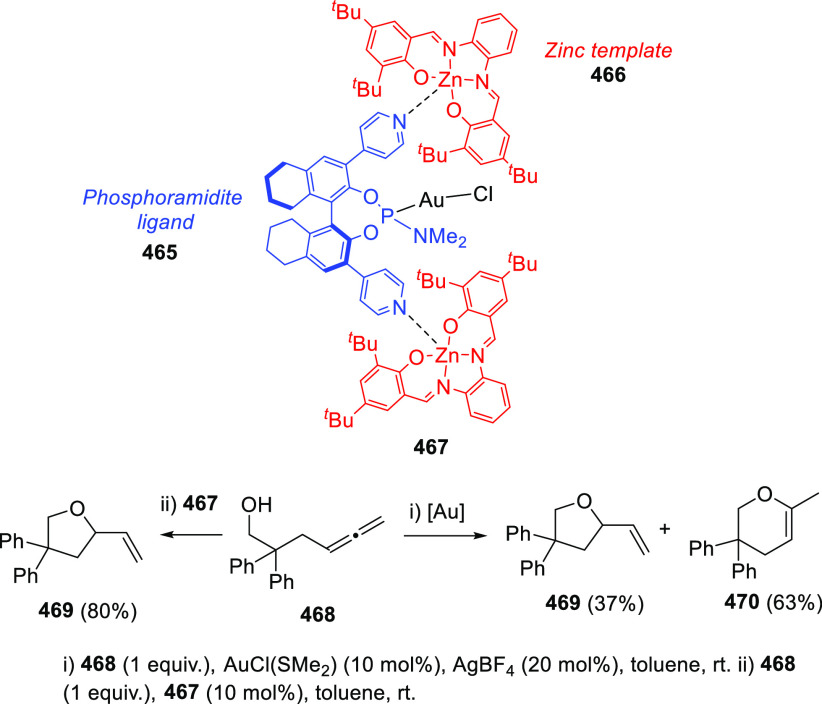
Supramolecular Gold(I) Catalysis in Selective Oxycyclization of γ-Allenols

Following a similar concept, Reek et al. have envisioned a gold(I) system bearing supramolecular ligands, as inductor of selectivity in the oxycyclization reaction of allenols. Pyridyl-decorated phosphoramidite ligands **465** were used to both bonding to the active gold ion and to link Zn template **466** through the pyridyl nitrogen atom, generating the supramolecular structure **467**. Although lower conversions were achieved compared to AuCl(L) complexes under similar reaction conditions, complete selectivity toward *5-exo-trig* cyclization of allenol **468** was observed, yielding vinyl tetrahydrofuran **469** as sole reaction product (Scheme 81).^305^

### 3.3 Allenols as Allenes Showing an Extra Coordination Site

Besides the allenol transformations where the hydroxyl group behaves as a leaving group or as a nucleophile, seen in previous sections 3.1 and 3.2 respectively, the recent literature has also provided several examples of alternative hydroxyl-assisted reactions. This third class of allenol reactivity includes both metal-catalyzed processes where the M–O coordination is crucial for a specific transformation as well as bond migration reactions promoted by the hydroxyl lone electron pairs. In those cases, the hydroxyl unit is retained unaltered in the final products or oxidized into a carbonyl group.

Araki and co-workers reported one early example of OH-assisted allylindation of allenols. Hydroxyl-chelated bicyclic species are reported as the most plausible transition states for this transformation.^306^

**Scheme 82:**
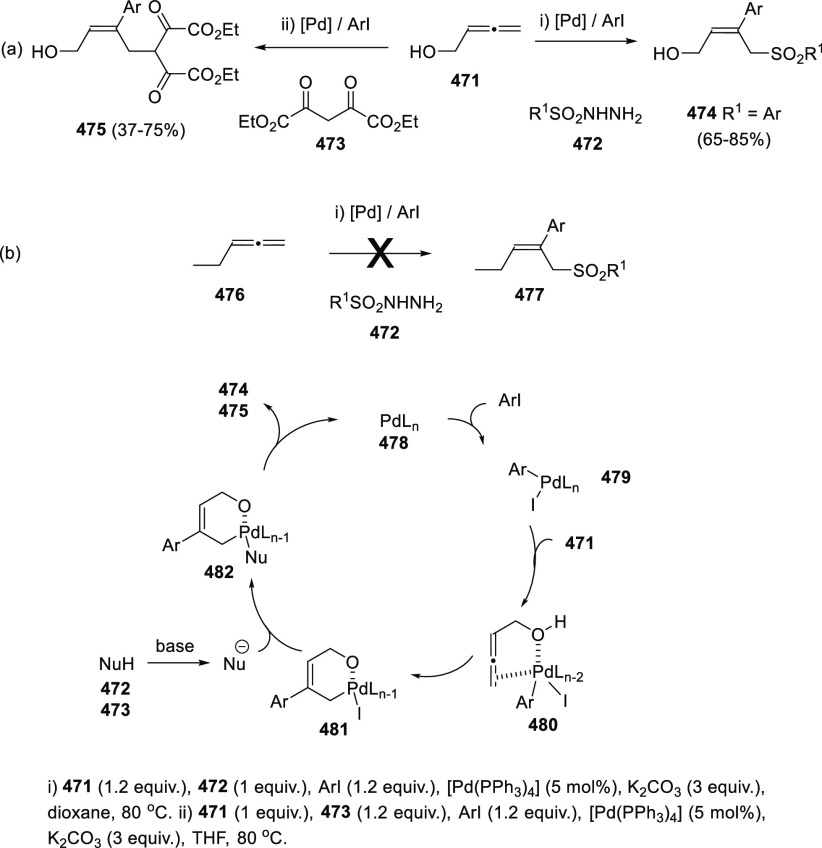
Pd-Mediated Synthesis of Allylic Alcohols

Gong and collaborators have reported a three-component methodology for the synthesis of 3,3′-disubstituted allylic alcohols under palladium catalysis. Treatment of allenic alcohol **471** with aryl iodides, catalytic amounts of a Pd(0) complex and the adequate pro-nucleophile **472** or **473**, provided allylic alcohols **474** and **475** with moderate to good yields and complete *Z*-selectivity (Scheme 82, reaction a). The hydroxyl group in the allenol skeleton **471** is proposed to perform a double role: (i) enhancement of the reactivity by palladium-coordination in metallacycle intermediate **481**, and (ii) both regio- and stereodirection for the addition of the aryl and nucleophile moieties. A mechanistic proposal for this transformation would start with the oxidative addition of the Pd(0) catalyst to the corresponding aryl iodide to generate Pd(II) intermediate **479**. Coordination and carbopalladation would furnish the above-mentioned cyclic intermediate **481**. Reaction with the *in situ* generated nucleophile followed by reductive elimination could lead to the observed allylic alcohols **474** or **475** (Scheme 81, bottom). Interestingly, allene **476** lacking hydroxyl unit, failed to yield the corresponding substituted alkene **477**, supporting the proposed mechanistic pathway and the coordinative role of the OH group in the reported transformation (Scheme 82, reaction b).^307−309^

**Scheme 83:**
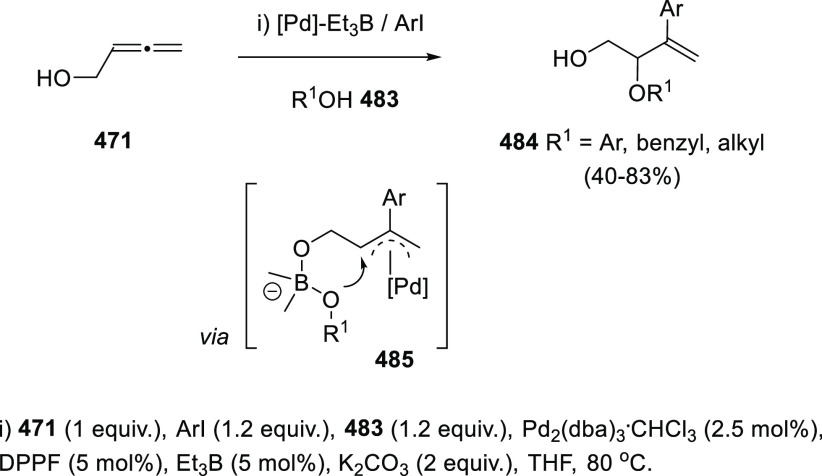
Cooperative Pd/B-Catalyzed Transformation of Buta-2,3-dien-1-ol into Allylic Alcohols

Zhang’s research group has reported a three-component palladium-promoted variant for the synthesis of allylic alcohols from allenol **471**, aryl iodides, and alcohols as *O*-nucleophiles. In this case, a cooperative borane-palladium catalyzed strategy was developed. Triethyl borane was used as coordinating agent, directing the addition of alcohols **483** toward the inner allenic carbon through intermediate **485**. The methodology was extended to a wide variety of substituted aryl iodides and both aromatic and aliphatic alcohols **483**, providing allylic alcohols **484** in moderate to good yields (Scheme 83).^310^

**Scheme 84:**
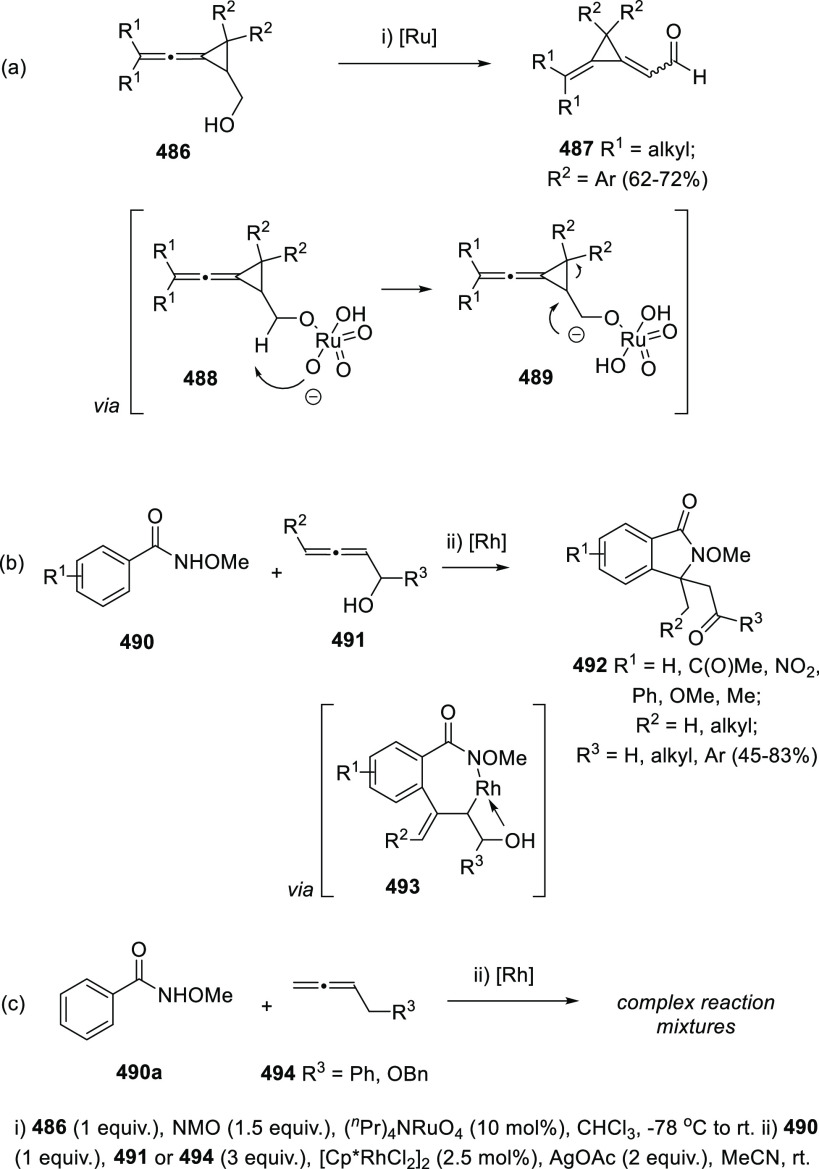
Allenol Transformations Promoted by Previous Metal-Hydroxyl Previous Coordination

Recent examples on allenol reactions triggered by metal–oxygen coordination includes Shi’s contribution on Ru-catalyzed oxidative isomerization of vinylidene cyclopropanes **486** to aldehydes **487** trough intermediate **488** (Scheme 83, reaction a).^311^ Also, Lu and collaborators have reported the reaction of aromatic amides **490** and allenols **491** in the presence of catalytic amounts of Rh(III) to yield γ-lactams **492** (Scheme 84, reaction b). The authors stated the significant role of the hydroxyl group in controlling both the regio- and the stereochemical outcome, probably through coordination with the rhodium atom in intermediate **493**. A control experiment from allene **494** lacking hydroxyl group, which lead to complex reaction mixtures supported the Rh–O coordination hypothesis (Scheme 84, reaction c).^312^

**Scheme 85:**
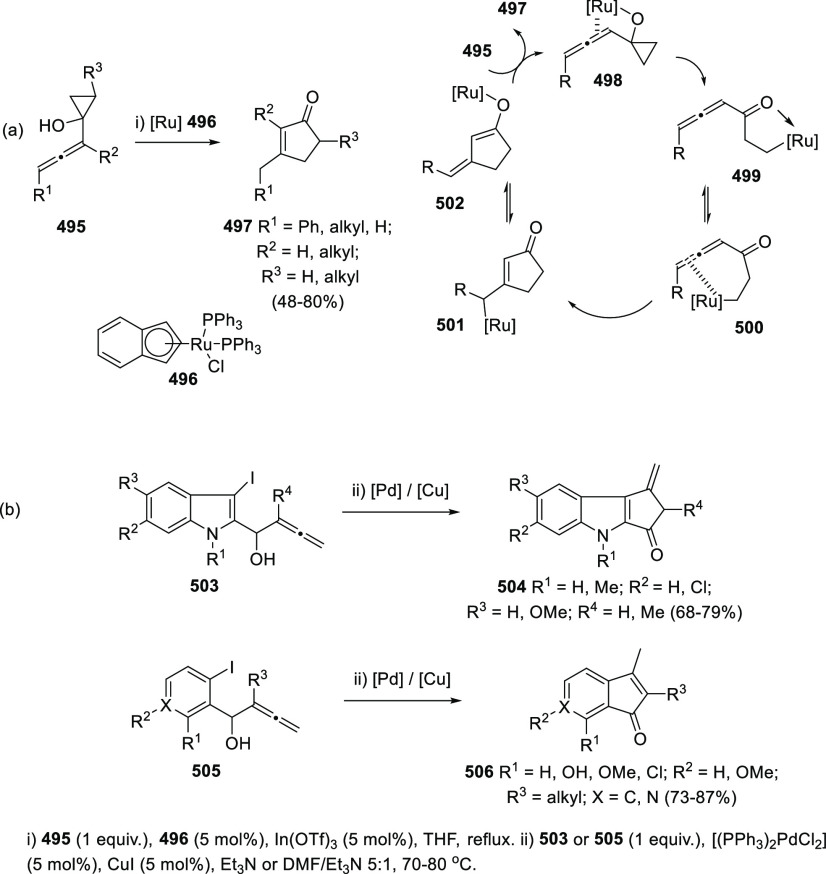
Synthesis of Cyclopentenones by Metal-Catalyzed Rearrangements of Allenols

The cyclopentenone skeleton is a recurring target in organic synthesis, exhibiting an extense range of biological activities and synthetic applications. Besides the well-known Pauson-Khand and Nazarov cyclizations, metal-catalyzed cycloisomerizations have recently appeared as synthetic strategies to achieve the cyclopentenone motif.^313−316^ In this context, Cha and co-workers have described an allenol-based ring expansion process involving a ruthenium–oxygen coordination to provide the cyclopentenone system. Thus, allenyl cyclopropanols **495** were treated with ruthenium complex **496** and In(OTf)_3_ as additive, generating cyclopentenones **497** in moderate to good yields (Scheme 85, reaction a, left). The mechanistic pathway may start from dual coordination of the metal in alcoholate **498**. Ring opening of the strained three-membered ring would then provide intermediate **499**, which might evolve through a migratory insertion to cyclic intermediate **501**. Eventual ligand exchange would explain the observed cyclopentenones **497** and return the active metal species to the cycle (Scheme 85, reaction a, right).^317,318^ Alcaide and Almendros research group has also contributed to the allenol/cyclopentenone transformation in the context of a cooperative bimetallic catalysis. In this case, 2-iodoaryl allenols **503** and **505** were treated with [(PPh_3_)_2_PdCl_2_] and CuI as bimetallic pair, yielding differently substituted fused cyclopentenones **504** and **506** in good yields (Scheme 85, reaction b), through a proposed intramolecular Heck-type coupling reaction mechanism.^319^

**Scheme 86:**
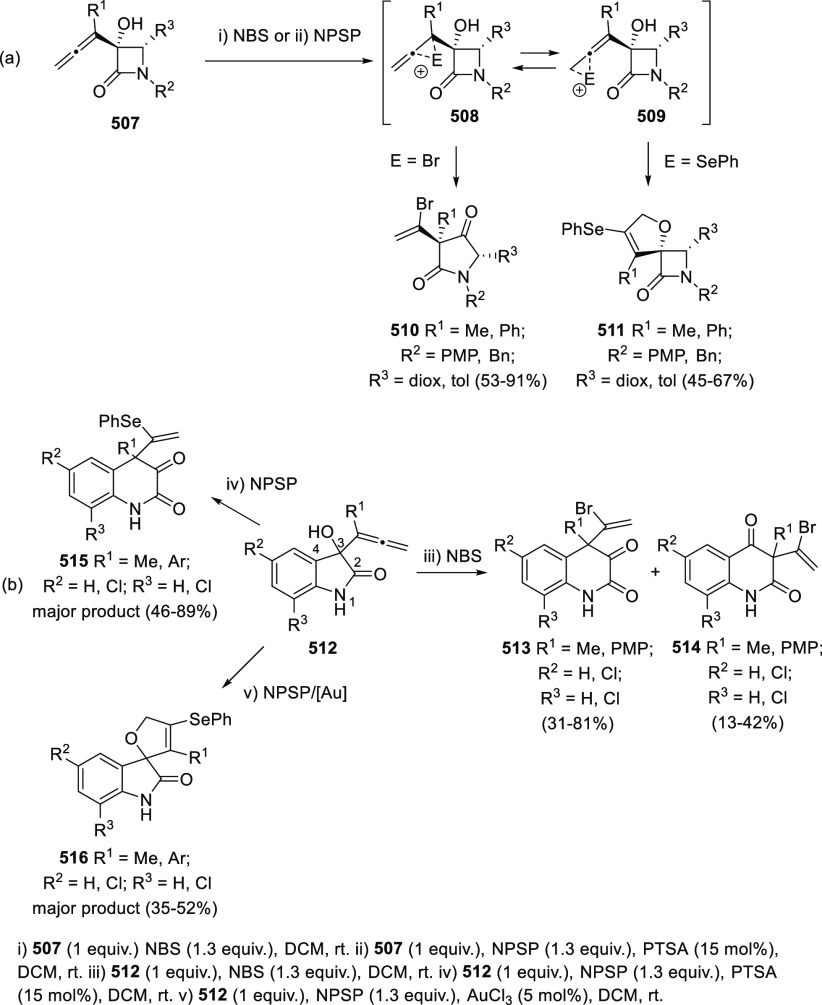
Halogen- and Selenium-Mediated Ring Expansion Reactions of Allenols

Hydroxyl-assisted bond migrations in allenol systems have been frequently reported by different research groups. Ma and collaborators have pioneered halogen-promoted 1,2-aryl shift, and 1,2-H shift in allenol skeletons.^320,321^ Thus, secondary and tertiary allenols were reported to smoothly generate 3-halo-3-alkenals and 2-halo-2-alkenyl ketones respectively, under halogenating reagents such as Br_2_, I_2_, NIS, or NBS. During the past decade, related halogen-promoted 1,2-bond migrations in allenol systems have been employed in ring expansion processes. Moreover, selenating reagents were found to induce an intriguing selectivity on this transformation. Thus, 2-azetidinone-tethered allenols **507** were reported to undergo a selective 1,2 C–C bond migration in the presence of NBS, providing tetramic acids **510** (Scheme 86, reaction a). In contratst, the use of *N*-phenylselenophthalimide as electrophile promoted the oxycyclization process yielding spirocyclic seleno-β-lactams **511**, under otherwise similar reaction conditions.^322^ Species **508** and **509**, formed by coordination of the electrophile to the proximal and distal allene double bond respectively, are proposed as raisonable intermediates for the divergent transformation (Scheme 86, reaction a). In addition, 2-indolinone-tethered allenols **512** under NBS conditions provided the corresponding quinolone skeletons **513** and **514** through a related ring expansion process (Scheme 86, reaction b, right).^323^ Noteworthy, mixtures of two regioisomers were frequently found, quinolone-2,3-diones **513** as major products from a C3–C4 bond cleavage, together with quinoline-2,4-diones **514** from a less favorable C2–C3 bond breakage. Interestingly, selenating reagents improved the divergency of the process, finding selenoquinoline-2,3-diones **515** as major or sole reaction products in the presence of *N*-phenylselenophthalimide (NPSP) (Scheme 86, reaction b, top left). Spirocyclic selenolactams **516** where achieved when phenylselenyl bromide was used. Also, AuCl_3_–NPSP cocatalyzed reaction of allenols **512** favored the formation of the spirocyclic products **516**, probably due to the gold ability in promoting oxycyclization transformations in allenols (Scheme 86, reaction b, bottom left).^324^

**Scheme 87:**
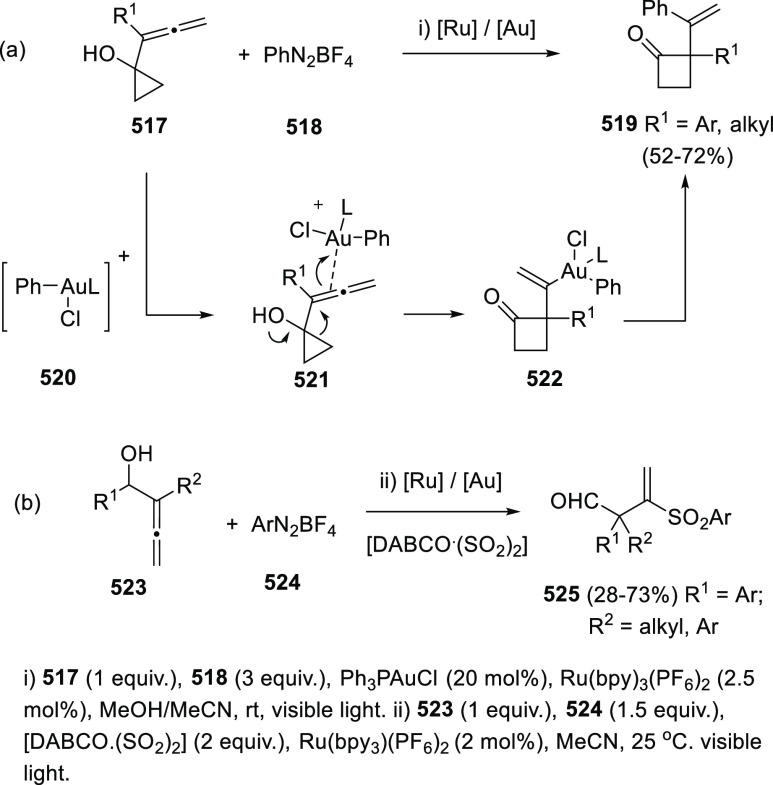
Dual Gold-Photoredox Catalyzed Arylative Rearrengement of Allenols

Toste’s research group has presented a photoredox-catalyzed ring expansion methodology in ciclopropane-linked allenol systems. Compounds **517** undergo ring expansion and oxidative arylation processes in the presence of electrophilic gold(III)-aryl complex **520** which is generated *in situ* from benzenediazonium salt **518**. Coordination of the gold complex to the proximal allenic double bond in allenols **517** to give intermdediates **521** would promote the oxidative ring expansion process toward intermediates **522**. The observed four-membered cyclic ketones **519** are obtained in practical yields from reductive elimination of intermediates **522** (Scheme 87).^325^ On the same basis of photoredox catalysis, Almendros and Luna et al. have recently reported the synthesis of 3-(arylsulfonyl)but-3-enals **525** from allenols **523**, sulfur dioxide, and arenediazonium salts **524** under visible light. The proposed mechanistic pathway includes a C–C bond migration facilitated by the latter oxidation of the hydroxyl group to the corresponding aldehyde in compounds **525** (Scheme 87, reaction b).^326^

**Scheme 88:**
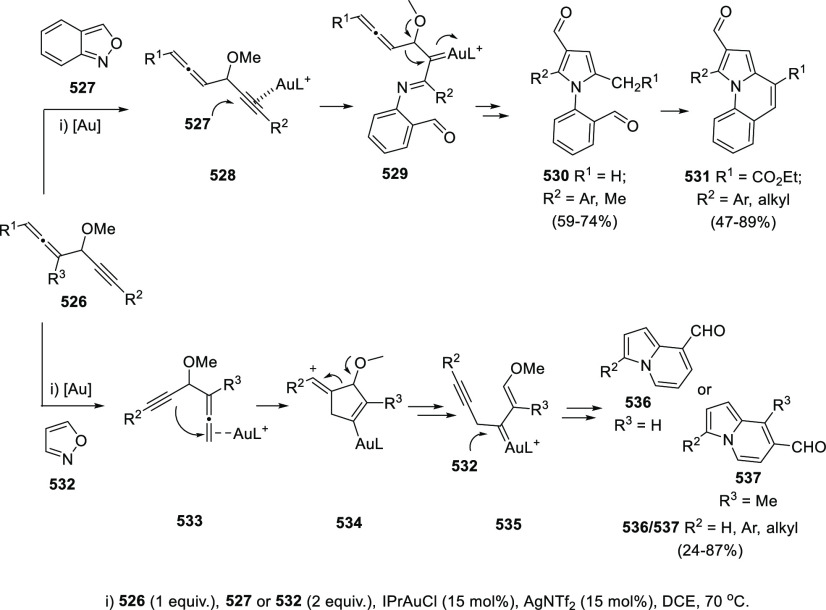
Reaction of Methoxyallenes with Anthraniland Isoxazole through Gold-Carbene Intermediates

Liu and co-workers have reported a methoxy-assisted allene migration to explain the synthesis of pyrroles **530** and pyrrolo[1,2-*a*]quinoline derivatives **531** from 4-methoxy-1,2-dienyl-5-ynes **526** with anthranil (**527**). Anthranil is proposed to attack the π-activated alkyne moiety in complex **528** (formed in the presence of gold salts), generating α-imino gold carbene intermediate **529**. 1,2-Allene migration and further gold-mediated aza-cyclization would furnish pyrroles **530**. When allene ester systems are employed, subsequent aldol reaction would explain evolution toward the polycyclic structures **531** (Scheme 88, top).^327^ On the other hand, reaction of 4-methoxy-1,2-dienyl-5-ynes **526** with isoxazole (**532**) as nucleophile provided the indolizine skeleton **536** and **537** under identical reaction conditions, unravelling a different mechanistic pathway. In this case, alkyne attack to the π-activated allene moiety in gold complex **533** would produce cyclic intermediate **534**, which afer methoxy-assisted ring-opening and isomerization could lead to vinyl gold carbene **535**. Nucleophilic attack of the isoxazole unit to the gold carbene carbon followed by a cascade azacyclization/rearrangement and aromatization would explain the observed indolizine compounds **536** and **537** (Scheme 88, bottom).^328^ Selenium-based π-acid-type catalysis has been used for the preparation of α,β-unsaturated α′-alkoxy ketones from alkoxy-allenes through alkoxy migration.^329^

### 3.4 Allenols in Chirality Transfer Processes

The development of new strategies to provide enantioenriched molecules constitutes one of the principal interests of the chemistry community. During the past decade, allenol-based reactions have also provided a notorious and increasing number of chirality transfer methodologies to get access to a wide family of enantioenriched structures. Chirality transfer from both central and axially chiral allenols has been reported. Also, it has been described the use of racemic allenols as precursors for the obtention of enantioenriched final compounds employing enzymatic catalysis, optically pure ligands in metal catalysis, and hybrid methodologies.

#### 3.4.1 Central-to-Central Chirality Transfer

**Scheme 89:**
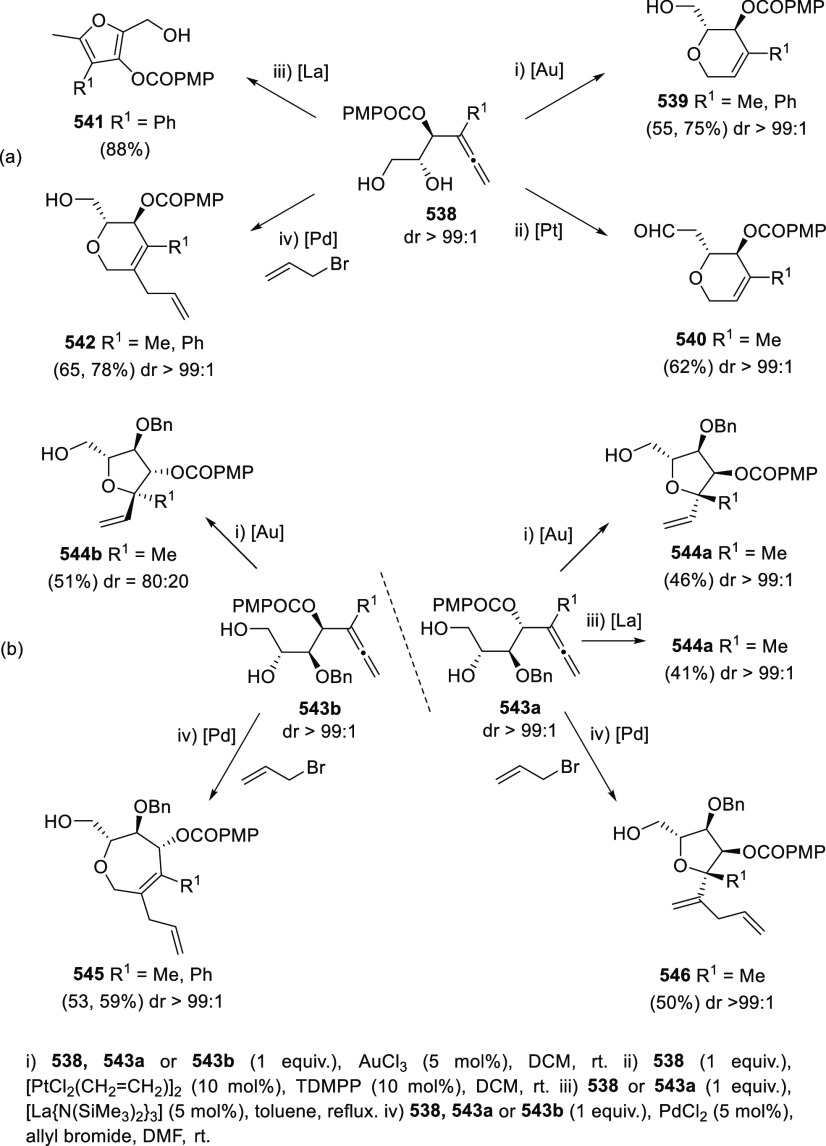
Synthesis of Enantiopure Tetrahydrofurans, Dihydropyrans, and Tetrahydrooxepines through Metal-Catalyzed Cyclization of Optically Pure β,γ- and γ,δ-Allendiols

The Alcaide and Almendros research group has described the synthesis of optically pure dihydropyran, tetrahydrofuran, and tetrahydrooxepine skeletons from enantiopure β,γ- and γ,δ-allendiols. Also, furan systems were synthesized. The methodology revealed an intriguing selectivity toward the oxycyclization reaction of secondary hydroxyls *versus* primary hydroxyls in both β,γ- and γ,δ-allendiols. The choice of the metal catalyst and the appropriate substituent was found to be crucial to achieve the optimal regioselectivity. Thus, β,γ-allendiols **538** reacted with gold(III) salts to produce the dihydropyran skeleton **539** through a *6-endo* cycloisomerization process involving the secondary hydroxyl group and the terminal allenic carbon (Scheme 89, reaction a, top right). Interestingly, allendiol **538** provided carbaldehyde **540** in the presence of platinum salts, from a similar *6-endo* cycloetherification reaction followed by a subsequent oxidation (Scheme 89, reaction a, bottom right). In contrast, furan **541** was obtained when β,γ-allendiol **538** was exposed to a lanthanide complex under catalytic conditions, through a *5-exo* cyclization toward the central allenic carbon (Scheme 89, reaction a, top left). Although palladium salts failed in promoting an effective cycloetherification to yield dihydropyrans **539** from allendiols **538**, the use of a Pd(II) catalysts and allyl bromide as coupling counterpart smoothly led to substituted dihydropyrans **542** (Scheme 89, reaction a, bottom left). This transformation revealed a selective *6-endo* cyclization/cross-coupling cascade reaction toward the terminal allenic carbon. On the other hand, benzyloxy homologous γ,δ-allendiols **543a** provided dihydrofurans **544a** under gold(III) catalytic conditions (Scheme 89, reaction b, top right). Noteworthy, diastereomer **543b** provided the corresponding 5-membered oxacycle **544b** through a related *5-exo-trig* cyclization, but exhibiting a decrease in the diastereoselectivity (Scheme 89, reaction b, top left). Although platinum catalysts did not show efficiency in the conversion of γ,δ-allendiols into oxacycle systems, lanthanide complexes provided dihydrofuran **544a** in similar yields as gold salts (Scheme 89, reaction b, right). More interestingly, palladium-catalyzed cross-coupling reaction conditions unravelled a dramatic change in the regiochemistry of the oxycyclization reaction depending on the absolute configuration of the starting material. Thus, γ,δ-allendiols **543b** provided the substituted tetrahydrooxepine **545** through a rare *7-endo-trig* cyclization, while diastereomer **543a** yielded tetrahydrofuran **545** through a *5-exo-trig* cyclization toward the inner allenic carbon (Scheme 89, reaction b, bottom).^330^

More recently, the same research group has reported the metal-based chemoselective aza- *versus* oxy-cyclization reaction of enantiopure α-amino-β-hydroxyallenes. Gold salts were reported to selectively promote the *5-endo* azacyclization toward the synthesis of optically pure 2,5-dihydro-1*H*-pyrroles, while the palladium cyclization/cross-coupling cascade strategy yielded 6-dihydro-2*H*-pyrans through a *6-endo* cycloetherification reaction.^331^ Also, optically pure β-lactam-tethered allenols were employed as chirality transfer reagents for the preparation of a wide variety of enantiopure polycyclic structures, such as morpholines, oxocines, dioxonines,^332^ and the already mentioned tetramic acids **510** and spirolactams **511** (see Scheme 86),^322^ through halogen or selenium-promoted reactions, respectively.

**Scheme 90:**
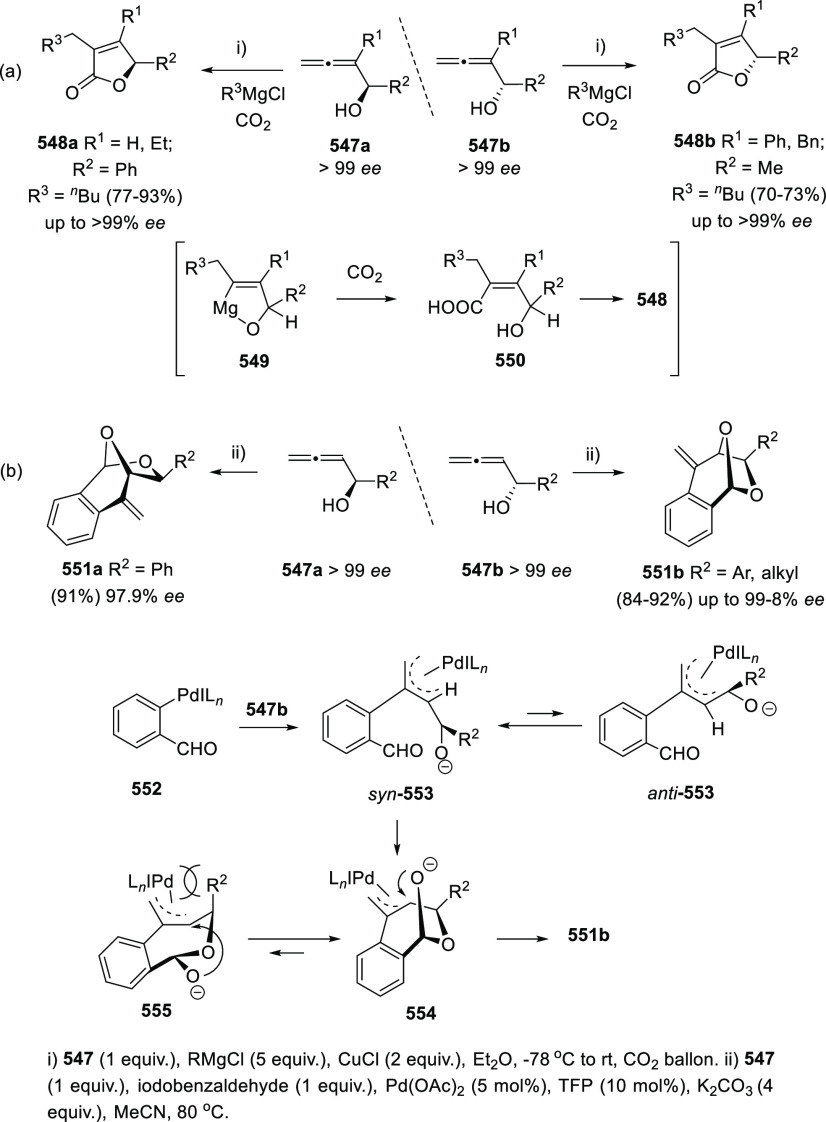
Metal-Catalyzed Cascade Oxycyclization from Enantiopure Allenols

Ma and collaborators have described the synthesis of enantiopure oxacycles from optically pure allenols presenting a tetrahedral chiral carbon through metal-catalyzed cascade processes involving oxycyclization steps. Highly substituted 2(5*H*)-furanones **548a**/**548b** were obtained by treatment of allenols **547a**/**547b** with Grignard reagents and CO_2_ atmosphere (Scheme 90, reaction a). The proposed reaction mechanism starts with the insertion of the organometallic reagent into the terminal allenic double bond, generating the cyclic intermediate **549** by Mg–O interaction. Reaction with CO_2_ would produce the corresponding γ-hydroxy-*Z*-alkenoic carboxylic acid **550**, which could undergo lactonization to yield the observed butenolides **548** without racemization.^333^

In a different approach, oxa-bridged benzocycloheptanes **551a**/**551b** were synthesized by reaction between optically pure allenols **547** and iodobenzaldehyde in the presence of palladium salts and a base (Scheme 90, reaction b). In this case, initial oxidative addition of Pd(0) species into the aryl halide would provide intermediate **552**. Then, carbopalladation of the corresponding allenol **547** (**547b** in Scheme 90, reaction b, bottom) would produce both π-allyl intermediates *anti*-**553** and *syn*-**553**, being the latter the most sterically favored. Intramolecular attack of the alkoxide to the carbaldehyde group in *syn*-**553** would lead to diastereomers **554** and **555**. The observed diasteroselectivity of the overall process could be explained by selective oxycyclization toward the allylic position in **554**, providing the less sterically hindered oxa-bridged benzocycloheptanes **551b** from allenes **547b**.^334^

**Scheme 91:**
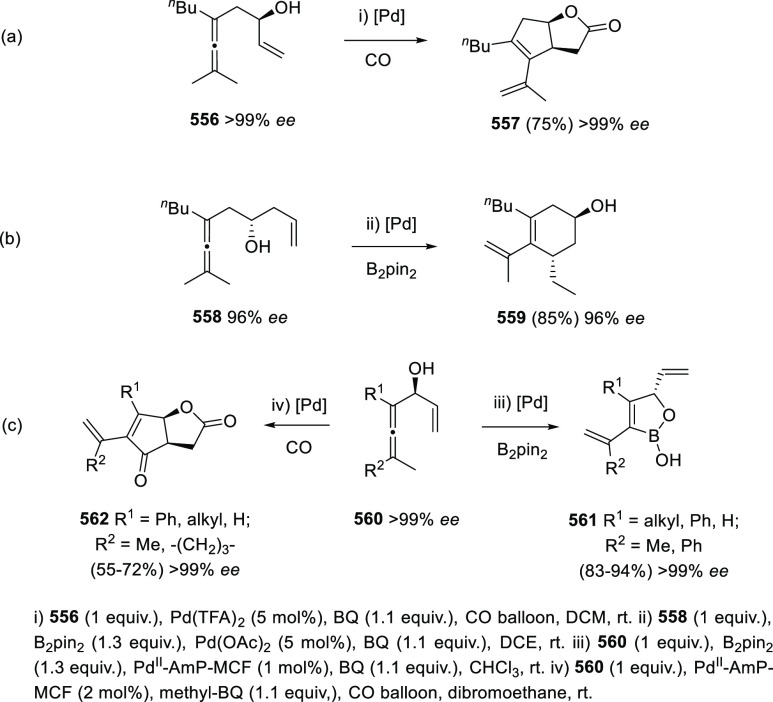
Enantioenriched Enallenols as Chirality Transfer Reagents in Palladium-Catalyzed Cyclization Reactions

Taking advantage of the versatile chemical behavior of the enallenol skeleton, Bäckvall research group has reported the preparation of a wide variety of molecules exhibiting high structural diversity and up to two sterogenic centers in optically pure form. Enantioenriched enallenol **556** reacted with Pd(TFA)_2_ under CO atmosphere to yield bicyclic lactone **557** with complete enantioretention (Scheme 91, reaction a).^335^ The reaction mechanism to explain this transformation would include a palladium-mediated allene-alkene carbocyclization reaction, followed by a sequential carbonylation/lactonization step, resembling previous palladium-lactonization of enallenols (see Scheme 69, section 3.2). Homologous enallenol **558** provided cyclohexanol skeleton **559** in good yield in the presence of B_2_pin_2_, exhibiting also full enantioretention (Scheme 91, reaction b). Homogeneous Pd(OAc)_2_ catalysis was employed to achieve this transformation, which should imply a related allene-alkene carbocyclization followed by ligand exchange with the borane reagent and reductive elimination.^336^ Noteworthy, coordination of the hydroxyl unit with the metal ion at the first stages of the reaction mechanism is proposed to be crucial to explain formation of compounds **557** and **559**.

In contrast, enallenols **560** were transformed into oxaboroles **561** by reaction with B_2_pin_2_ under heterogeneous palladium catalysis (Scheme 91, reaction c, right). Enantioenriched allenols **560** were prepared through kinetic resolution of the racemic mixture using *Candida Antarctica* Lipase B, showing >99% *ee*. Interestingly, despite of the palladium ability to induce racemization in allylic alcohols, no loss of optical purity was detected.^210^ The same catalytic system provided γ-lactones **562** in optically pure manner from reaction of allenols **560** under CO atmosphere. Moderate to good yields were achieved with full retention of the enantiopurity in final compounds **562** (Scheme 91, reaction c, left).^337^ Amino-supported heterogeneous palladium has also been recently used by Bäckvall et al. in a domino reaction from related enallenols with alkynes. The chelating activity of the hydroxyl group is responsible of the observed diastereoselectivity.^338^

More particular methodologies for the synthesis of stereodefined oxacycles have been recently reported. Anderson et al. have described the reaction of enantiopure cyclic alkynyl carbonates with palladium catalysts to yield alkynyl tetrahydrofuran systems through *in situ* generated allenol-palladium intermediates.^339^ Guinchard and collaborators have employed gold-catalyzed cyclizations of tetrahydro β-carboline structures decorated with enallenol motifs for the synthesis of optically pure decahydrofuro[2,3-*f*]indolo-[2,3-*a*]quinolizines. Gold(I) salts have been reported to exhibit optimal reaction conversions providing full enantioretention and good control on the enantioselectivity of the newly formed sterogenic centers.^340^

**Scheme 92:**
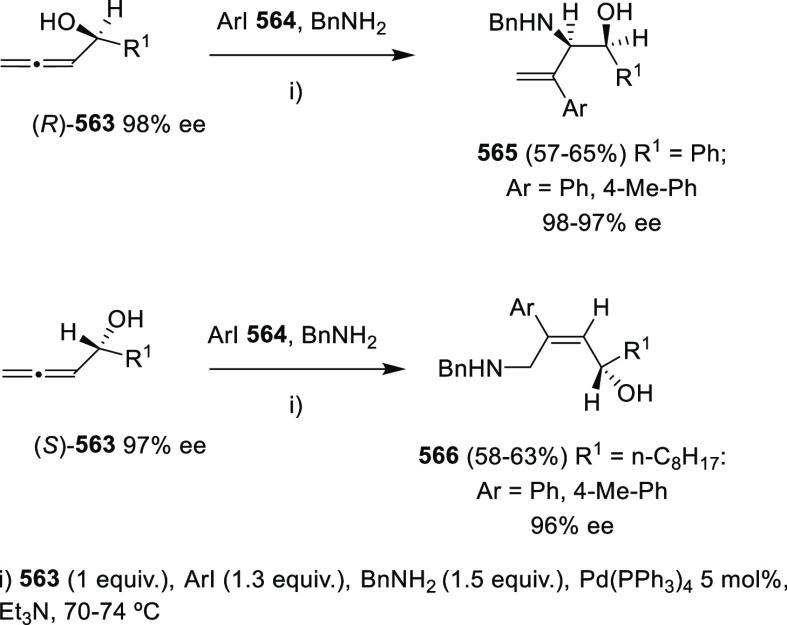
Asymmetric Multicomponent Reaction of Enantioenriched Allenols, Aryl Iodides, and Amines under Palladium Catalysis

Opposite to the most commonly reported cyclization reactions, intermolecular processes involving enantioenriched allenols in asymmetric synthesis are scarce. Taking advantage of the chelating effect of the hydroxyl group, as explored in section 3.3, palladium-catalyzed multicomponent reactions of different allenols **563**, aryl iodides **564** and benzylamine proceeded with no loss of enantiopurity, even in the absence of enantiopure phosphine ligands. Interestingly, a change on the allenol substitution provided a dramatic change on the regioselectivity. Thus, phenyl-substituted allenols yielded homoalylic alcohols **565**, while alkyl-substituted substrates provided allylic alcohols **566**, through the attack of the amine to the terminal allenic carbon (Scheme 92).^341^

#### 3.4.2 Axial-to-Central Chirality Transfer

**Scheme 93:**
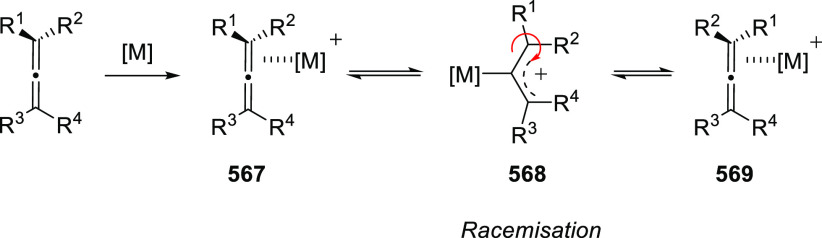
Racemization of Axially Chiral Allenes by Metal-Coordination

Opposite to central-to-central chirality transfer methodologies from enantioenriched allenols, reports on axial-to-central chirality transfer using axially chiral enantioenriched allenols are rare. One of the principal challenges to circumvent is the ease of racemization of the allene moiety under metal catalyzed conditions. Normally, metal activation of the allene unit starts with π-coordination of one of the double allenic bonds with the metal ion in a η^2^ complex **567** (Scheme 93). When such intermediates are in equilibrium with the corresponding π-allyl cations **568**, free bond rotation falls into loss of the optical purity of the allene moiety. Avoiding the above-mentioned equilibrium by stabilizing the η^2^ complex constitutes one of the most recurrent strategies to achieve axial chirality transfer reactions in allenes. The choice of the metal catalyst and the appropriate substituents on the allene core are the principal tools to achieve a successful chirality transfer transformation.

**Scheme 94:**
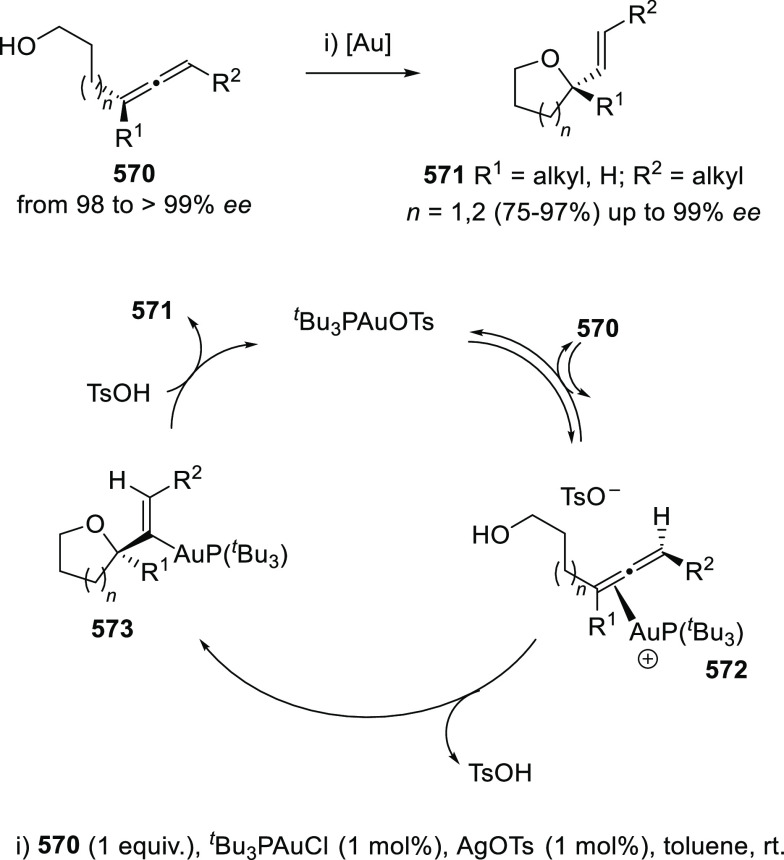
Chirality Transfer in Gold-Catalyzed Cycloisomerization Reactions of γ- and δ-Allenols

In the context of allenol-based reactions, Lalic and co-workers have reported the first synthesis of teytrahydrofuran and tetrahydropyran systems bearing a tetrasubstituted stereogenic center. Substituted β- and γ-allenols **570** were submitted to gold catalysis providing the expected oxycyclization products **571** with good to excellent yields and a chirality transfer of up to 99% (Scheme 94). Gold(I)-based salts bearing a tosylate group together with electron-rich sterically hindered phosphine ligands were found to provide the best results, also promoting complete *E*-selectivity in the double bond formation in compounds **571**. A plausible reaction mechanism would start from coordination of the metal species to the proximal double bond in intermediate **572**, followed by carbometalation to furnish vinyl gold intermediate **573** without the occurrence of allene racemization. Opposite to Widenhoefer’s statement about digold alkenyl intermediates as catalyst resting states,^300^ monogold(I) intermediate **573** is herein presented as the most likely resting state due to the weakly electrophilicity of the gold complex, and the highly coordinating character of the counterion.^342^

**Scheme 95:**
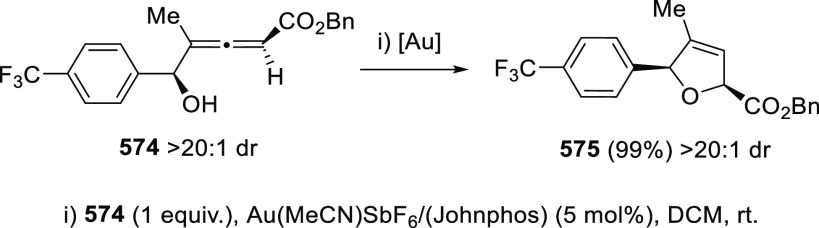
Synthesis of Optically Active Dihydrofurans through Gold-Catalyzed Oxycyclization of Enantioenriched Allenols

Similarly, Yin’s research group has reported the synthesis of substituted dihydrofurans **575** showing up to 20:1 dr through a gold-catalyzed oxycyclization process. Allenols **574**, exhibiting both axial and central chirality, have been prepared according to the previously mentioned asymmetric Cu-catalyzed alkynylogous aldol reaction (Scheme 22, reaction a), and successfully converted into cyclic structures **575**, current motifs in anti-Alzheimer and Down’s Syndrome drugs (Scheme 95).^145^

**Scheme 96:**
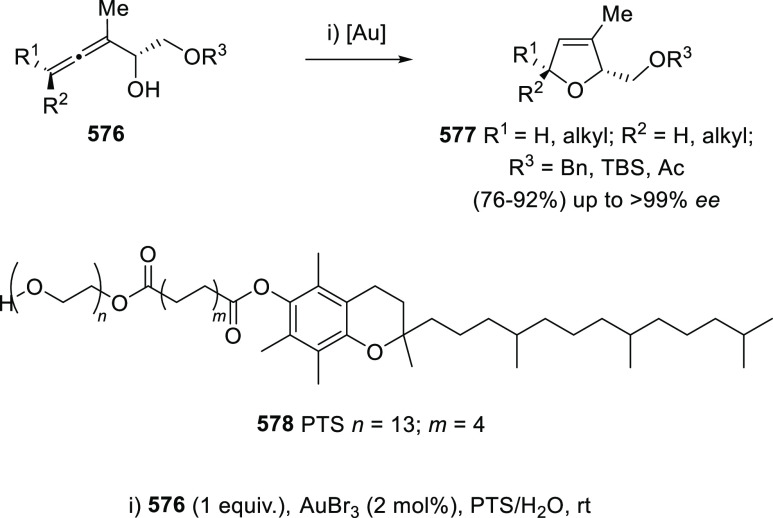
Chirality Transfer in Micellar-Supported Gold-Catalyzed Oxycyclization Reactions of Allenols

A different approach based on gold catalysis has been reported by Krause and Lipshutz et al., following their interest in discovering new micellar systems to provide greener methodologies, high catalyst efficiency and recyclability.^281^ It has been reported that micellar catalysis in aqueous media was also effective for the synthesis of enantioenriched structures from α-allenols, employing poly(oxyethyl)-α-tocopheryl sebacate (PTS, **578**) as amphiphile and AuBr_3_ as metal source. Dihydrofuran structures **577** bearing two stereogenic centers were achieved, exhibiting good to excellent yields and complete chirality transfer (Scheme 96).^343^

**Scheme 97:**
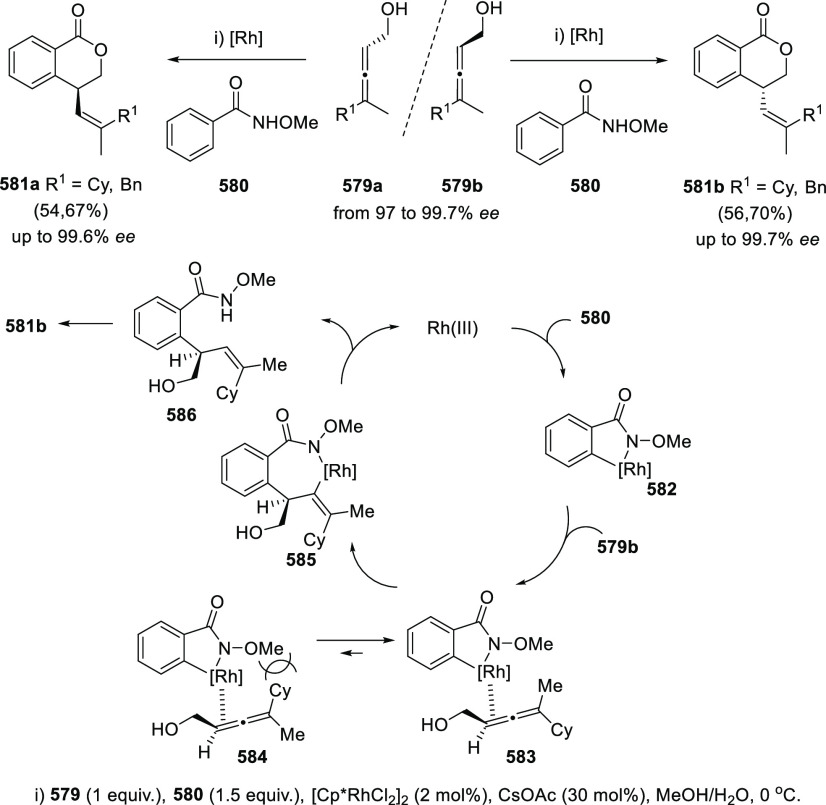
Chirality Transfer on Rhodium-Mediated C–H Insertion/Lactonization Reaction of *N*-Methoxybenzamide with Allenols

Ma and collaborators have synthesized optically pure lactones **581a** and **581b** from *N*-methoxybenzamide **580** and enantioenriched substituted allenols **579a** and **579b** under rhodium catalysis conditions (Scheme 97). Axial chirality in starting allenols **579** was fully transferred into lactones **581** exhibiting a stereogenic center with moderate yields. The mechanistic proposal would start with rhodation of the *N*-methoxybenzamide unit **580** to yield cyclic intermediate **582** (Scheme 97, bottom). Coordination of the metal ion to the less substituted allenic bond would then lead to complex **583**, in equilibrium with the less favored intermediate complex **584**. Carbometalation of the allenic double bond from the less sterically hindered adduct **583** could explain the *E*-selectivity observed, furnishing vinyl rhodium compound **585**, which may suffer protonolysis to give **586**. Eventual lactonization would explain the experimentally observed cyclic structures **581**.^344^

**Scheme 98:**
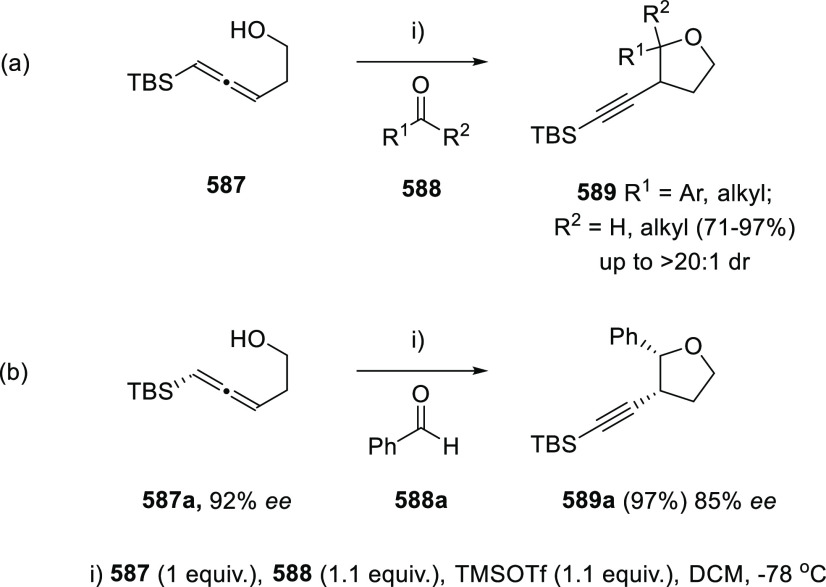
Tetrahydrofuran Synthesis through Prins-type Cyclization of β-Allenols

Metal-free strategies regarding axial-to-central chirality transfer are scarce. Zhang and Bao and collaborators have recently reported the NIS-promoted allenol oxycyclization to yield dihydropyran systems showing central chirality.^345^ Sakaguchi and Ohfune and co-workers have described the enantiomeric version of the Prins-type reaction of allenols with carbonyls. β-Allenols **587** bearing a terminal silyl group were selected as chirality transfer agents, reacting through an uncommon *5-endo-trig* cyclization in the presence of TMSOTf as Lewis acid catalyst and both aldehydes and ketones as reaction counterparts. Silylalkynyl-decorated tetrahydrofurans **589** were obtained as single diastereomers, exhibiting two stereogenic centers (Scheme 98, reaction a). To test the axial-to-central chirality transfer efficiency, enantioenriched allenol **587a** was synthesized with a 92% *ee*. Reaction of **587a** with benzaldehyde (**588a**) and TMSOTf as acid catalyst provided the expected oxycyclization product **589a** showing an enantiomeric excess of 78%. Increasing the Lewis acid load up to 1.1 equiv allowed an 85% *ee* in final tetrahydrofuran **589a**, showing solely slight racemization during the reaction (Scheme 98, reaction b).^346^

#### 3.4.3 Metal-Based Catalysts as Chirality Transfer Agents

**Scheme 99:**
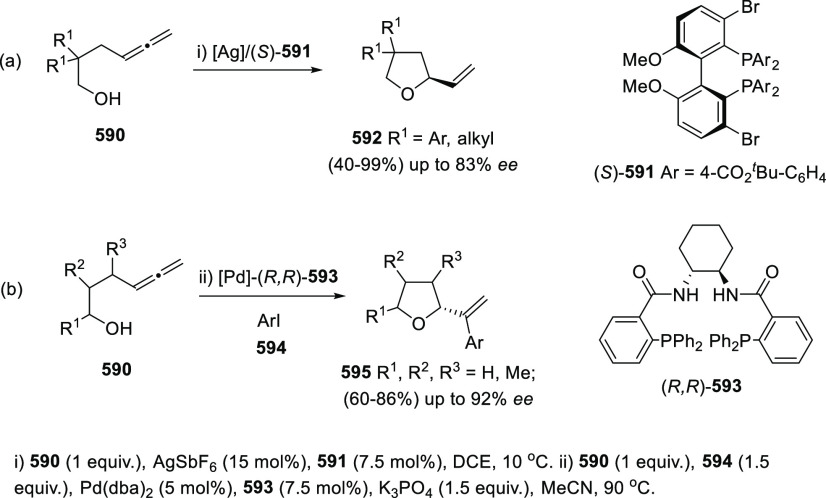
Synthesis of Enantioenriched Vinyl Tetrahydrofuran Systems through Metal-Catalyzed/Phosphine Ligand-Mediated Oxycyclization

Metal catalysis offers a wide variety of alternatives to achieve an efficient chirality transfer with associated formation of enantioenriched molecules. The most traditional and extended strategy is based in the joined use of the metal along with a chiral phosphine ligand which induces enantioselectivity during the reaction. Michelet and Scalone and collaborators have synthesized an unprecedented bromine-decorated diphosphine ligand (*S*)-**591** showing chiral atropisomerism. The catalytic system Ag/**591** was applied for the synthesis of vinyl tetrahydrofurans **592** through a *5-exo-trig* cyclization of γ-allenols **590**. Yields were moderate to excellent, and enantiomeric ratios up to 91.5:8.5 were stated (Scheme 99, reaction a).^347^ Ma’s research group has devised an enantioselective palladium-catalyzed cross-coupling reaction of γ-allenols **590** with aryl iodides **594** yielding styryl tetrahydrofuran systems **577** through a similar *5-exo-trig* cyclization. Optically pure diphophine (*R,R*)-**591** was employed, finding enantioinversion in vinyl-substituted tetrahydrofuran structures **595** with respect to systems **592**. Good yields and up to 92% *ee* were stated (Scheme 99, reaction b).^348^

**Scheme 100:**
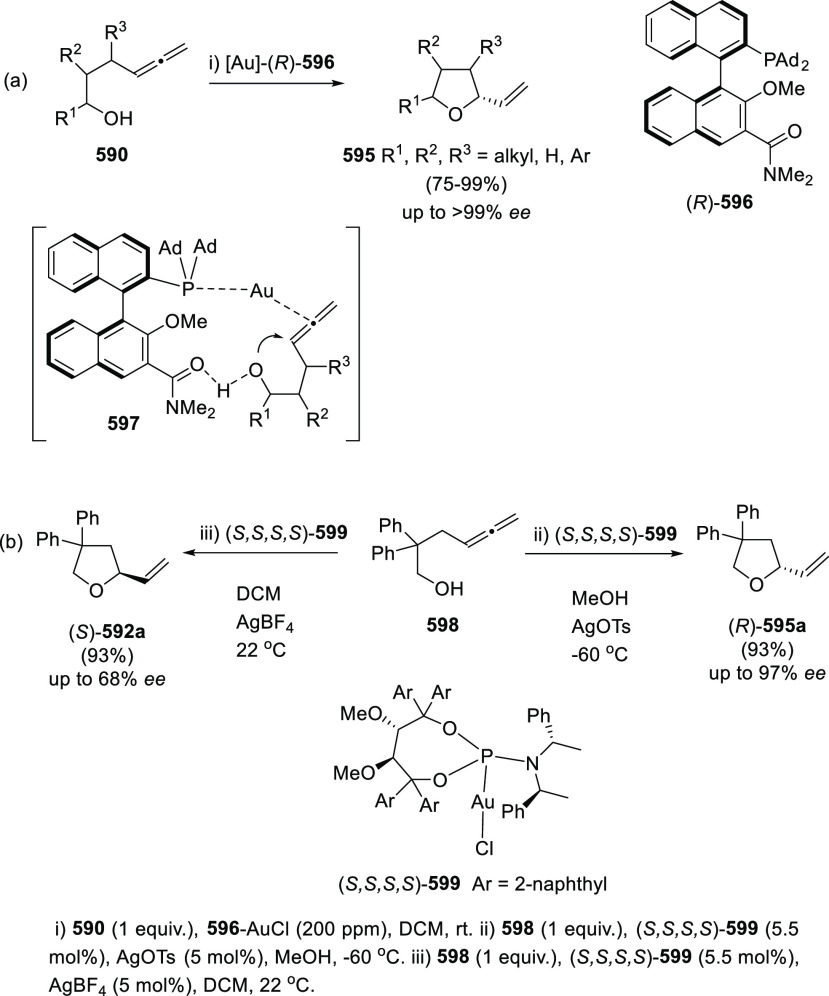
Synthesis of Enantiopure Vinyl Tetrahydrofurans through Chirality Transfer from Gold-Based Catalytic Systems

Zhang’s research group has envisioned an accelerative gold catalysis by ligand–metal coordination to γ-allenols **590** providing vinyl tetrahydrofuran skeletons **595** (Scheme 100, reaction a). Opposite to classic enantiopure ligand approaches, where chiral induction is frequently based in sterically hindered ligands which normally show a decelerating effect in the reaction rate, Zhang’s ligand (*R*)-**596** bearing an amide remote group exhibited an 88-fold rate increase compared with unsubstituted bisphenyl- or bisnaphthyl phosphines. Simoultaneous metal-allene and carbonyl-hydroxyl coordination in intermediate **597** explains the enhancement of the catalytic activity, yielding tetrahydrofurans **595** with good to excellent yields and enantioselectivities up to >99% *ee*. Also, catalyst loadings as low as 200 ppm were allowed.^349^ A similar strategy was applied to the synthesis of enantioenriched dihydrofuran structures from *in situ* generated α-allenol systems.^350^ The 5*-exo-trig* oxycyclization of γ-allenol **598** has also been in the focus of an intriguing case of enantioinversion in final vinyl tetrahydrofuran molecules (*S*)-**592a** and (*R*)-**592a** (Scheme 98, reaction b). Fürstner et al. have found that even under the same optically pure gold catalyst **599**, formed from AuCl and phosphoramidite (*S*,*S*,*S*,*S*)-**599**, enantioselection could be achieved by changing the solvent, temperature, and counterion. Moreover, a synergistic effect between the three parameters could be perform, achiving enantiomeric excesses from 97% *ee* in (*R*)-**595a** (Scheme 100, reaction b, right) to 68% *ee* in (*S*)-**592a** (Scheme 100, reaction b, left). Experimental and computational studies revealed that a change in the rate determining step lays on the base of the change in the sterochemical outcome. Protic solvents and coordinating counterions supported an additive-assisted reaction mechanism favoring the (*R*)-**595a** isomer. Also, temperature has a dramatic entropic effect, promoting the additive-assisted mechanism on cryogenic conditions providing (*R*)-**579a** isomer as major compound.^351,352^

**Scheme 101:**
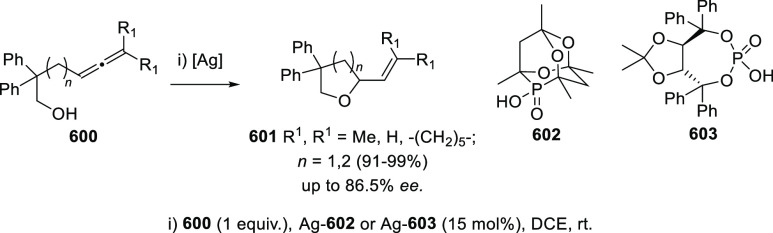
Silver-Catalyzed Enantioselective Oxycyclization of Allenols

Related enantiopure oxaphosphorous ligands **602** and **603** have been used in the context on an unprecedented enantioselective silver-catalyzed oxycyclization of allenols. Reaction of Ag_2_CO_3_ salts with the corresponding phosphoric acid yielded catalyst complexes Ag-**602** and Ag-**603**. Treatment of different allenols **600** with the preprepared catalytic species allowed the synthesis of vinyl tetrahydrofuran and tetrahydropyran compounds **601** (Scheme 101). Also, furanones were obtained starting from the corresponding allenic carboxilyc acids. DFT calculations on this transformation pointed to ionic interactions between ligand and substrate as the major forces contributing to the enantioselectivity of the process.^353^

**Scheme 102:**
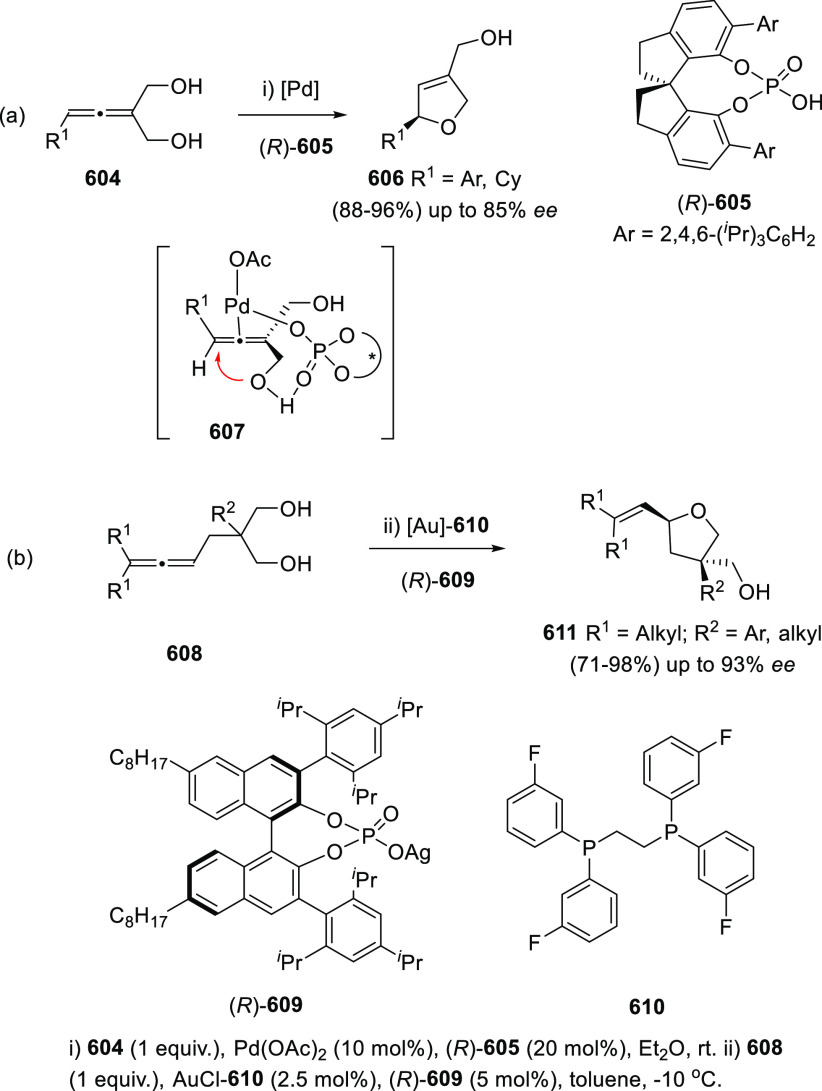
Metal-Catalyzed Desymmetrization of Allendiols

Chemical desymmetrization of prochiral allendiols has been accomplished using palladium catalysts and enantiopure phosphoric acids as ligands. α,α′-Allendiols **604** were submitted to Pd(OAc)_2_ catalysis in the presence of catalytic amounts of chiral ligand (*R*)-**605**, generating dihydrofurans **606** in good to excellent yields, and practical enantiomeric excesses up to 85% *ee* (Scheme 102, reaction a). Adduct **607** was proposed as the stereodetermining intermediate, showing a dual interaction of the catalytic system and the allenol unit through both metal-coordination to the central allenic carbon and hydrogen bonding with the hydroxyl group.^354^ In a similar but conceptually different approach, γ,γ′-allendiols **609** were converted into enantioenriched tetrahydrofuran derivatives **611** through a gold-catalyzed oxycyclization (Scheme 102, reaction b). In this case, enantiopure phosphoric silver salts (*R*)-**609** were introduced in the reaction system as chiral counterions, while achiral phosphine ligands such as **610** are linked to the reactive gold ion and are used as enantioselectivity modulators. This methodology allowed the generation of two stereogenic centers in one single operational step, furnishing oxacycles **611** with good to excellent yields and up to 93% *ee*.^355^

**Scheme 103:**
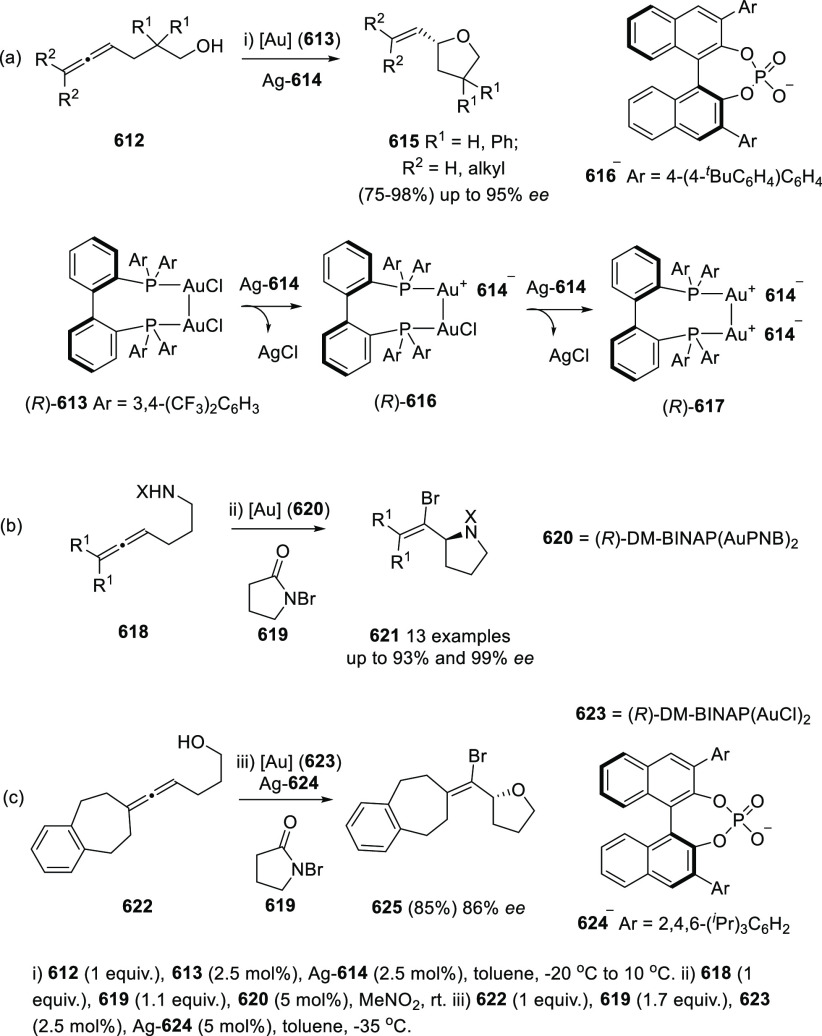
Chirality Transfer from Enantiopure Chiral Ligands and Chiral Counterions on Gold-Catalyzed Oxycyclization of γ-Allenols

Different authors have investigated the synergistic effect of both enantiopure chiral ligands and enantiopure counterions to achieve higher enantioselectivities without loss of catalytic activity. Regarding allenol oxycyclizations, Mikami et al. have described the use of neutral dinuclear gold complexes **613** as catalytic species with improved activity for the *5-exo-trig* cyclization of substituted γ-allenols (Scheme 103, reaction a). The optimal reaction conditions were found when same catalytic amount of **613** and the silver salt Ag-**614** bearing an enantiopure chiral phosphoric acid counterion were added. Vinyl tetrahydrofurans **615** were obtained with good to excellent yields and up to 95% *ee*. According to experimental studies, it has been proposed species (*R*)-**616** from ligand exchange with one equivalent of silver complex Ag-**614** as the catalytically active system. Complex (*R*)-**613** in the absence of silver salts was ineffective for the indicated transformation, while complex (*R*)-**617** from the addition of two equivalents of Ag-**614** showed lower yields and enantioselectivity (Scheme 103, reaction a).^356^ Toste and co-workers described the enantioselective aza-cyclization/halogenation tandem reaction of allenamides **618** with brominating reagent **619** to yield bromovivnyl pyrrolidine structures **621** under gold catalysis in the presence of BINAP-type ligands (Scheme 103, reaction b). The methodology also included one example of *5-exo-trig* oxycyclization of γ-allenol **622** to produce bromovinyl tetrahydrofuran **625** (Scheme 103, reaction c). This transformation promoted by gold catalyst **623** provided a poor enantioselectivity (25% *ee*) of the indicated structure **625**. Interestingly, the combined use of **623** and silver salt Ag-**624** bearing a chiral counterion provoked a remarkable impact, both increasing the selectivity up to 86% *ee* and reverting the enantioselectivity compared to adducts **621**.^357^

**Scheme 104:**
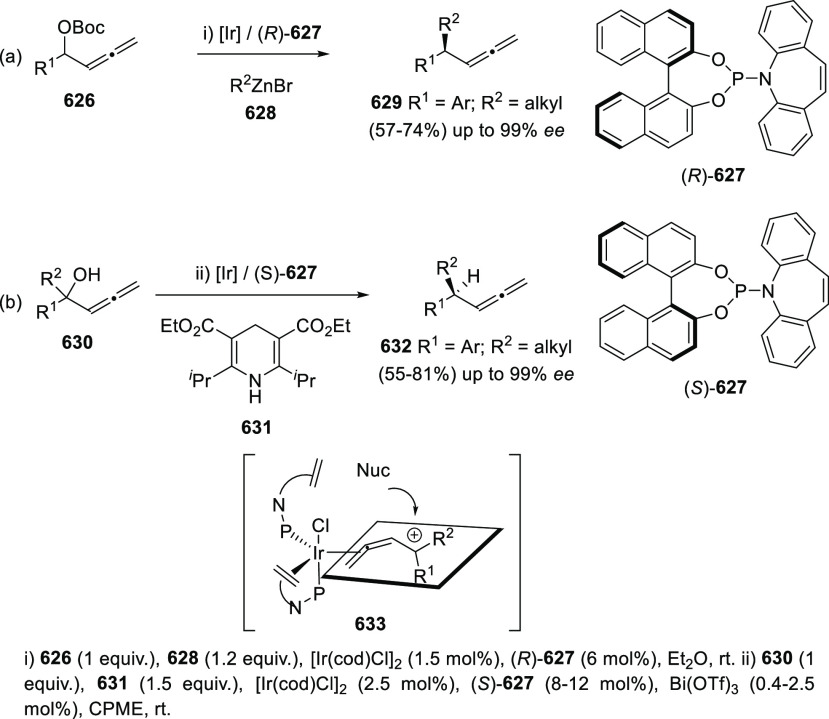
Enantioselective S_N_1-type Reactions Involving Iridium-Based Allenic Carbocations

Despite the widespread presence of carbocations in organic synthesis, its utilization as reaction intermediates in asymmetric synthesis is still scarcely described. The challenging facial discrimination in planar carbocation species has been mainly limited to diastereoselective-substrate control, or to the addition of an enantiopure chiral anion forming ion pairs with the carbocationic molecule. Carreira research group has envisioned an alternative approach for developing asymmetric S_N_1-type reactions in α-allenols and related systems, based in η^2^ coordination complex **633** (Scheme 104, bottom). Species **633** formed from interaction of iridium salts bearing an enantiopure chiral phosphine and the distal double bond of the allene moiety can be considered as mimics of diastereoselective-control substrates and constitutes one rare example of enantioselectivity in allenol transformations apart from oxycyclization processes. Thus, racemic Boc-protected α-allenols **626** reacted with organozinc nucleophiles **628** in the presence of iridium catalysts Ir-(*R*)-**627** to provide compounds **629** in practical yields and excellent enantioselectivities, through an asymmetric allene-transfer reaction (Scheme 104, reaction a).^358^ In addition, related methodology has been applied to describe the first example of an enantioselective reductive deoxygenation of tertiary alcohols. Thus, racemic α-allenols **630** were submitted to catalytic system Ir-(*S*)-**627** in the presence of Hantzsch ester analogues **631** as hydride source, providing enantioenriched allenes **632**. Kinetic and computational insights revealed the presence of η^2^ carbocationic complexes as the most plausible reaction intermediates (Scheme 104, reaction b).^359^

Different approaches to achieve asymmetric synthesis from racemic allenols and enantiopure catalysts also includes denitrogenative annulation of 1,2,3-benzotriazin-4(3*H*)-ones with allenes under nickel catalysis,^360^ synthesis of 1*H*-isochromene structures through copper catalyzed oxycupration/allylation reaction using optically pure phosphine ligands,^361^ the enantioselective synthesis of spiropentanes from hydroxymethylallenes catalyzed by Zn,^362^ the asymmetric palladium-catalyzed homoallenilation of amines,^363^ or the synthesis of cyclodextrin-tethered gold(I) carbene complexes as water-soluble and recyclable catalysts for several transformations including oxycyclizations of α- and γ-allenols.^364^

**Scheme 105:**
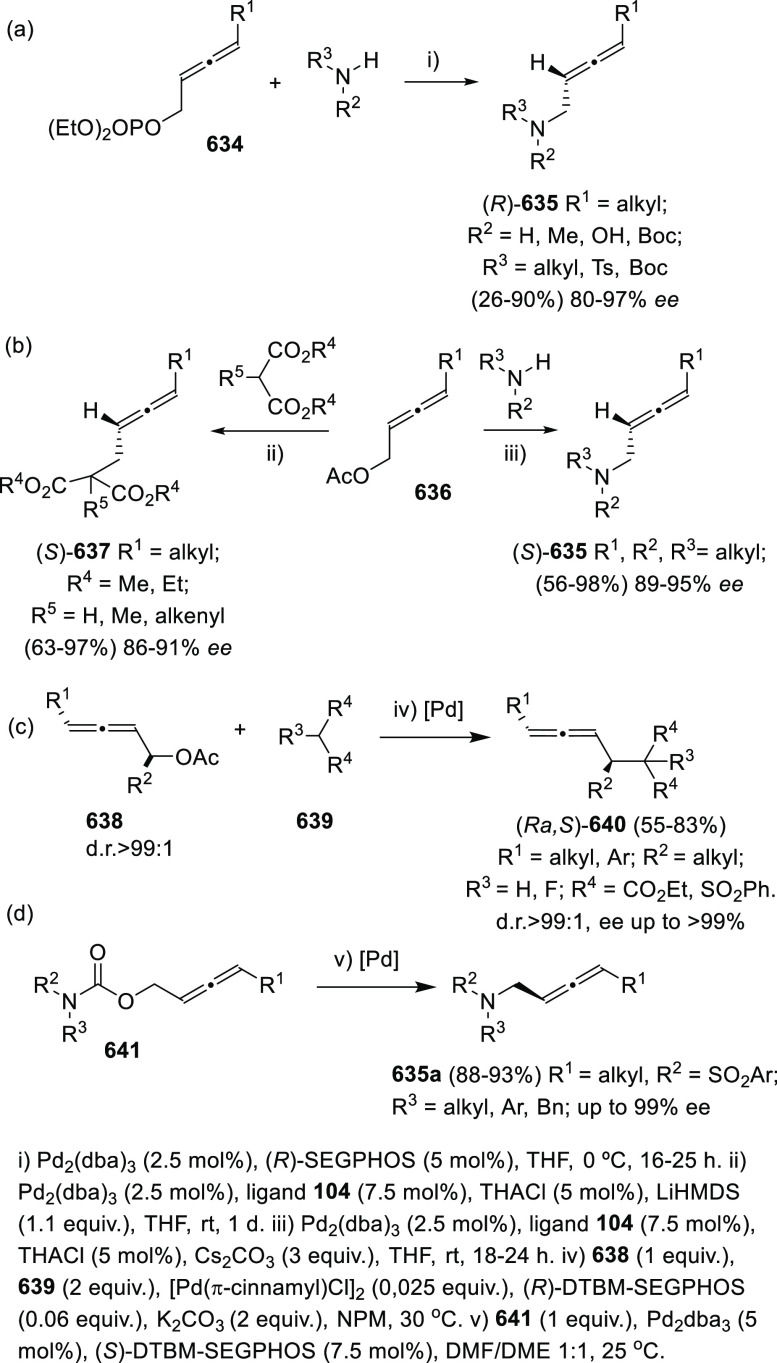
Palladium-Catalyzed Enantioselective Synthesis of Functionalized Allenes from Allenyl Esters

Racemic 2-(2′,3′-alkadienyl)malonates were obtained through the Pd(PPh_3_)_4_-catalyzed alkylation reaction of allenyl acetates with malonates^365^ while racemic allenes bearing a quaternary carbon center α to the cumullene were prepared by [Ir(cod)Cl]_2_/dppe-catalyzed allylic alkylation of 1,1-disubstituted-2,3-butadienyl acetates with malonates.^366^ A smart approach for obtaining optically active allenes is the direct preparation from achiral allenyl acetates, allenyl phosphonates, and allenyl carbamates via π-allylmetal intermediates, taking advantage of the great leaving aptitude of the acetate, phosphonate, and carbamate moieties. Imada, Murahashi, and Naota achieved the metal-catalyzed synthesis of enantioneriched α-allenamines **635** by asymmetric amination of allenyl phosphonates **634** using Pd2(dba)3CHCl3 as the palladium source and (*R*)-SEGPHOS as the ligand (Scheme 105, reaction a).^367^ Starting from precursors 1, the same research group did also reported the asymmetric alkylation with 2-acetamidomalonate.^368^ Trost and co-workers accomplished the asymmetric synthesis of allenes (*S*)-**635** and (*S*)-**637** from allenyl acetates **636** by palladium-catalyzed dynamic kinetic reactions with both malonates and amines involving α-methylidene π-allylpalladium species (Scheme 105, reaction b).^369^ The optimized reaction conditions require the use of Pd2dba3 (2.5 mol %), phosphine **104** (7.5 mol %), THACl (tetrahexylammonium chloride) (5 mol %), and a base in THF. Hamada performed the same palladium-catalyzed reaction between allenyl acetates **636** and malonates but replacing Trost ligand with (*S*,*R*_P_)-DIAPHOXs, a chiral nonracemic diaminophosphine oxide, which results in the formation of axially chiral allenes (*R*)-**637** in good yields with up to 99% *ee*.^370^ The above-mentioned palladium-catalyzed amination of allenyl phosphonates,^371^ and the addition of malonates to allenyl acetates,^372,373^ have also been explored by Ma and collaborators in the context of achieving central as much as axial chirality from racemic allenes. The same research group has deeply investigated different allenyl esters in the presence of various nucleophiles yielding substituted allenes exhibiting both types of chirality. Thus, racemic allenyl acetates **638** have been reported to undergo a S_N_2′-type oxidative addition in the presence of palladium complexes and enantiopure (*R*)-DTBM-SEGPHOS as ligand. Echoing effect between the central and axial chirality is stated, providing enantioenriched allenes **640** showing both axial and central chirality (Scheme 105, recation c).^374^ Asymmetric allenylation of malonates have also been achieved using racemic allenyl carbonates. Selectivity between mono- and bis-allenylation is reported using Pd2(dba)3/(*R*)-DTBM-SEGPHOS as catalytic pair.^375^ In a related work, a smart approach to allenylamines exhibiting axial chirality has been accomplished through the palladium-catalyzed decarboxylative amination of allenyl carbamates **641**. Pd2(dba)3/(*S*)-DTBM-SEGPHOS catalytic pair promotes the loss of CO2 providing the corresponding π-allylpalladium intermediates. Further nucleophilic attack of the *in situ* generated amide ion yields the observed allenylamines **635a** exhibiting up to 99% ee and good to excellent yields (Scheme 105, reaction d).^376^

#### 3.4.4 Chirality Transfer in Enzymatic Catalysis

**Scheme 106:**
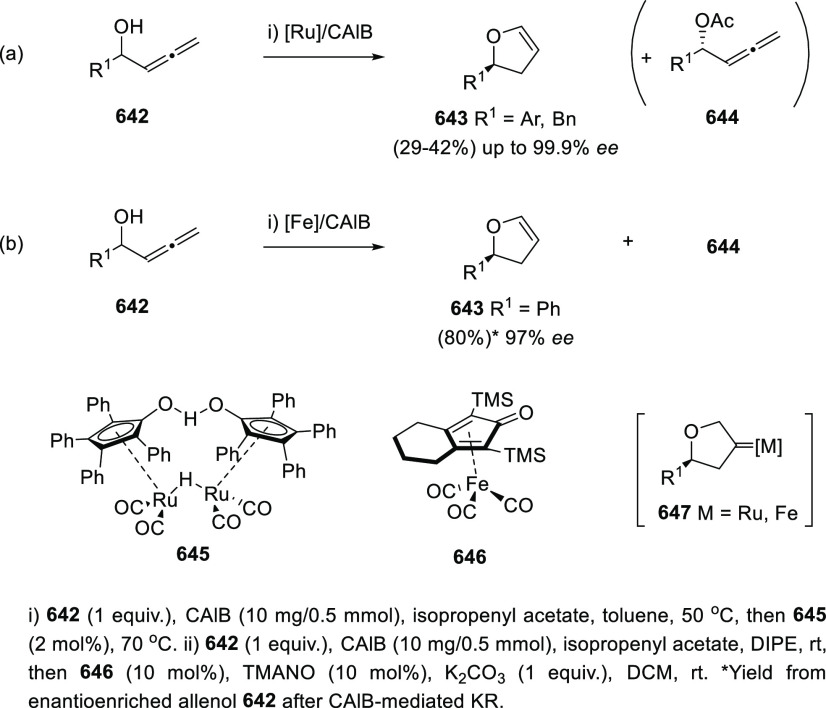
Hybrid Enzymatic/Transition Metal Catalyzed Synthesis of Dihydrofuran Compounds

Although enzymatic systems have been largely employed as biocatalysts for KR and DKR in allenol synthesis, strategies for the preparation of enantiopure compounds from racemic allenols including enzymatic resolution are almost unexplored. Bäckvall research group has contributed to develop this methodology with the synthesis of dihydrofuran and cyclobutanol skeletons with excellent enantiomeric excesses. During attempts to achieve an efficient DKR of allenols, Shvo catalyst (**645**) was used in combination with Candida Antarctica lipase B (CAlB) to promote the expected consecutive recemization and selective acetylation of α-allenols **642**. Despite the well-known activity of ruthenium catalyst **645** for the racemization of secondary alcohols, oxycyclization products **643** were found instead, along with the corresponding acetylated allenols **644** (Scheme 106, reaction a). Noteworthy, enantioselectivities in dihydrofuran skeletons **643** were remarkably higher in comparaison with any other standard oxycyclization procedure. In addition, mechanistic insights pointed to ruthenium carbene species **647** as reaction intermediates, explaining the eventual double bond isomerization found in final adducts **643** through a 1,2-H shift.^377^ Alternatively, a cheaper and less toxic approach based on iron catalyst **646** and same enzymatic system allowed the preparation of dydrofurans **643** through milder reaction conditions (Scheme 106, reaction b).^378^

**Scheme 107:**
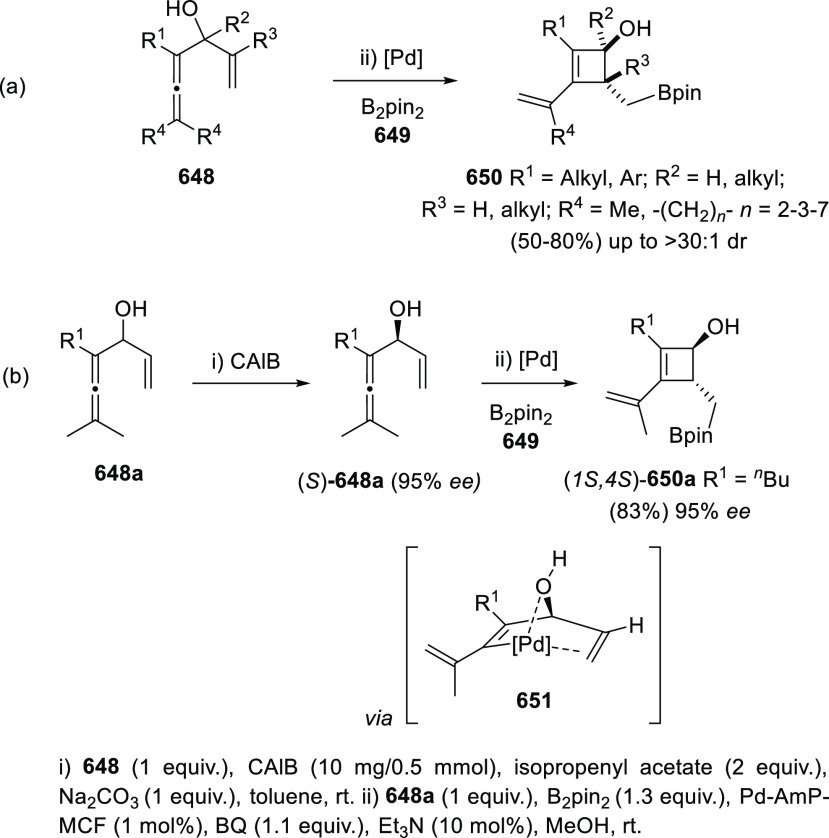
Hybrid Enzymatic/Transition Metal Catalyzed Synthesis of Enantioenriched Cyclobutenols

The same research group has also taken advantage of the fruitful reactivity of enallenol skeletons under palladium catalysis, previously mentioned in section 3.2, to perform the synthesis of cyclobutenol structures. Reaction of racemic enalenols **648** and boronic esters **649** in the presence of palladium nanoparticles yielded the four-membered ring systems **650** trough a tandem carbocyclization/borylation reaction. Moderate to good yields and high diastereoselectivities were observed. In addition, palladium nanoparticles were suspended on amino-decorated mesocellular foam (Pd-Amp-MCF), providing high recyclability and efficiency (Scheme 101, reaction a). In combination with biocatalyst CAlB, cyclobutenol structure (*1S*,*4S*)-**650a** was obtained with a good 83% yield from enantioenriched enallenol (*S*)-**648a**, and a high 95% *ee* (Scheme 107, reaction b). From the mechanistic point of view, hydroxyl group is proposed to perform a multiple role, promoting the carbocyclization process by coordination with the metal unit, and directing the stereoselectivity on final adducts **630** through intermediates **651**.^379^

**Scheme 108:**
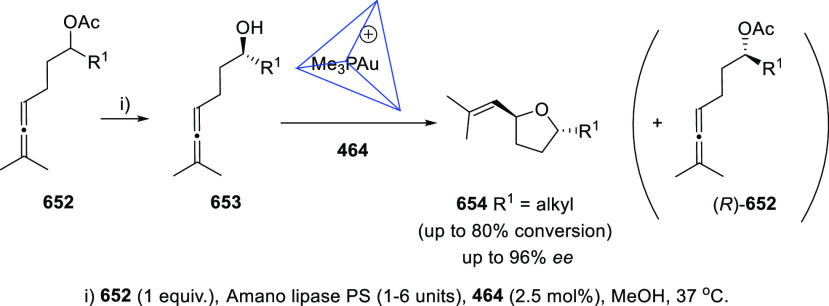
Hybrid Enzymatic/Transition Metal Catalyzed Oxycyclization of Allenols Using Supramolecular Hosts

Following their interest in supramolecular host–guest catalyzed reactions (see Scheme 80, ref (282), Bergman, Raymond, and Toste and collaborators have extended the applications of Ga_4_L_6_-encapsulated gold ions **464** in tandem reactions with biocatalysts. Nonencapsulated metal catalyst, especially gold species, can partially poison biocatalysts by binding amino-acid groups from the protein skeleton. This fact may incur in a loss of catalytic activity, unless great excess of enzyme is used. In addition, the use of supramolecular hosts exhibited many other advantages such as catalyst stabilization or aqueous media allowance. Thus, racemic acetylated γ-allenols **652** were reported to undergo KR in the presence of different enzymes such as *Amano lipase PS* providing enantioenriched allenols **653**, which after oxycyclization in the presence of supramolecular catalyst **464** yielded dihydrofurans **654** showing up to 96% *ee*. Notably, the supramolecular host–guest catalytic system **464** allowed a decrease in the enzymatic loading to six units, compared with the 25 units needed when naked Me_3_PAuCl complex was used as catalyst. Kinetic experiments supported the hypothesis of the enzyme poisoning from free metal salts, revealing no interference between encapsulated gold systems **464** and lipase enzymes (Scheme 108).^380^

## 4 Allenols in Natural Products

The diverse reactivity of allenols under different reaction conditions has been applied to the synthesis of a wide family of natural and pharmaceutically attractive products. During the past decade, several reports have appeared describing the total synthesis of naturally occurring structures incorporating allenol chemistry, normally as a key step in the whole reaction sequence. Also, natural products have been characterized and synthesized exhibiting the allenol motif in the final structure. In the first part of this section, the most significant and recent uses of allenols as key intermediates in total synthesis will be described. In a second part, allenol synthesis strategies applied to the preparation of natural product bearing an allenol unit will be detailed.

### 4.1 Allenols as Key Intermediates in Natural Product Synthesis

**Scheme 109:**
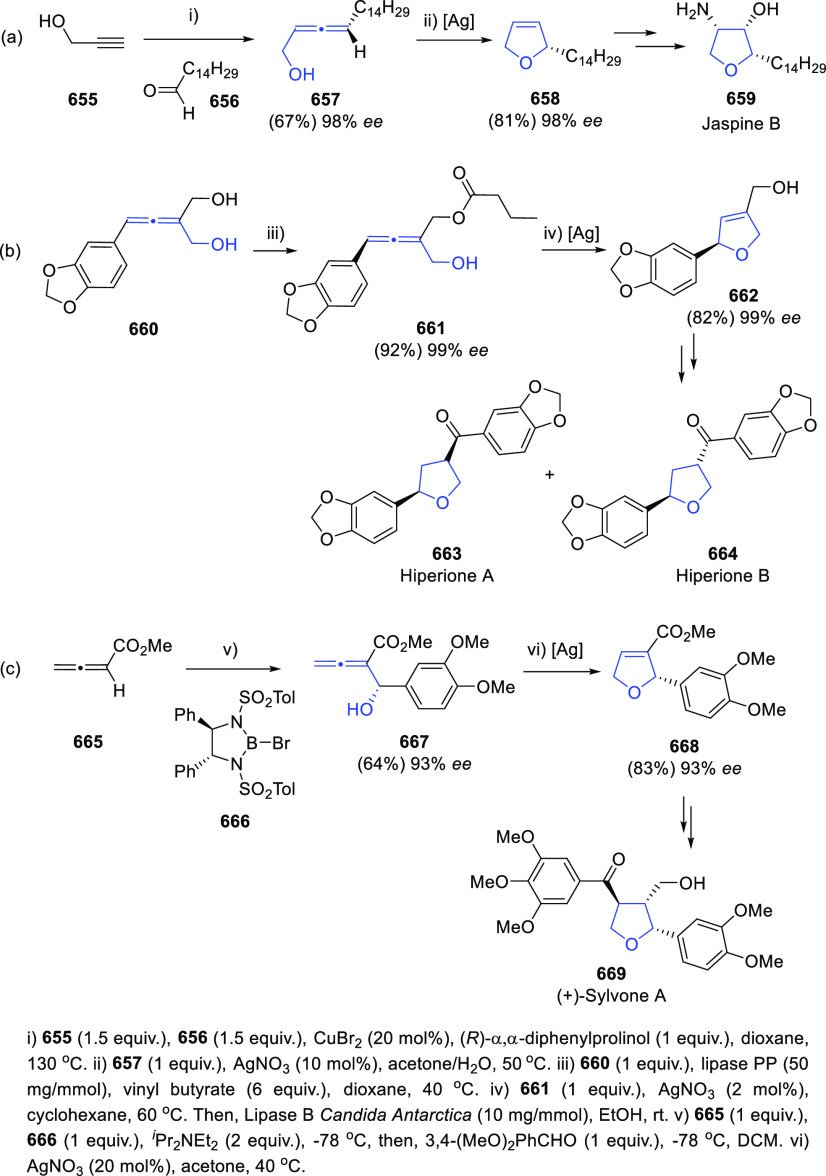
Silver-Catalyzed Oxycyclization of α-Allenols in Natural Product Synthesis

The great ability of allenes to undergo carbo- and heterocyclization reactions has been largely employed for the synthesis of the cyclic core of different naturally occurring compounds. Concretely, oxycyclization of allenols has been one the most recurring tools to get access to natural products containing 5- and 6-membered oxacycles. Silver catalysis has been frequently described to provide tetrahydrofuran systems from ennartioenriched allenols without racemization. In this context, Ballereau and co-workers have reported one short synthetic route to the natural product Jaspine B (**655**), a cytotoxic marine compound consisting in a trisubstituted tetrahydrofuran skeleton. The heterocyclic core was obtained through a silver-catalyzed *5-endo-trig* cyclization of α-allenol **657**, obtained from the enantioselective Crabbé-type reaction of propargylic alcohol (**655**) with aldehyde **656** using optically pure (*R*)-α,α-diphenylprolinol as secondary amine (Scheme 109, reaction a). Axial-to-central chirality transfer from enantioenriched allenol **657** allowed the full retention of the enantiopurity in dihydrofuran **658**. Further transformations included epoxidation, ring opening reaction in the presence of sodium azide and reduction to yield Jaspine B (**659**) in 12% overall yield through a six-step sequence.^381^

Deska et al. reported the desymmetrization of prochiral allendiol **660** using enzymatic catalysis providing enantioenriched allenol **661** exhibiting 99% *ee* (Scheme 109, reaction b). Silver-catalyzed *5-endo-trig* cyclization followed by enzymatic ester hydrolysis generated the corresponding dihydrofuran skeleton in product **662**, with no racemization observed. Compound **662** was used as precursor for the synthesis of diastereomers Hyperione A (**663**) and Hyperione B (**664**), secondary metabolites found in the leaves of *Hypericum Chinese*, showing pharmaceutical activity.^382^

(+)-Sylvone A (**669**) is a highly functionalized tetrahydrofuran metabolite principally extracted from the seeds and fruits of *piper sylvaticum* and *piper logum* plants. Yu’s research group has envisioned a synthetic sequence to yield (+)-Sylvone A, based also on a silver-catalyzed *5-endo-trig* cyclization of enantioenriched α-allenols. In this case, optically pure borane **666** was used as chiral inductor in the enantioselective aldol-type reaction of allenoate **665** with 3,4-dimethoxybenzaldehyde to yielded enantioenriched allenol **667** exhibiting 93% *ee* (Scheme 109, reaction c). Silver nitrate was then employed as the most convenient metal catalyst to promote the dihydrofuran generation from **667** to compound **668** avoiding racemization processes. Further transformations including a Michael addition provided the expected natural product (+)-Sylvone A (**669**) through a short 5-step reaction sequence.^383^

**Scheme 110:**
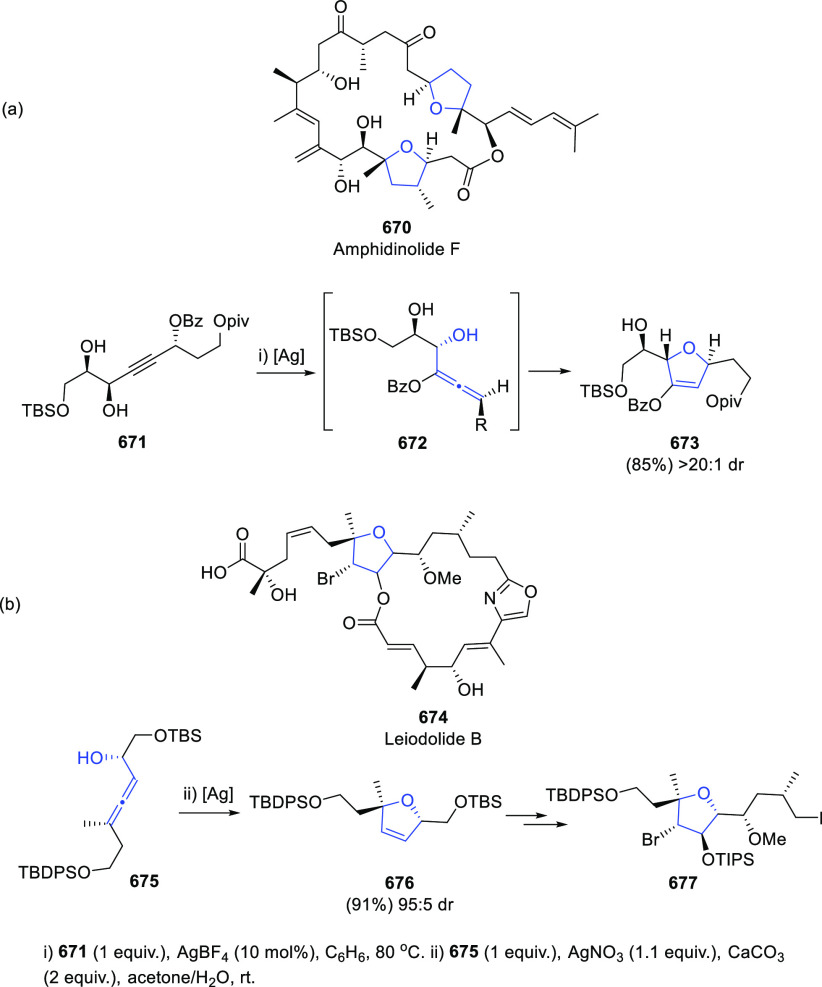
Silver-Mediated Oxycyclization of α-Allenols in the Total Synthesis of Amphidinolide F and Leiodolide B

Metal-catalyzed oxycyclization of α-allenols have also been involved in longer synthethic pathways toward the synthesis of natural products exhibiting higher structural complexity. Carter et al. have reported the total synthesis of macrolide **670**, a naturally occurring product found in *Amphidinium* sp. organisms. Compound **670** shows one of the most densely functionalized structures among the family of amphidinolides, and 11 stereogenic centers, endowing both a synthethic and analytical challenge. Also, two trans-disposed tetrahydrofuran units are present in the skeleton of compound **670**, both synthesized from optically pure alkynol **671** (Scheme 110, reaction a). In the presence of AgBF_4_ as metal salt, alkynol **671** rearranges to produce α-allenol **672** as nonisolable reaction intermediate. In situ oxycyclization of **672** generates dihydrofuran **673** in 85% yield and dr >20:1. The complete synthesis of macrolide **670** comprises 34 steps in the longest linear sequence, and its synthesis has helped to elucidate the absolute configuration of the whole structure, unresolved since its first isolation more than two decades ago.^384^

Leiodolide B metabolite (**674**) is a natural product isolated from marine sponges, showing a challenging tetrahydrofuran unit bearing four stereogenic centers on its northern fragment (Scheme 110, reaction b). Fürstner and collaborators have envisioned a total synthesis of compound **674** through a 26-step reaction sequence. To achieve the tetrahydrofuran structure with the appropriate stereochemistry, a silver-promoted *5-endo-trig* oxycyclization of enantioenriched α-allenol **675** was proposed. In this manner, dihydrofuran **676** was therefore achieved in high 91% yield and further converted into tetrahydrofuran **677**. Further transformations allowed the preparation of macrolide **674**, although full charecterization and interpretation of the naturally isolated analogous remains unresolved, leaving the quest for the Leiodolide B absolute configuration still open.^385^

**Scheme 111:**
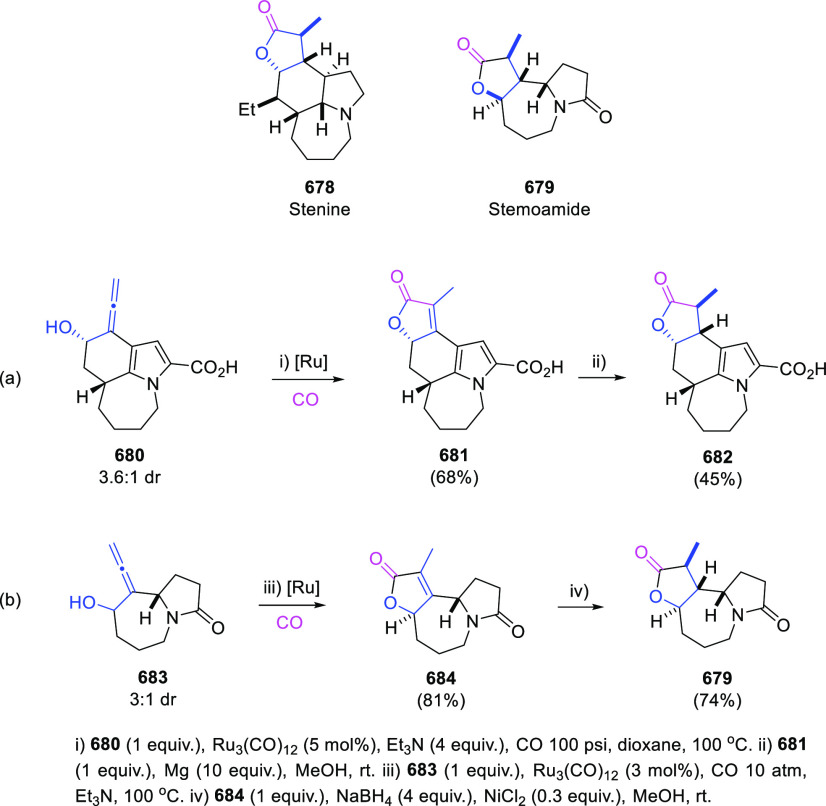
Ruthenium-Catalyzed Carbonylation of α-Allenols toward the Synthesis of the γ-Butyrolactone Scaffold in Natural Products

The γ-butyrolactone scaffold is ubiquitous in nature and present in different biologically active alkaloids. Stenine (**578**) and Stemoamide (**579**), two naturally occurring heterocycles from the stemona alkaloid family, exhibit a γ-butyrolactone unit which has been achieved trough a ruthenium-catalyzed carbonylation of allenols. In both cases, related exocyclic allenols **580** and **583** were synthesized and submitted to ruthenium catalysis under CO atmosphere to yield butenolide systems **581** and **584** (Scheme 111). In the final steps of the synthesis of the stenine and stemoamide cores, double bond reduction by treatment with Mg/MeOH and nickel boride, respectively, led to the desired compounds **682** and **679**.^386,387^

**Scheme 112:**
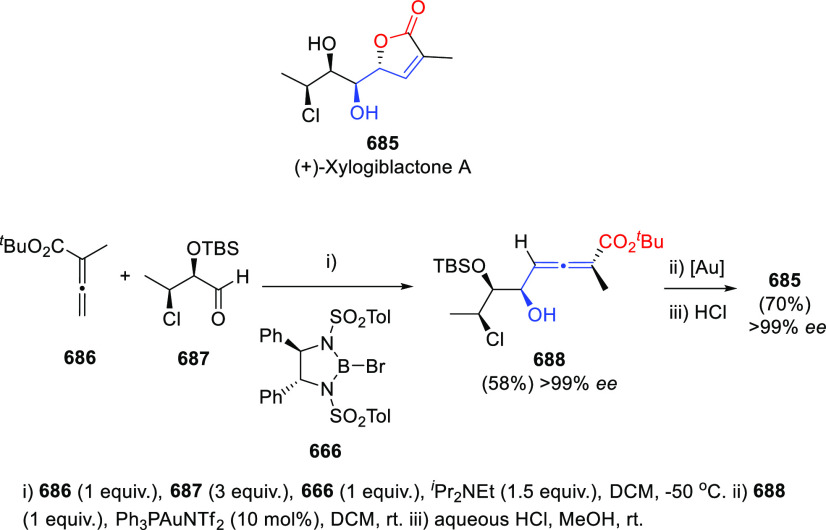
Synthesis of (+)-Xylogiblactone A through Gold-Catalyzed Cycloisomerization of Enantioenriched Allenols

In a recent total synthesis of (+)-Xilogyblactone A (**685**), an alternative gold-based methodology has been introduced to access the butanolide motif. *^t^*Butyl allenoate **686** reacted through an asymmetric aldol-type transformation with aldehyde **687** in the presence of enantiopure organoboron reagent **666**. Enantioenriched α-allenol **688** was therefore synthesized exhibiting >99% *ee*. Interestingly, gold treatment of allenol **688** yielded the butenolide skeleton from selective nucleophilic attack of the carboxylic oxygen to the allene moiety. (+)-Xilogyblactone (**685**) was obtained after acidic hydrolysis of the TBSO group through a short three-step sequence and a 41% overall yield (Scheme 112).^388^ A related gold-catalyzed cycloisomerization of allenyl carboxylates has also provided the γ-butyrolactone unit in the total synthesis of Xestospongienes E, F, G, and H.^389^

**Scheme 113:**
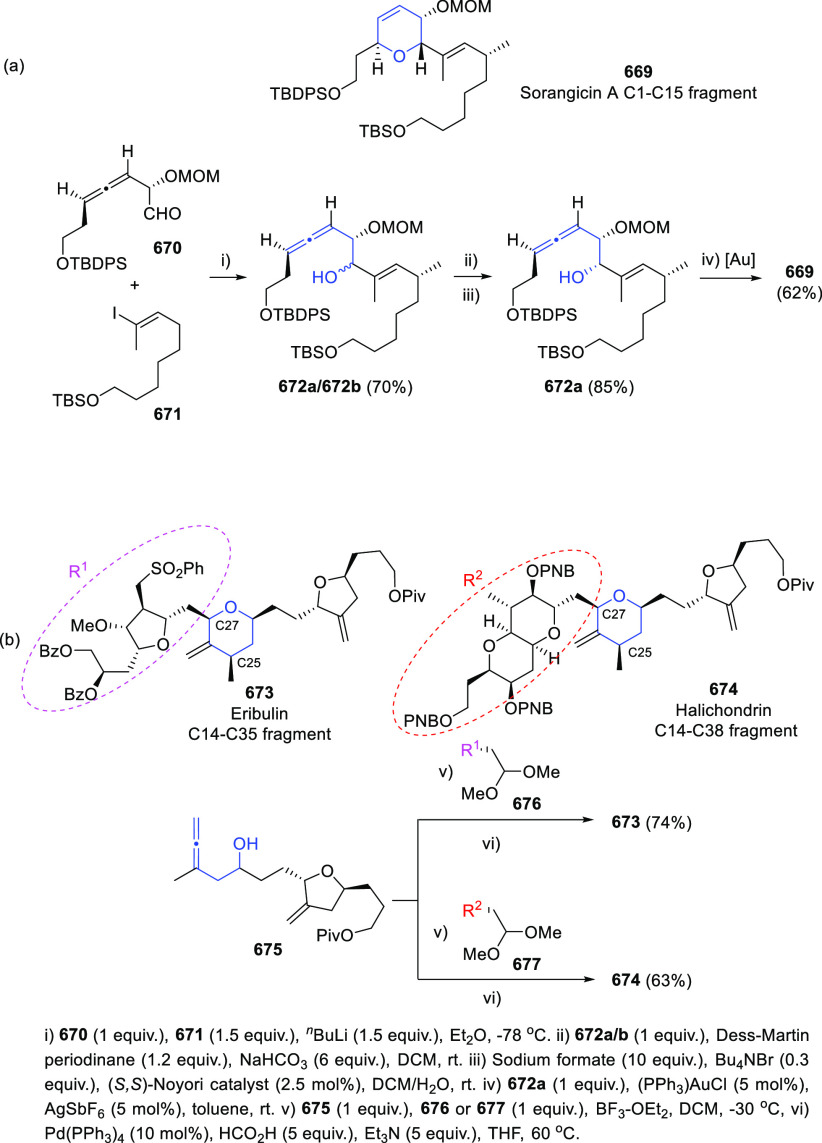
Synthesis of the Dihydropyran and Tetrahydropyran Fragments of Sorangicin A, Eribulin, and Halichondrin

Dihydropyran and tetrahydropyran fragments found in natural products have been accessed from oxycyclization of β-allenols through diverse strategies. The dihydropyran C1–C15 subunit **689** of Sorangicin A, a potent antibiotic isolated from *Sorangium Cellulosumi* bacteria, has been synthesized through the gold-catalyzed *6-endo-trig* cyclization of enantioenriched β-allenol **692a**, prepared from oxidation and further asymmetric reduction of the diastereomeric mixture **692***a***/692b** (Scheme 113, reaction a).^390^

Prins-type cyclization of β-allenol **695** with dimethyl acetals **696** and **697** followed by Tsuji reduction led to the C14–C35 fragment of Eribulin (**693**) and the C14–C38 fragment of Halichondrin (**694**), respectively, two macrolides of marine origin exhibiting potent antitumor activity. Chirality transfer from enantioenriched allenols and acetals led to the stereocontrolled generation of the C27 sterocenter, and further Tsuji reduction under palladium conditions provided the stereodefined C25 center in both fragments **693** and **694** (Scheme 113, reaction b).^391^

**Scheme 114:**
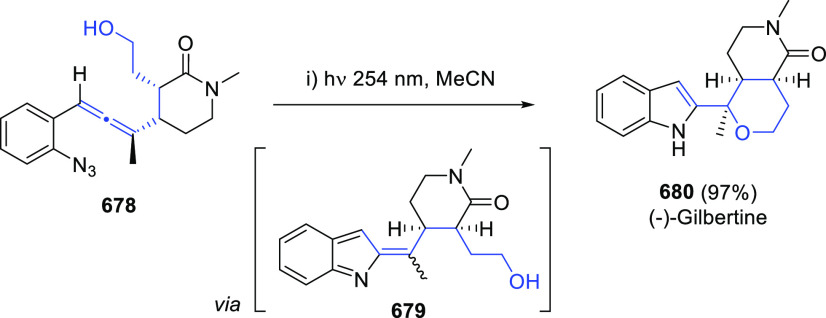
Synthesis of (−)-Gilbertine through Photoinduced Cyclization of Azido-allenols

A more intrincate reaction mechanism was envisioned for the synthesis of (−)-Gilbertine natural product (**680**), a member of the uleine alkaloid family. Allenyl azide **678** undergoes photoinduced azacyclization to yield indolidene intermediate **679**, which after *6-exo-trig* oxycyclization reaction from the hydroxyl group yields the fused tetrahydropyran skeleton, favored by the formation of the more stable indol aromatic ring in compound **680** (Scheme 114).^392^

In the context of allenol oxycyclization reactions toward the synthesis of naturally occurring products, related allenyl hydroxylamines have been described to undergo *in situ* cycloisomerization to yield polycyclic oxazines en route to the total synthesis of Casuarine, Australine, and diverse non-natural derivatives.^393^

**Scheme 115:**
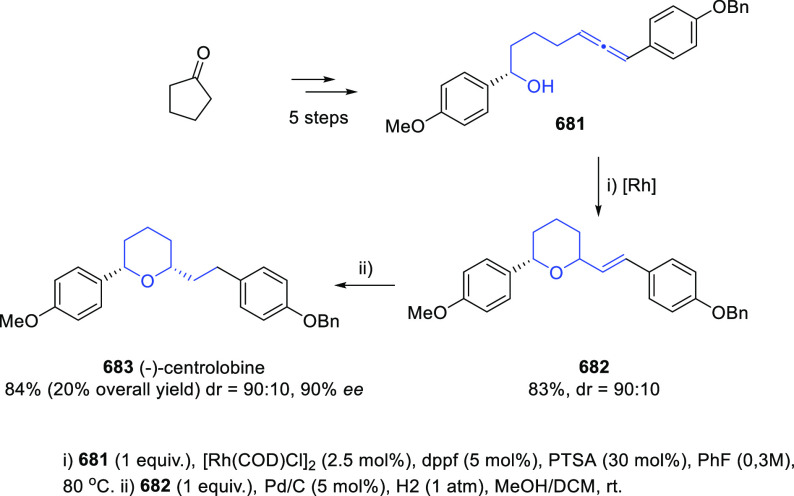
Synthesis of (−)-Centrolobine Natural Product from an Enantioenriched Internal Allenol

Breit and co-workers have recently reported a diasteroselective synthesis of dihydropyrans through the rhodium-catalyzed oxycyclization of both terminal and internal allenols, using dppf as ligand. The methodology has been successfully applied to the synthesis of (−)-centrolobine (**683**) through a six-steps reaction sequence and 20% overall yield (Scheme 115).^394^

**Scheme 116:**
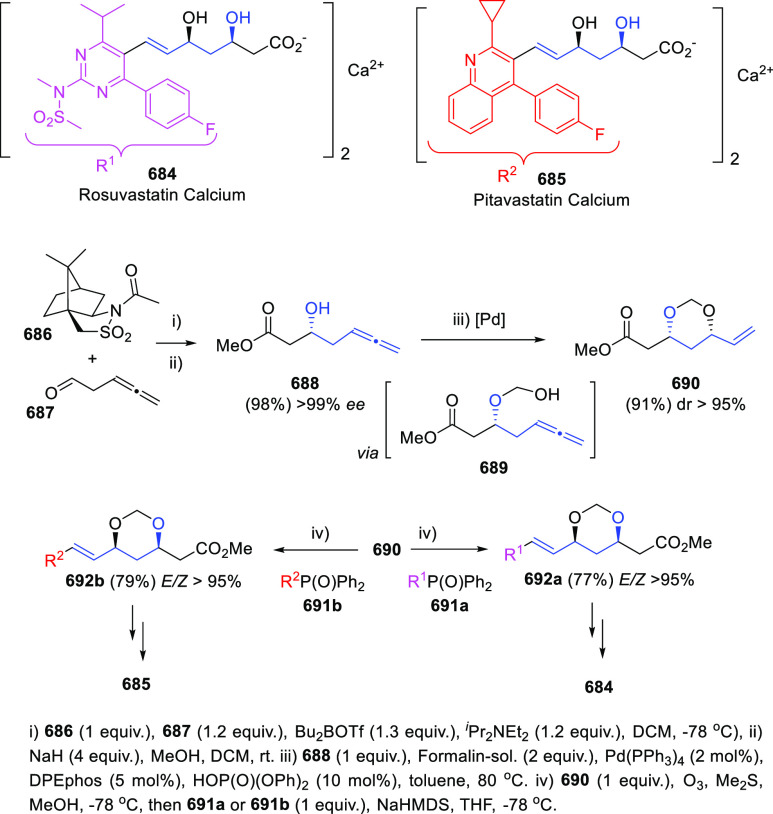
Synthesis of Rosuvastatin and Pitavastatin Natural Products from an Enantiopure β-Allenol

The β-allenol scaffold has been also presented as precursor of acyclic fragments. *Syn*-1,3-diol is a common motif in every compound of the statin family. Breit and collaborators have developed the diasteroselective synthesis of *syn*-dioxanes **690** from *in situ* generated allenyl hemiacetals **689** as *syn*-1,3-diol precursors (Scheme 116). The asymmetric version of this transformation was achieved using acetylsultam borylenolate **686** as chiral inductor. Coupling reaction of compound **686** with allenyl carbaldehyde **687** followed by amide hydrolysis led to enantiopure β-allenol **688**. Further coupling reaction of *syn*-dioxanes **690** with the appropriate phosphoryl compound **691** and acetal hydrolysis allowed the total synthesis of Rosuvastatin (**684**) and Pitavastatin (**685**).^395^

**Scheme 117:**
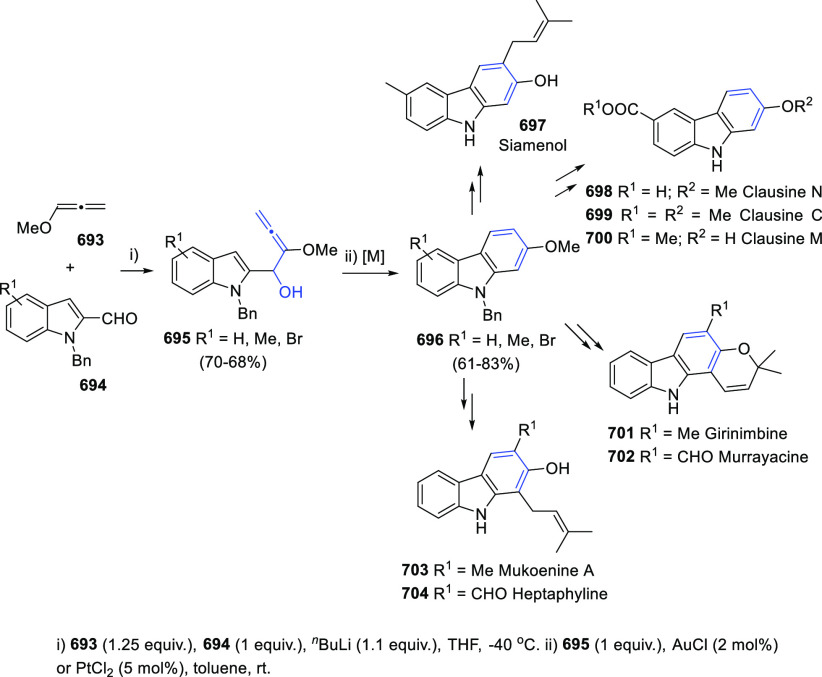
Synthesis of Carbazole Alkaloids from Indole-Tethered Allenols

As previously mentioned in section 3.1.2, tandem carbocyclization/dehydratation reactions of allenols constitute straightforward procedures for the preparation of aromatic and heteroaromatic polycyclic structures. Ma and co-workers have employed this strategy for the synthesis of a wide variety of alkaloids from the carbazole family, starting from readily available methoxypropadiene (**693**) and indole-2-carbaldehydes **694**. Treatment of methoxypropadiene (**693**) with *^n^*BuLi in the presence of indole-2- carbaldehydes **694** led to indole-tethered allenols **695** (Scheme 117). Carbocyclization of compounds **695** under precious metal catalyzed conditions followed by *in situ* dehydratation provided 2-methoxy-3-methylcarbazoles **696** in high yields. Carbazoles **696** were employed as key intermediates for the synthesis through short reaction sequences of natural occurring alkaloids such as Siamenol (**697**) or the Clausine family drugs (**698**–**700**), both exhibiting promising anti-HIV activities and commonly used in traditional medicine. Also, Girinimbine (**701**), Murrayacine (**702**), or Mukoenine-type structures (**703**, **704**), showing cytotoxic activity against a wide variety of cell lines were obtained.^232^

**Scheme 118:**
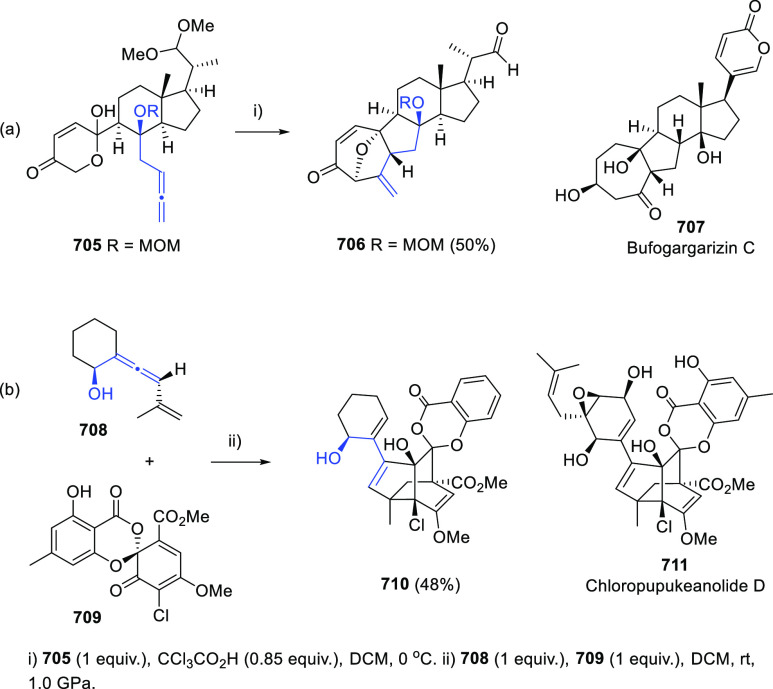
Synthesis of Natural Products from Allenol Cycloaddition Processes

Cycloaddition reactions involving allenol molecules en route to natural products and fragments have also been described. Early examples deal with the Diels-Alder of allenols and methyl propiolate in the total synthesis of Quassin.^396^ More recent advances include the intramolecular (5 + 2) cycloaddition of allenol **705** for the synthesis of the tetracyclic core of Bufogargarizin C (**707**) (Scheme 118, reaction a),^397^ or the tandem Diels-Alder/carbonyl-ene reaction from allenol **708** to provide the Chloropupukeananin D analogous **710** (Scheme 118, reaction b).^398^ The first and asymmetric total synthesis of the bioactive bufospirostenin A, an unusual spirostanol natural product, has been accomplished taking advantage of the intramolecular allenic Pauson–Khand reaction of an alkyne-tethered allenol for the construction of a tetracyclic skeleton.^399^

**Scheme 119:**
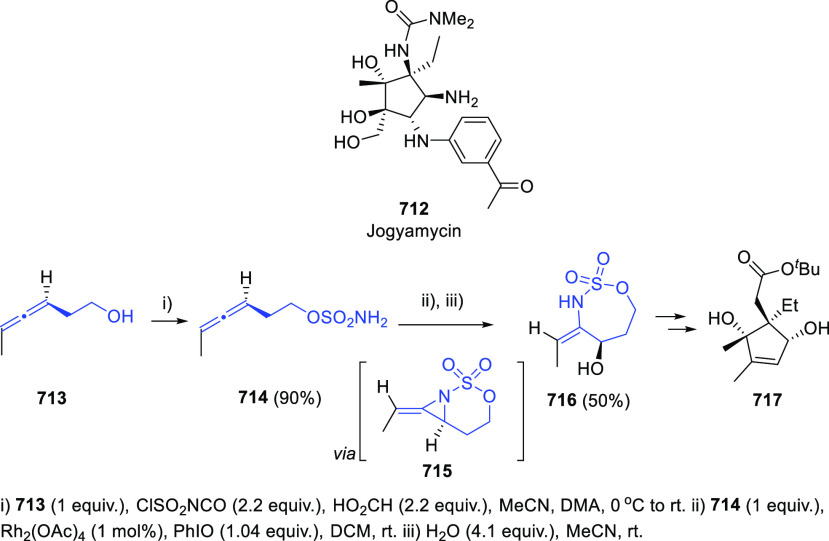
Formal Synthesis of Jogyamycin through Rhodium-Catalyzed Azacyclization of a β-Allenol

Jogyamicin (**712**) is an aminocyclopentitol-based natural product recently isolated from *Streptomyces* culture broth. Its potent antiprotozoal activity along with its challenging structure has attracted the interest of diverse research groups. One recent approach to the five-membered core of Jogyamicin starts from enantioenriched β-allenol **713**, which after protection as allenic sulfamate **714**, followed by oxidative allene amination under rhodium catalysis led to cyclic sulfamate **716** through aziridine-intermediate **715** (Scheme 119). A 15-step reaction sequence from sulfamate **716** provided the pentacyclic structure **717** in a 6% overall yield, a known key intermediate in the total synthesis of Jogyamicin.^400^

**Scheme 120:**
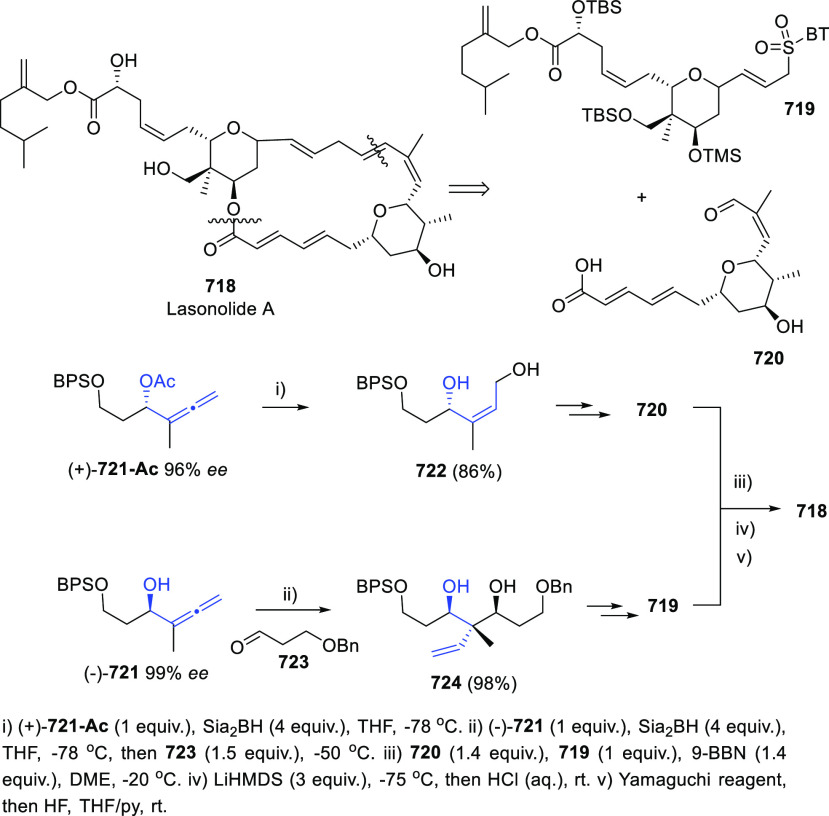
Total Synthesis of Lasonolide A from Enantioenriched α-Allenols

Palladium-catalyzed additions and hydroborations of allenes has been applied to the preparation of different natural products. Yoshida’s research group has described the synthesis of sesquiterpenes (−)-HM-3 and (−)-HM-4 based on the palladium-catalyzed addition of boronic acids to α-allenols,^401^ and the synthesis of enokipodins A and B, two sesquiterpenoids from the α-cuparenone family exhibiting antimicrobial activity through a similar strategy.^402^ Roulland and co-workers have reported the total synthesis of the antibiotic Tiacumicin B incorporating a palladium-mediated cross-coupling reaction of alkynes and allenols.^403^ Hong and collaborators have envisioned a total synthesis of Lasonolide A (**718**), a natural product from marine origin and promising activity in pancreatic cancer therapies. The proposed retrosynthesis disconnects the macrolide product in fragments **719** and **720**, prepared from the hydroboration of both allenes (+)-**721-Ac** and (−)-**721**, after a 12- and 11-step sequence, respectively. Enantiopure allenes (+)-**721-Ac** and (−)-**721** have been prepared taking advantage of the enzymatic resolution of racemic allenol **721**. Julia-type olefination of fragments **719** and **720** followed by Yamaguchi macrolactonization and total desilylation provided the expected structure of the Lasonolide A polyketide (Scheme 120).^404^

**Scheme 121:**
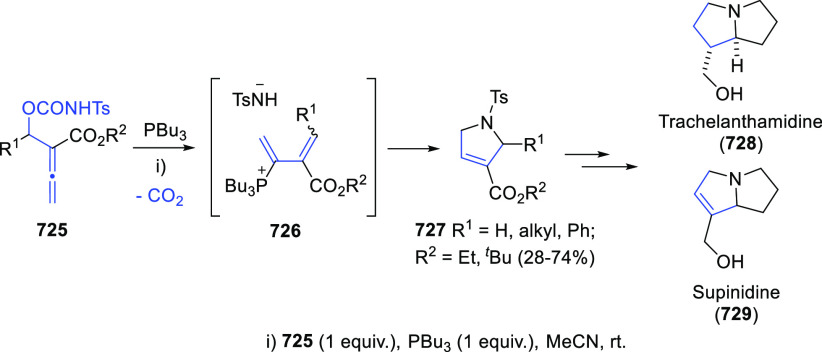
Total Synthesis of Trachelanthamidine and Supinidine

Recently, the ability of allenyl carbamates, readily available from the corresponding allenols, to generate dienes as reaction intermediates has been employed in the total synthesis of trachelanthamidine and supinidine through a (4 + 1) ring closing process. Thus, allenyl carbamates **725** reacted under phosphine-promoted conditions to yield pyrrolines **727**, key reaction intermediates in the total synthesis of pyrrolizidine alkaloids **728** and **729** (Scheme 121).^405^

### 4.2 Natural Products Bearing the Allenol Motif

**Scheme 122:**
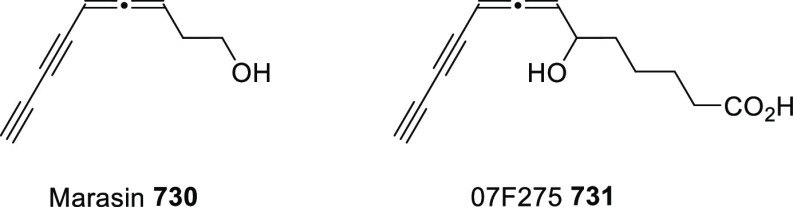
Linear Allenols Exhibiting Antibiotic Properties

Once considered chemical curiosities and extremely reactive compounds, allenes are currently found in more than 150 thermally and photochemically stable natural products.^406^ Despite the allenol system is infrequent in naturally occurring systems, and linear allenes commonly show chemical instability, some simple linear molecules bearing the allenol motif have exhibited important antibiotic activity, such as the diyonic compounds marasin (**730**),^407^ and 07F275 molecule (**731**),^408^ both described in the late 80s (Scheme 122).

**Scheme 123:**
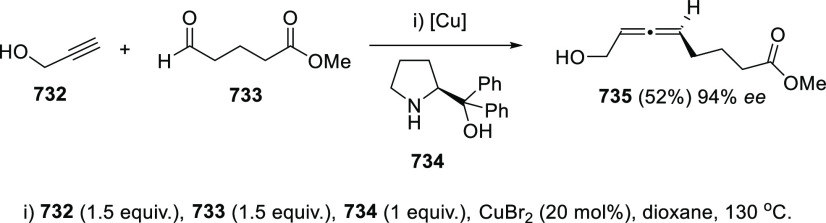
Enantioselective Allenation of Terminal Alkynes for the Synthesis of Naturally Occurring Allenol Structure

During the past decade, some examples describing naturally occurring linear allenes have been reported. Ma’s research group has applied their chiral amine enantioselective allenation of terminal alkynes for the one-step synthesis of (*R*)-8-hydroxyocta-5,6-dienoate (**735**), a potent antifungal and antibiotic molecule extracted from the Japanese tallow tree *Sapium japonicum*. Reaction of propargylic alcohol (**732**) and methyl-5-oxopentanoate **733** in the presence of (*S*)-diphenyl(pyrrolidin-2-yl)methanol **734** and CuBr_2_ as metal catalyst led to the expected 1,3-disubstituted allene moiety in compound **735** (Scheme 123). Efficient chirality transfer from optically pure secondary amine **734** allowed the obtention of allenol **735** in 94% *ee*.^389^

**Scheme 124:**
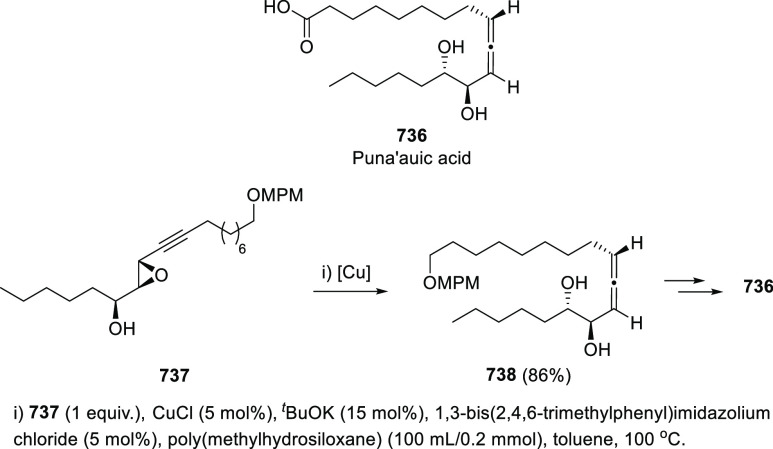
Conjugated Hydride Addition to Epoxy Alkynes Towards the Total Synthesis of Allenol-Based Puna’auic Fatty Acid

**Scheme 125:**
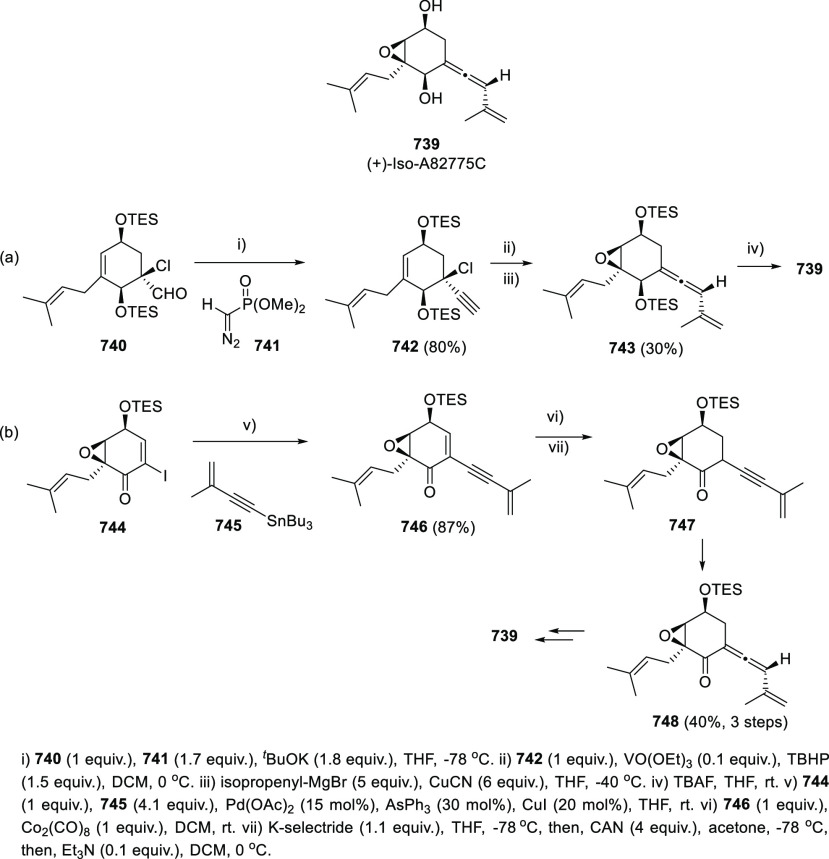
Alternative Approaches to the Allenic Moiety Generation in (+)-Iso-A82775C Total Synthesis

Thomas and collaborators have reported the total synthesis of the allenol-based natural product Puna’auic acid (**736**), a fatty acid isolated from marine cyanobacterium. The allenol motif is generated in one of the latter steps of the reaction sequence, through a copper-catalyzed conjugated hydride addition to enantioenriched epoxy alkyne **737**, following Krause’s procedure.^409^ Although the allene biosynthethic origins are not yet fully understood, the discovery of minor alkyne metabolites related to structure **736** from the same natural sources point to a similar conjugated hydride addition as the most plausible biosynthethic route (Scheme 124).^410^

(+)-Iso-A82775C natural product (**739**) has been isolated from the fermentation culture of fungus *Pestalotiopsis fici*, and it has been proposed as a biosynthethic intermediate of the previously mentioned chloropupukeananin family. Compound **739** is a polysubstituted cyclohexane ring bearing an exocyclic allene. Its structure and complex stereochemistry have attracted the interest of different research groups, reporting alternative synthethic strategies. Suzuki and Tanino and co-workers have proposed a Seyferth–Gilbert homologation of carbaldehyde **740** to generate ethynyl cyclohexane structure **742** (Scheme 125, reaction a). Epoxidation of the endocyclic olefin followed by Cu-mediated anti-S_N_2′ reaction of the chloroalkyne with isopropenyl magnesium bromide provided adduct **743** exhibiting the allene moiety with the adequate stereochemistry. Natural product **739** was obtained after desilylation of protected alcohols with TBAF.^411^ Han et al. have envisioned a different approach to get access to the allene moiety in **739**. Stille coupling of iodo vinyl derivative **744** and organostannane **745** led to the dienyne **746** (Scheme 125, reaction b). Selective endocyclic double bond reduction with K-selectride yielded cyclohexanone **747**, which suffered enyne-enallene isomerization in the presence of catalytic amounts of triethylamine. Total synthesis of **739** was completed with further carbonyl reduction and desylylation steps.^412^

**Scheme 126:**
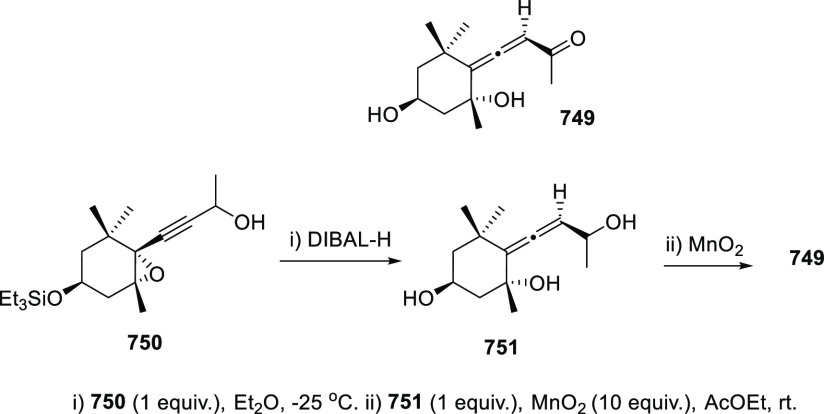
Synthesis of the Grasshopper Ketone

Carotenoids represent the largest group of natural products exhibiting the allenol motif, being the Grasshopper ketone (**749**) one of the most commonly reported. First synthesis and isolation date to the late 60s,^413^ pointing to a dietary metabolism of larger carotenoids as the most plausible biological origin of compound **749**. Eugster et al. described a total synthesis of allenol **749** based on a S_N_2′ hydride addition onto propargylic oxirane precursor **750**, followed by selective oxidation under MnO_2_ conditions (Scheme 126).^414^

**Scheme 127:**
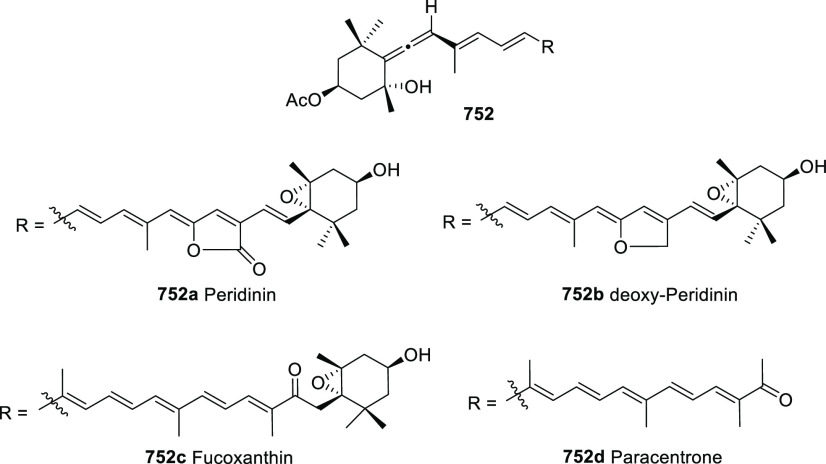
Allenol-Based Natural Products from the Norcarotenoid Family

A related exocyclic allene unit is present in the wide family of xanthophyll norcarotenoids, naturally occurring compounds isolated from marine microalgae. Great effort has been made during the last years to provide an efficient synthethic route to some of these carotenoid molecules such as the most abundant Peridinin (**752a**), Fucoxanthin (**752c**), the biosynthetic intermediate Paracentrone (**752d**), and diverse natural and non-natural derivatives (Scheme 127).

**Scheme 128:**
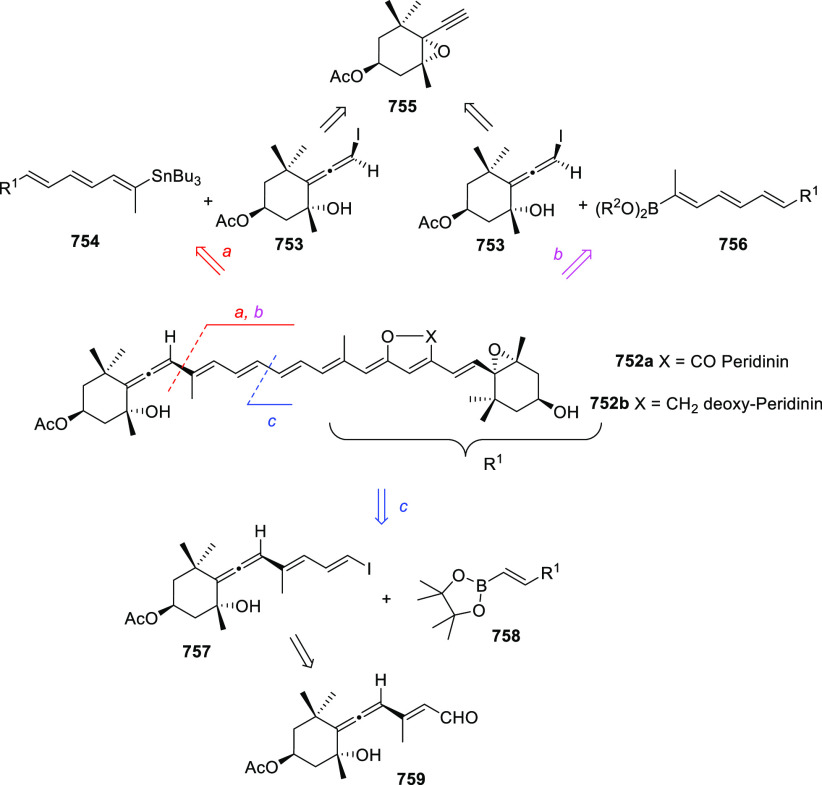
Retrosynthethic Strategies for the Synthesis of Peridinin and Deoxy-Peridinin Natural Products

Álvarez and de Lera et al. have proposed a retrosynthethic analysis for the total synthesis of Peridinin based on Julia-Kocienski olefinations, and a Stille coupling reaction to incorporate the allenic moiety from fragment **753** (Scheme 128, path a). Also, in depth investigations on the stereoselective oxidative addition or S_N_2′ substitution of palladium reagents to iodoallene derivatives **753** were reported. Iodoallene **753** was prepared from the corresponding alkyne **755**.^415−417^ Burke’s research group has devised a Suzuki coupling of iodoallene **753** and the corresponding boronic acids **756** to incorporate the allenic fragment utilizing the same disconnection strategy (Scheme 128, path b).^418,419^ A different retrosynthetic approach for the total synthesis of analogous deoxy-Peridinin (**752b**) has been proposed by Sakaguchi and Katsumara and collaborators. Despite this strategy being Suzuki-based, the novelty lays on a different disconnection, which results in allenic fragment **757** and boronic acids **758** (Scheme 128, path c).^420^ Wittig olefination from previously known enallenal **759** provided the dienallenyl iodide **757**.^421^

**Scheme 129:**
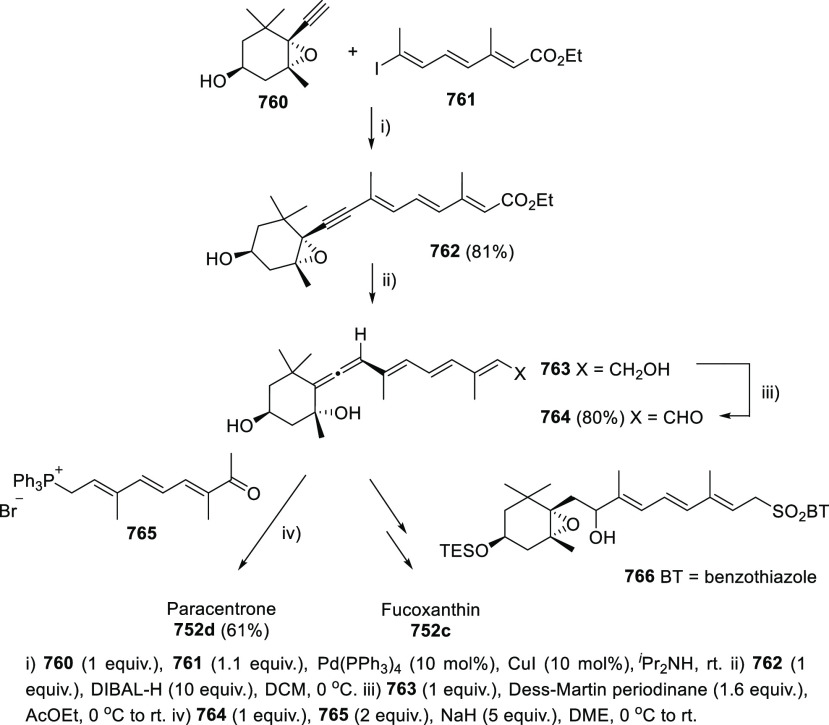
Synthesis of Paracentrone and Fucoxanthin Natural Products

Katsumara’s research group has proposed a total synthesis for both Fucoxanthin (**752c**) and Paracentrone (**752d**) natural products following similar strategies. Sonogashira coupling of ethynyl epoxide **760** and iodo triene **761** yielded trienynyl carboxylate **762** (Scheme 129). Next, treatment with DIBAL-H as hydride source smoothly rearranged the ethynyl epoxide moiety to generate the allenol motif and also reduced the carboxylate unit to the corresponding terminal alcohol building compound **763**. Further Dess-Martin oxidation followed by Wittig olefination in the presence of phosphonium salt **765** provided the Paracentrone skeleton (**752d**) (Scheme 129, left).^422^ A Suzuki alternative for the Wittig olefination in the last steps of the synthesis has also been stated,^423^ also allowing the synthesis of related 19-hexanoyloxyparacentrone 3-acetate.^424^ Likewise, Fucoxanthin (**752c**) total synthesis has been achieved through a Julia-type olefination of the allenic fragment **764** with hydroxysulfone **766** (Scheme 129, right).^425^

**Scheme 130:**
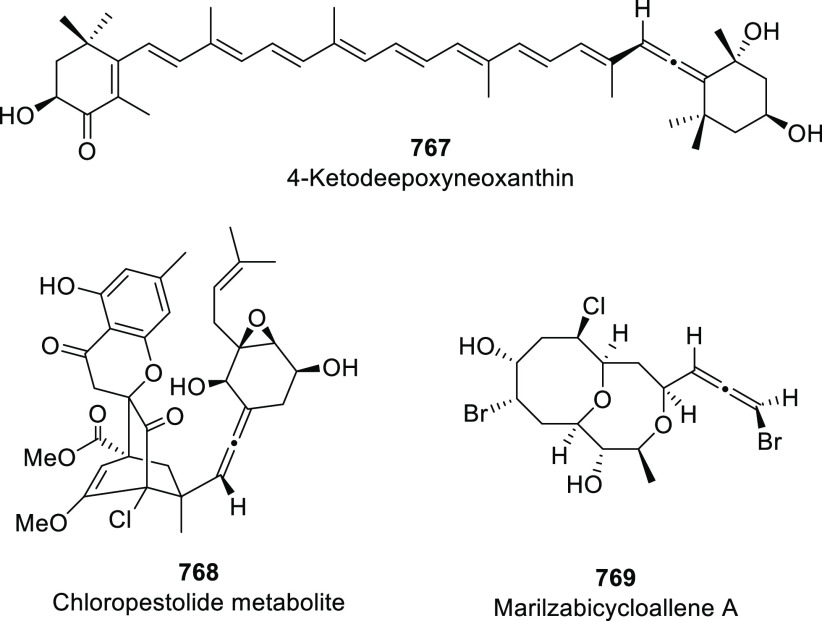
Proposed Structure of Allenol-Containing Natural Products As Determined by NMR Investigations

Besides the above-mentioned synthethic approaches to naturally occurring targets, the determination of both structure and absolute configuration of allenol-containing natural products have been reported. Thus, Maoka et al. have stated the absolute configuration of minor carotenoid 4-Ketodeepoxyneoxanthin (**767**) in basis of NMR investigations.^426^ Che’s and Souto’s research groups have respectively proposed the full structure and stereochemistry of Chloropestolide metabolite **768**,^427^ and Marilzabicycloallene A (**769**), exhibiting an unusual bromoallene motif (Scheme 130).^428^

**Scheme 131:**
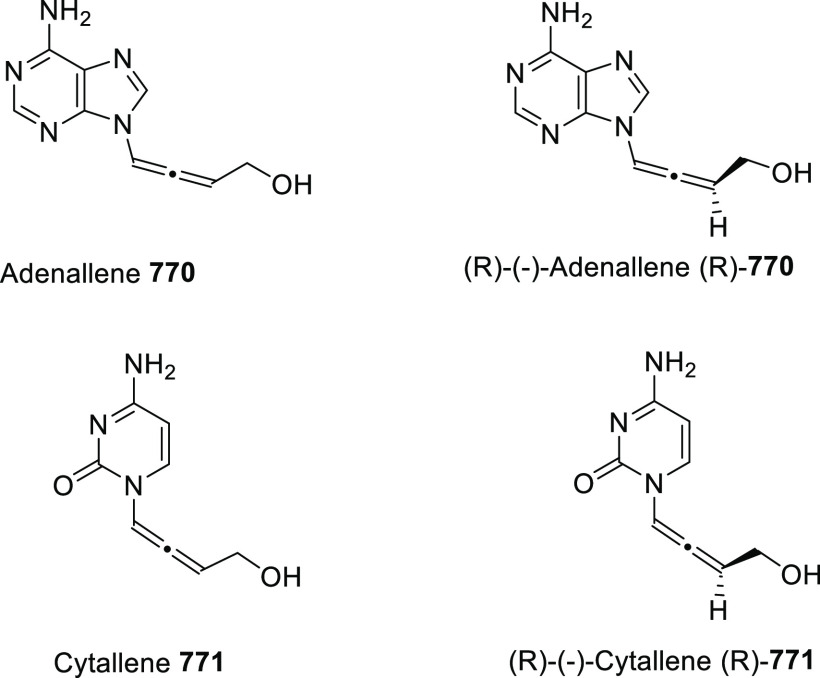
Allenol Motifs in Nucleoside Analogous with Pharmaceutical Activities

The diverse and intriguing biological properties of naturally occurring allenols described so far, have paved the way to the synthesis of non-natural analogous and the examination of their pharmaceutical activities. Zemlicka has recently reported a critical comparison of the antiviral properties of a wide family of lipophilic nucleoside analogous and their phosphoramidates, including several examples of allenol-containing systems.^429^ For instance, adenosine- and cytosine-based compounds **770** and **771** have been prepared through basic equilibration from the corresponding alkynol precursors. Both molecules exhibit potent cytotoxic and antiviral activities. Interestingly, anti-HIV properties of **770** and **771** were found to be in close dependency of the absolute configuration of the allene moiety, being the (R)-isomer obtained through enzymatic resolution the active species (Scheme 131).^430,431^

More non-natural pharmacologically attractive allenols were described and reviewed during the 80s and 90s decades such as allenol-based prostaglandin and carbacyclin systems,^432^ allenic amino acids bearing hydroxyl groups,^433^ and allenic steroids.^434^

## 5 Conclusions

Allenol chemistry remains to be a hot research topic, which includes two main areas, namely the development of new methods for their synthesis and the discovery of novel and fascinating reactivity, which converts the allenol moiety in a powerful building block in the modern synthetic arsenal. Allenes decorated with hydroxyl units, namely allenols, exhibit unique and particular reactivity compared to the nonsubstituted analogous. The allenol reactivity could be classified in three main categories: (i) Allenols reacting as π-activated alcohols, where the hydroxyl group leaves the molecule or undergoes 1,3-migration rearrangement processes, leading to open-chained systems such as dienes or enynes, or to aromatic cyclic structures. (ii) Those reacting as bidentate nucleophiles-electrophiles, taking advantage of both the hydroxyl nucleophilicity as well as the allene electrophilicity when π-metal activation takes place. Oxacyclic structures such as furans or pyrans are accessed. (iii) Those where the hydroxyl group assists any kind of allene transformation, frequently by metal intermediate coordination. Also, the frequent use of allenols as key intermediates for the total synthesis of natural product deserves to be mentioned.

The extensive use of allenols as synthetic intermediates is associated with the implementation of an increasing number of methodologies for their preparation, both in racemic and enantiopure manner. Particularly attractive is the use of modern catalytic methods for the synthesis of enantioenriched allenols, which can display astonishing axial and central chirality. Last but not least, despite that the allenol scaffold is not commonly encountered in Nature, several allenol-based natural products have been isolated, characterized and synthesized. All contributions together support the widespread use and importance of the allenol functionality in current organic chemistry.

The intriguing reactivity so far exhibited by the allenol functional group, and the wide range of structures accessible from allenol starting materials will certainly inspire organic chemist to pursuit new advances and results that will be shortly coming in this area. In one hand, allenol-containing molecules constitute an ideal playground to continue the development of modern synthetic methodologies, as it has been recently illustrated by the recent micellar catalysis or the gold-based supramolecular catalysis, both of them based on allenol oxycyclization reactions. The field is dominated by the use of catalysts derived from expensive transition metals such as gold and palladium, with punctual incorporation of other metals. It should be desirable the widespread use of inexpensive and more environmentally friendly metals such as iron, copper, etc. Besides, despite the appearance of several catalytic protocols in heterogeneous phase through the use of metal nanoparticles, the more of the reactions are performed in homogeneous conditions. On the other hand, covalent–organic frameworks (COFs) and metal–organic frameworks (MOFs) have attracted considerable interest in recent years, but its application in allenol chemistry remains elusive. Consequently, more sustainable processes are desirable. In this context, the incorporation of recent progresses in photochemical methods and modern electrochemistry persist as a challenge. The application of photochemistry in allenol chemistry is restricted to a couple of isolated reports dealing with photoredox catalysis while there is absence of information concerning electrochemical methods. The use of enzymes in allenol chemistry is limited to the classical use for the resolution of racemic mixtures, but an efficient use of bioengineering advances should be taken into account. Besides, ongoing endeavors to discover competent asymmetric routes are largely based on designing and building new and exotic chiral nonracemic ligands or catalysts; however, the recognition of conveniently activated allenol precursors to enlarge catalytic effectiveness is critical too. On the other hand, the potential axial chirality of the allene motif is still unexploited, being the axial-to-central chirality transfer processes from axially enantioenriched 1,2-dienes one of the most notable challenges regarding the chemistry of allenes. Also, the inexhaustible search of new and more potent drugs, and the synthesis and characterization of yet unreported natural products often bearing oxacyclic moieties, will be certainly supported by the rich and efficient chemistry of the allenol system.

## Abbreviations

Acac: acetyl acetonate
BINAP: 2,2′-bis(diphenylphosphino)-1,1′-binaphthalene
Bn: benzyl
Boc: *tert*-butyloxycarbonyl
Bpy: 2,2′-bipyridine
BPS: 4,4′-sulfonyldiphenol
BQ: benzoquinone
BT: benzothiazole
Bz: benzoyl
Cbz: benzyloxycarbonyl
Cod: cyclooctadiene
CPME: cyclopentyl methyl ether
CpMe_4_: tetramethylciclopentadienyl
Cy: cyclohexyl
DABCO: 1,4-diazabicyclo[2.2.2]octane
Dba: dibenzylacetone
DCE: dichloroethane
DCM: dichloromethane
DDQ: 2,3-dichloro-5,6-dicyano-1,4-benzoquinone
DEAD: diethyl azodicarboxylate
DIBAL-H: diisobutylaluminum hydride
DMAP: 4-dimethylaminopyridine
DMF: dimethylformamide
DMSO: dimethyl sulfoxide
DPEphos: bis[(2-diphenylphosphino)phenyl] ether
Dppf: 1,1′-Ferrocenediyl-bis(diphenylphosphine
DTBM-SEGPHOS: 5,5′-bis[di(3,5-di- *tert*-butyl-4-methoxyphenyl)phosphino]-4,4′-bi-1,3-benzodioxole
HMDS: hexamethyldisilazane
HMPA: hexamethylphosphoramide
LDA: lithium diisopropyl amine
m-CPBA: *meta*-chloroperbenzoic acid
MOM: methoxymethyl
Ms: methanesulfonyl chloride
NBS: *N*-bromosuccinimide
NCS: *N*-chlorosuccinimide
NFSI: *N*-fluorobenzenesulfonimide
NP: nanoparticles
NPSP: *N*-(phenylseleno)phthalimide
PCC: pyridinium chlorochromate
Pin: pinacol
PINAP: (2-phosphino-1-naphthyl)phthalazinamine
Piv: pivaloyl
PMP: *p*-methoxyphenyl
PNB: *p*-nitrobenzoyl
PPL: lipase porcine pancreas
PTSA: *p*-toluenesulfonic acid
Py: pyridine
Rh_2_(S-DOSP)_4_: dirhodium tetrakis((*S*)-*N*-(dodecylbenzenesulfonyl)prolinate)
Sia_2_BH: disiamylborane
TBAB: tetrabutylammonium bromide
TBAI: tetrabutylammonium iodide
TBDPS: *tert*-butyldiphenylsilyl
TBHP: *tert*-butyl hydroperoxide
TBS: *tert*-butyl dimethylsilyl
TDMPP: tris(2,6-dimethoxyphenyl)phosphine
TEA: triethylamine
TEMPO: (2,2,6,6-tetramethylpiperidin-1-yl)oxyl
TES: triethylsilyl
Tf: trifluoromethane sulfonyl
TFA: trifluoroacetic acid
TMANO: trimethylamine *N*-oxide
TMEDA: tetramethylethylenediamine
TMS: trimethylsilyl
Ts: tosyl
9-BBN: 9-borabicyclo[3.3.1]nonane
